# Tandem synthesis of dihydronaphthalen-1(2*H*)-one derivatives *via* aldol condensation-Diels–Alder-aromatization sequence of reactions

**DOI:** 10.1039/d5ra04673d

**Published:** 2025-09-04

**Authors:** M. Saeed Abaee, Yazdanbakhsh L. Nosood, Elaheh Akbarzadeh, Mohammad M. Mojtahedi, Ahmed Al-Harrasi

**Affiliations:** a Organic Chemistry Department, Chemistry and Chemical Engineering Research Center of Iran P.O. Box 14335-186 Tehran Iran abaee@ccerci.ac.ir; b Natural and Medical Sciences Research Center, University of Nizwa P.O. Box 33, Postal Code 616, Birkat Al Mauz Nizwa Sultanate of Oman

## Abstract

A new series of dihydronaphthalen-1(2*H*)-one derivatives were synthesized in high yields starting from commercially available 3,5,5-trimethylcyclohex-2-en-1-one 1a, aromatic aldehydes 2, and diethyl acetylenedicarboxylate. Reaction of 1a with the aldehydes produced the respective dienones 3, which could cycloadd to dialkyl acetylenedicarboxylate, either stepwise or *in situ*, under aqueous/organocatalyzed (DMAP) conditions. The respective adducts 4, were produced efficiently *via* a Diels–Alder-double bond isomerization-oxidative aromatization sequence and were characterized based on the analysis of their ^1^H and ^13^C NMR spectra.

## Abstract 2

A three-component synthesis of polysubstituted dihydronaphthalene derivatives was developed. Various derivatives of the target compounds were prepared using the three reactants under mild aqueous DMAP conditions.

## Graphical abstract

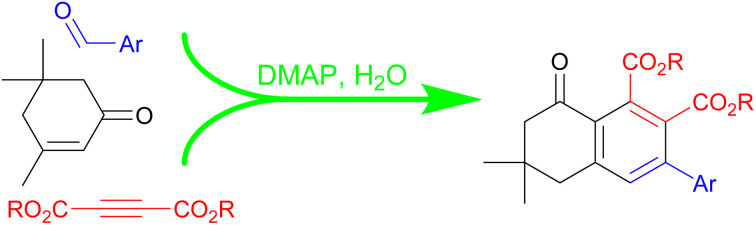

## Introduction

**Fig. 1:**
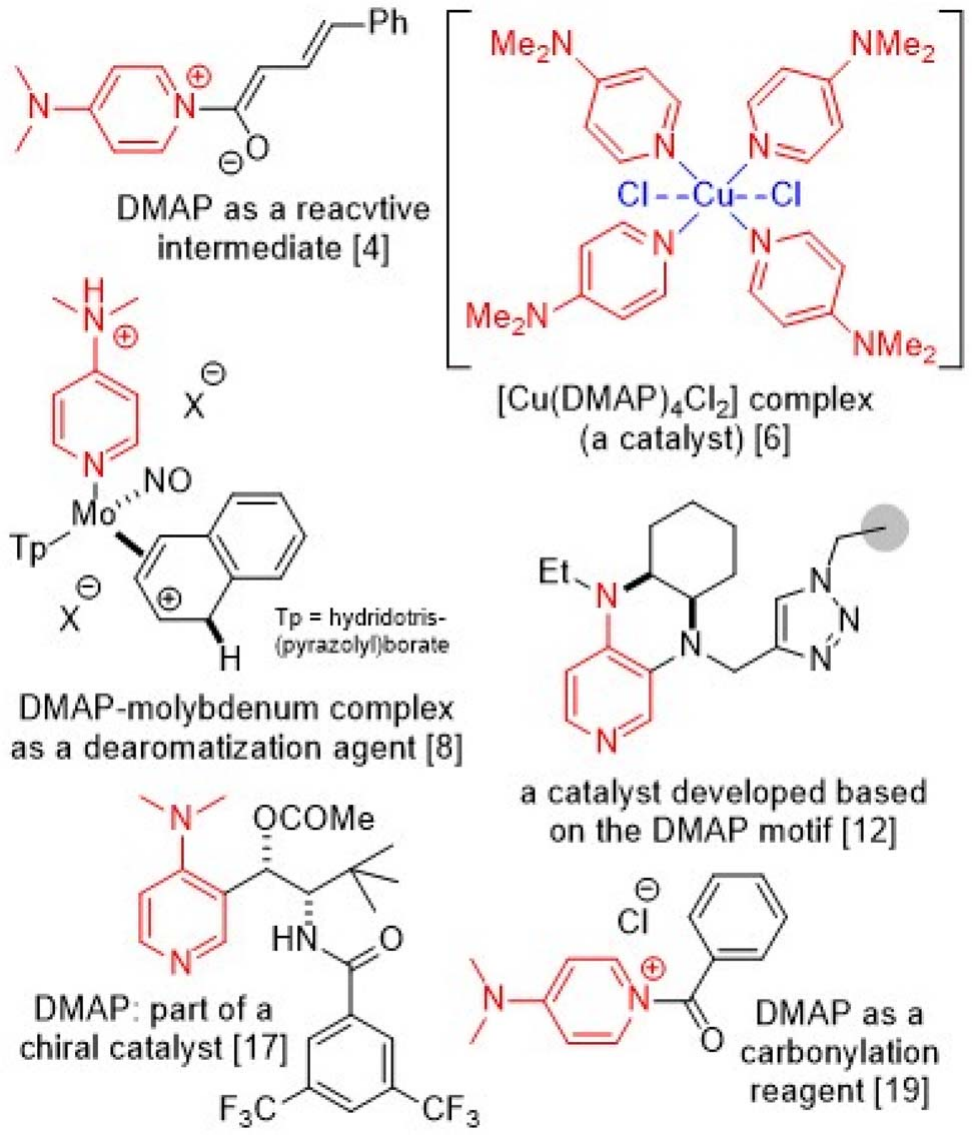
Some important synthetic roles of DMAP.

4-Dimethylaminopyridine (DMAP) is an amine with improved basicity^1^ and nucleophilicity,^2^ suitable to accelerate various organic transformations conveniently.^3^ As a catalyst, DMAP is used in the synthesis of α,β-unsaturated δ-lactones,^4^ Buchwald–Hartwig C–N coupling,^5^*N*-vinylation,^6^ [4 + 2] cycloadditions,^7^ and many other synthetic reactions.^8^ DMAP is also conveniently used in domino carbonylation-electrocyclization synthesis of imidazodipyridines^9^ and cascade synthesis of α-pyrones.^10^ In addition to usual homogeneous uses, DMAP can be used in the form of ionic liquids^11^ or immobilized derivatives.^12^ The advantages of DMAP in organic chemistry are not limited to its regular basic or nucleophilic properties and are additionally highlighted due to its uses in biological,^13^ medicinal,^14,15^ and nanoparticle disciplines,^16^ while DMAP has shown several applications in chiral catalysis^17^ and asymmetric synthesis^18^ and has got involved in synthetic protocols as a reactant^19^ or the active part of starting materials.^20^ Some illustrative cases are highlighted in Fig. 1.

**Fig. 2:**
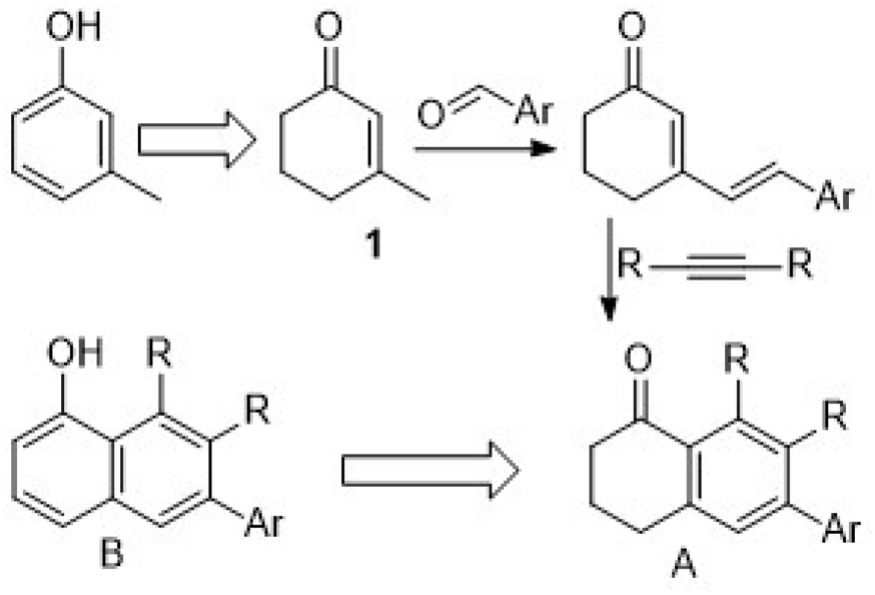
3-Methylcyclohexenone route to aromatic DA adducts.

Cyclohexenone derivatives are a group of usually inexpensive commercially available reactants for various convenient transformations to more complex products,^21–23^ including the aromatization process to their respective phenol equivalents.^24^ Among this group of compounds, the 3-methyl substituted derivative 1 would be a suitable candidate for both aromatization to the respective phenol and chain extension *via* aldol condensation with aldehydes^25^ or coupling reactions with vinylogous species^26^ at the allylic methyl position. The resulting dienes have the advantages for further synthetic manipulations through cycloaddition reactions with acetylenic dienophiles to access the respective polysubstituted dihydronaphthalen-1-one (A) or naphthol analogues (B), which in turn are useful synthons to access natural^27,28^ or synthetic^29^ structures (Fig. 2).

The [4 + 2] Diels–Alder (DA) cycloaddition is a key reaction in synthetic organic chemistry,^30^ due to efficient formation of a cyclohexene ring with up to four stereogenic centres with predictable selectivity.^31^ Incorporation of this reaction with other important organic transformations into tandem processes multiplies the efficiency of the DA cycloaddition so that many synthetic applications have arisen in recent decades as a result of DA-Hantzsch,^32^ Biginelli-DA,^33^ Heck-DA,^34^ Knoevenagel-DA,^35^ aldol condensation-DA,^36^ and other sequential reactions.^37,38^

**Scheme 1:**
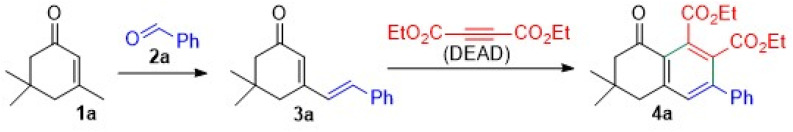
Typical stepwise reaction pathway for the conversion of 1 to 3 and 3 to 4.

We are interested in the study of DA reactions and have communicated our findings on the synthesis and cycloaddition reactions of the styrylcyclohexene diene system in recent years.^39,40^ Based on this background, we were persuaded to extend our study to embark on direct synthesis of the dihydronaphthalen skeleton starting from 3,5,5-trimethylcyclohex-2-en-1-one (1a, isophorone). We hereby disclose the synthesis of various derivatives of 4 through a tandem aldol-condensation-Diels–Alder-rearrangement-aromatization sequence of reactions under aqueous/DMAP conditions, as depicted in Scheme 1 for the reaction of 1a with benzaldehyde and the following cycloaddition step of 3a with diethyl acetylenedicarboxylate (DEAD).

## Results and discussion

We first optimized the conditions for both steps. While a mixture of 1a and benzaldehyde 2a in aqueous DMAP gave low quantities of 3a at room temperature (entry 1), treatment of the same mixture at 60 °C produced 84% of the product after 3 h (entry 2). In contrast, the yield diminished in the absence of either DMAP (entry 3) or water (entry 4). This was also the case when water was replaced with an alcohol (entries 5–6), toluene (entry 7), MeCN (entry 8), or THF (entry 9) as the medium of the reaction.^41^ Alternatively, separation of 3a and its treatment with DEAD in the same H_2_O/DMAP medium gave 4a in much higher quantities at reflux (entry 10).^42^ Treatment of the same mixture at lower temperatures was less productive (entries 11 and 12) (Table 1).

**Table 1:** Three-component optimization of the synthesis of 3a

Entry	Reactants	Conditions^a^	Temperature (^o^C)	Time (h)	Product	Yield^b^ (%)
1	1a + 2a	DMAP, H_2_O	rt	3	3a	17
2	1a + 2a	DMAP, H_2_O	60	3	3a	84
3	1a + 2a	H_2_O	60	3	3a	>5
4	1a + 2a	DMAP	60	3	3a	24
5	1a + 2a	DMAP, EtOH	60	3	3a	10
6	1a + 2a	DMAP, MeOH	60	3	3a	12
7	1a + 2a	DMAP, toluene	60	3	3a	>5
8	1a + 2a	DMAP, MeCN	60	3	3a	12
9	1a + 2a	DMAP, THF	60	3	3a	>5
10	3a + DEAD	DMAP, H_2_O	100	48	4a	81
11	3a + DEAD	DMAP, H_2_O	80	48	4a	38
12	3a + DEAD	DMAP, H_2_O	60	48	4a	22

a 10 mol% DMAP was used in all reactions.

b Isolated yields.

Having the two sets of optimized conditions, we next evaluated the generality of the method by using other derivatives of 2 (Table 2). Thus, in addition to unsubstituted aromatic aldehydes (entries 1 and 2), the H_2_O/DMAP mediated condensation of various aldehydes bearing electron releasing (entries 3 and 4) or electron withdrawing (entries 5–11) and heteroaromatic groups (entry 12) with 1a gave the respective aldol condensation products in high yields. Alternatively, isolation of 3a–l and their separate treatment with DEAD (entries 14–18 and 20–25) or dimethyl acetylenedicarboxylate (DMAD) (entries 13 and 19) in the same aqueous DMAP mixtures yielded 4a–l efficiently.

**Table 2:** Stepwise synthesis of various derivatives of 3 and 4

Entry	Conditions^a^	Reactants	Ar	Product	Yield^b^^b^ (%)
1	DMAP, H_2_O, 60 °C	1a + 2a	C_6_H_5_	3a	84
2	DMAP, H_2_O, 60 °C	1a + 2b	2-Naphthyl	3b	84
3	DMAP, H_2_O, 60 °C	1a + 2c	4-MeC_6_H_4_	3c	81
4	DMAP, H_2_O, 60 °C	1a + 2d	4-MeOC_6_H_4_	3d	87
5	DMAP, H_2_O, 60 °C	1a + 2e	3-MeOC_6_H_4_	3e	78
6	DMAP, H_2_O, 60 °C	1a + 2f	4-FC_6_H_4_	3f	82
7	DMAP, H_2_O, 60 °C	1a + 2g	4-BrC_6_H_4_	3g	80
8	DMAP, H_2_O, 60 °C	1a + 2h	4-CF_3_C_6_H_4_	3h	81
9	DMAP, H_2_O, 60 °C	1a + 2i	3-O_2_NC_6_H_4_	3i	80
10	DMAP, H_2_O, 60 °C	1a + 2j	4-ClC_6_H_4_	3j	83
11	DMAP, H_2_O, 60 °C	1a + 2k	2,6-Cl_2_C_6_H_3_	3k	75
12	DMAP, H_2_O, 60 °C	1a + 2l	2-Thienyl	3l	79
13	DMAP, H_2_O, reflux	3a + DMAD	C_6_H_5_	4a	85
14	DMAP, H_2_O, reflux	3a + DEAD	C_6_H_5_	4a′	83
15	DMAP, H_2_O, reflux	3b + DEAD	2-Naphthyl	4b	78
16	DMAP, H_2_O, reflux	3c + DEAD	4-MeC_6_H_4_	4c	83
17	DMAP, H_2_O, reflux	3d + DEAD	4-MeOC_6_H_4_	4d	86
18	DMAP, H_2_O, reflux	3e + DEAD	3-MeOC_6_H_4_	4e	77
19	DMAP, H_2_O, reflux	3f + DMAD	4-FC_6_H_4_	4f	81
20	DMAP, H_2_O, reflux	3g + DEAD	4-BrC_6_H_4_	4g	78
21	DMAP, H_2_O, reflux	3h + DEAD	4-CF_3_C_6_H_4_	4h	82
22	DMAP, H_2_O, reflux	3i + DEAD	3-O_2_NC_6_H_4_	4i	80
23	DMAP, H_2_O, reflux	3j + DEAD	4-ClC_6_H_4_	4j	81
24	DMAP, H_2_O, reflux	3k + DEAD	2,6-Cl_2_C_6_H_3_	4k	75
25	DMAP, H_2_O, reflux	3l + DEAD	2-Thienyl	4l	80

a 10 mol% DMAP was used in all reactions.

b Isolated yields.

We then evaluated the feasibility of conducting both steps in a one-pot procedure (Table 3). For this, we examined the conditions for the reaction of 1a with benzaldehyde and DEAD in a water/DMAP mixture, where initial warming of the mixture of 1a and 2a to 60 °C (1 h) and delayed addition of the dienophile DMAD to the mixture and switching the conditions to refluxing temperature produced 4a in 82% yield within 48 h (entry 1). Successful reactions of other aldehydes with either DMAD or DEAD illustrated the generality of the process by producing their respective adducts in high yields (entries 2–13).

**Table 3:** One-pot synthesis of derivatives of 4

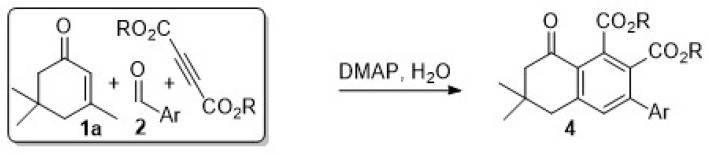					
Entry	Reactants^a^	Ar	R	Product	Yield^b^ (%)
1	1a + 2a + DMAD	C_6_H_5_	Me	4a	82
2	1a + 2a + DEAD	C_6_H_5_	Et	4a′	80
3	1a + 2b + DEAD	2-Naphthyl	Et	4b	80
4	1a + 2c + DEAD	4-MeC_6_H_4_	Et	4c	77
5	1a + 2d + DEAD	4-MeOC_6_H_4_	Et	4d	78
6	1a + 2e + DEAD	3-MeOC_6_H_4_	Et	4e	80
7	1a + 2a + DMAD	4-FC_6_H_4_	Me	4f	86
8	1a + 2f + DEAD	4-BrC_6_H_4_	Et	4g	80
9	1a + 2g + DEAD	4-CF_3_C_6_H_4_	Et	4h	80
10	1a + 2a + DMAD	3-O_2_NC_6_H_4_	Et	4i	75
11	1a + 2a + DMAD	4-ClC_6_H_4_	Et	4j	85
12	1a + 2a + DMAD	2,6-Cl_2_C_6_H_3_	Et	4k	81
13	1a + 2a + DMAD	2-Thienyl	Et	4l	80

a 10 mol% DMAP was used in all reactions.

b Isolated yields.

**Fig. 3:**
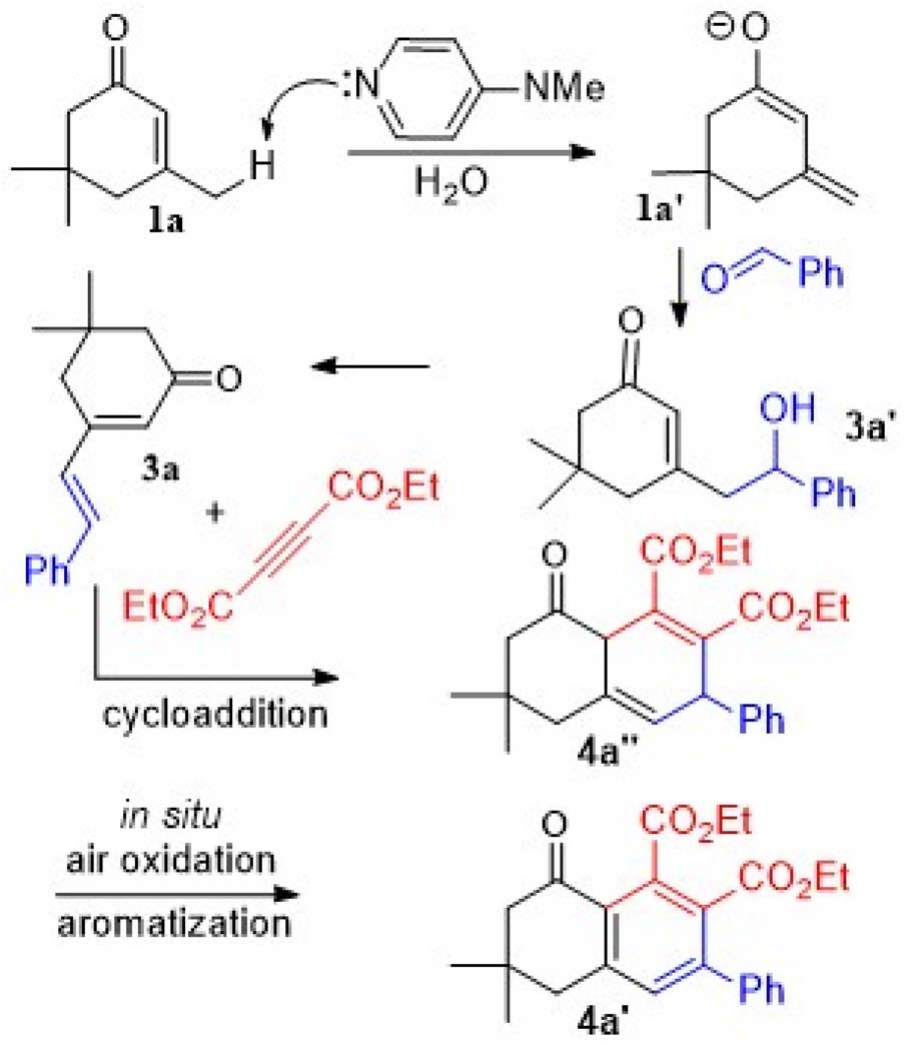
The proposed mechanism.

Based on the results obtained here and in view of the basicity of DMAP as an organocatalyst in aqueous media,^43^ a mechanism was proposed for the one-pot combination of the reactants, as shown in Fig. 3 for the reaction of benzaldehyde with 1a and DEAD. Primarily, the DMAP preferably removed the γ acidic proton (Me group) to give the dienolate intermediate (1a′). In continuation, the intermediate attacked the aldehyde to complete the aldol step, giving 3a′ and then 3a. Cycloaddition of 3a with DEAD followed by *in situ* aromatization of 4a′′ produced the final product 4a′.^44^

## Experimental

### General

All reagents were commercially available and used as received. Progress of the reactions was monitored by TLC using silica gel coated plates and EtOAc/petroleum ether mixture as the eluent. Melting points are uncorrected and obtained by Buchi Melting Point 530 apparatus. ^1^H NMR (300 MHz or 600 MHz) and ^13^C NMR (75 MHz or 150 MHz) spectra are obtained on a FT-NMR Bruker Ultra Shield™ (or Bruker DRX-600) instrument as CDCl_3_ solutions, and the chemical shifts are expressed as *δ* units with Me_4_Si as the internal standard. Chemical ionization (CI) HRMS data are obtained (with MeOH as the ionization source) using Agilent technologies 6530 Q-TOF-LC-MS. The identity of the known products was confirmed by the comparison of their ^1^H NMR and ^13^C NMR spectra with those of authentic compounds reported in the literature.^45,46^ All new products were fully characterized based on their spectral data.

### Typical synthesis of 3a

A mixture of 1a (300 μL, 2.0 mmol), benzaldehyde 2a (284 μL, 2.0 mmol), and DMAP (25 mg, 10 mol%) in water (1.0 mL) was stirred at 60 °C for 3 h. After completion of the reaction (monitored by TLC using EtOAc/hexanes (1 : 4) as the eluent), the mixture was extracted with EtOAc (3 × 5 mL), washed with brine, dried over anhydrous Na_2_SO_4_, and concentrated under reduced pressure. Product 3a (380 mg, 84%) was obtained by column chromatography fractionation of the residue using (EtOAc/hexanes, 1 : 4, v/v).

### Typical stepwise synthesis of 4a′

A mixture of 3a (1.0 mmol, 226 mg) and DEAD (255 mg, 1.5 mmol), and DMAP (13 mg, 10 mol%) in H_2_O (2.0 mL) was refluxed for 48 h, until TLC showed completion of the process. The product was extracted from the reaction mixture with EtOAc (5.0 mL), washed with brine, dried over anhydrous Na_2_SO_4_, and concentrated under reduced pressure. Product 4a′ (327 mg, 83%) was obtained by column chromatography fractionation of the residue using (EtOAc/hexanes, 1 : 4, v/v).

### Typical one-pot synthesis of 4a′

A mixture of 1a (150 μL, 1.0 mmol), benzaldehyde 2a (142 μL, 1.0 mmol), and DMAP (13 mg, 10 mol%) in water (1.0 mL) was stirred at 60 °C for 3 h. At this point, DEAD (255 mg, 1.5 mmol) was added to the mixture and the mixture was refluxed for another 36 h. The product was extracted from the reaction mixture with EtOAc (5.0 mL), washed with brine, dried over anhydrous Na_2_SO_4_, and concentrated under reduced pressure. Product 4a′ (316 mg, 80%) was obtained by column chromatography fractionation of the residue using (EtOAc/hexanes, 1 : 4, v/v).

### Spectral data of new products

#### (*E*)-5,5-dimethyl-3-(4-(trifluoromethyl)styryl)cyclohex-2-en-1-one 3h

Mp 103–105 °C; IR (KBr): 1324, 1586, 2161, 1655, 2933, 9961 cm^−1^; ^1^H NMR (300 MHz, CDCl_3_) *δ* 1.12 (s, 6H), 2.33 (s, 2H), 2.49 (s, 2H), 6.12 (s, 1H), 6.99 (s, 2H), 7.59 (d, *J* = 8.0 Hz, 2H), 7.63 (d, *J* = 8.0 Hz, 2H); ^13^C NMR (75 MHz, CDCl_3_) *δ* 28.4, 33.3, 38.9, 51.3, 125.7 (q, *J* = 28 Hz), 127.2, 128.2, 130.3, 130.7, 131.9, 133.0, 139.4, 153.7, 200.1; MS (70 eV) *m*/*z* (%), 294 (M^+^), 142, 170, 262, 277; anal. calcd for C_17_H_17_F_3_O: C, 69.38; H, 5.82. Found: C, 69.20; H, 5.79.

#### (*E*)-3-(2,6-dichlorostyryl)-5,5-dimethylcyclohex-2-en-1-one 3k

Mp 69–71 °C; IR (KBr): 1587, 1616, 1657, 2930, 2947 cm^−1^; ^1^H NMR (300 MHz, CDCl_3_*δ*) 1.12 (s, 6H), 2.32 (s, 2H), 2.50 (s, 2H), 6.06 (s, 1H), 6.94 (d, *J* = 16.5 Hz, 1H), 7.01 (d, *J* = 16.5 Hz, 1H), 7.13 (t, *J* = 8.0 Hz, 1H), 7.33 (d, *J* = 8.0 Hz, 2H); ^13^C NMR (75 MHz, CDCl_3_) *δ* 28.4, 33.2, 38.5, 51.3, 128.2, 128.4, 128.6, 128.9, 133.3, 134.4, 137.7, 153.7, 200.1; MS (70 eV) *m*/*z* (%), 294 (M^+^), 176, 204, 260; anal. calcd for C_16_H_16_Cl_2_O: C, 65.10; H, 5.46. Found: C, 64.87; H, 5.37.

#### Dimethyl 6,6-dimethyl-8-oxo-3-phenyl-5,6,7,8-tetrahydronaphthalene-1,2-dicarboxylate (4a)

Mp 151–153 °C; IR (KBr): 2949, 1739, 1694, 1588 cm^−1^; ^1^H NMR (600 MHz, CDCl_3_): 1.03 (s, 6H), 2.58 (s, 2H), 2.93 (s, 2H), 3.62 (s, 3H), 3.97 (s, 3H), 7.22 (d, *J* = 8.0 Hz, 2H), 7.29 (s, 1H), 7.56 (d, *J* = 8.0 Hz, 2H); ^13^C NMR (150 MHz, CDCl_3_): 28.1, 33.7, 43.8, 52.5, 52.6, 53.0, 122.9, 128.5, 129.6, 129.7, 131.7, 132.0, 134.2, 138.2, 144.5, 145.4, 167.4, 169.0, 196.3; ESI-HRMS (MeOH) (*m*/*z*): calcd for [C_22_H_22_O_5_ + H]^+^: 367.1540, found: 367.1545.

#### Diethyl 7,7-dimethyl-5-oxo-5,6,7,8-tetrahydro-[2,2′-binaphthalene]-3,4-dicarboxylate (4b)

Mp 124–125 °C; IR (KBr): 2969, 2255, 1726, 1241 cm^−1^; ^1^H NMR (600 MHz, CDCl_3_): 0.50 (t, *J* = 7.5 Hz, 3H), 1.14 (s, 3H), 1.17 (s, 3H), 1.41 (t, *J* = 7.5 Hz, 3H), 2.62 (q, *J* = 7.5 Hz, 2H), 2.96 (q, *J* = 7.5 Hz, 2H), 2.93 (s, 2H) 4.46 (q, *J* = 7.5 Hz, 2H), 7.34 (dd, *J* = 8.0 Hz, 1H), 7.36 (s, 1H); 7.45 (dd, *J* = 8.0, 8.0 Hz, 1H), 7.50 (dd, *J* = 8.0, 8.0 Hz, 2H), 7.59 (d, *J* = 8.0 Hz, 1H), 7.89 (d, *J* = 8.0 Hz, 1H), 7.91 (d, *J* = 8.0 Hz, 1H); ^13^C NMR (150 MHz, CDCl_3_): 13.0, 13.9, 28.0, 28.3, 33.8, 43.8, 52.7, 61.1, 61.9, 125.0, 125.7, 126.1, 126.3, 126.4, 128.2, 128.4, 128.7, 131.3, 131.5, 133.2, 133.3, 134.4, 137.4, 144.9, 145.0, 166.4, 168.7196.4; ESI-HRMS (MeOH) (*m*/*z*): calcd for [C_28_H_28_O_5_ + H]^+^: 445.2009, found: 445.2010.

#### Diethyl 3-(4-fluorophenyl)-6,6-dimethyl-8-oxo-5,6,7,8-tetrahydronaphthalene-1,2-dicarboxylate (4f)

Mp 93–95 °C; IR (KBr): 3062, 1729, 1233 cm^−1^; ^1^H NMR (600 MHz, CDCl_3_): 1.02 (t, *J* = 7.0 Hz, 3H). 1.11 (s, 6H), 1.40 (t, *J* = 7.0 Hz, 3H), 2.58 (s, 2H), 2.93 (s, 2H), 4.07 (q, *J* = 7.0 Hz, 2H), 4.44 (q, *J* = 7.0 Hz, 2H), 7.11–7.14 (m, 2H), 7.28 (s, 1H), 7.33–7.35 (m, 2H); ^13^C NMR (150 MHz, CDCl_3_): 13.6, 13.9, 28.1, 33.8, 43.8, 52.6, 61.8, 62.0, 115.5 (d, *J* = 21.0 Hz), 128.3, 128.8 (d, *J* = 9.0 Hz), 130.3, 131.9, 134.2, 135.5, 144.8 (d, *J* = 84.0 Hz), 144.4 (d, *J* = 247.5 Hz), 145.1 (d, *J* = 211.5 Hz), 196.3; ESI-HRMS (MeOH) (*m*/*z*): calcd for [C_24_H_25_FO_5_ + H]^+^: 413.1759, found: 413.1759.

#### Diethyl 3-(4-bromophenyl)-6,6-dimethyl-8-oxo-5,6,7,8-tetrahydronaphthalene-1,2-dicarboxylate (4g)

Mp 133–135 °C; IR (KBr): 2981, 1737, 1591 cm^−1^; ^1^H NMR (300 MHz, CDCl_3_): 0.94 (t, *J* = 7.0 Hz, 3H). 1.04 (s, 6H), 1.31 (t, *J* = 7.0 Hz, 3H), 2.49 (s, 2H), 2.84 (s, 2H), 4.00 (q, *J* = 7.0 Hz, 2H), 4.35 (q, *J* = 7.0 Hz, 2H), 7.14 (d, *J* = 7.5 Hz, 2H), 7.18 (s, 1H), 7.45 (d, *J* = 7.5 Hz, 2H); ^13^C NMR (75 MHz, CDCl_3_): 13.6, 13.9, 28.1, 33.8, 43.8, 52.6, 61.8, 62.0, 122.7, 128.5, 129.7, 130.1, 131.6, 131.7, 134.3, 138.4, 144.4, 145.1, 166.9, 167.4, 167.1, 196.2; ESI-HRMS (MeOH) (*m*/*z*): calcd for [C_24_H_25_BrO_5_ + H]^+^: 473.0958, found: 473.0964.

#### Diethyl 6,6-dimethyl-8-oxo-3-(4-(trifluoromethyl)phenyl)-5,6,7,8-tetrahydronaphthalene-1,2-dicarboxylate (4h)

IR (KBr): 2971, 1728, 1592 cm^−1^; ^1^H NMR (300 MHz, CDCl_3_): 0.96 (t, *J* = 7.0 Hz, 3H). 1.12 (s, 6H), 1.40 (t, *J* = 7.0 Hz, 3H), 2.58 (s, 2H), 2.93 (s, 2H), 4.04 (q, *J* = 7.0 Hz, 2H), 4.44 (q, *J* = 7.0 Hz, 2H), 7.30 (s, 1H), 7.46 (d, *J* = 7.5 Hz, 2H), 7.69 (d, *J* = 7.5 Hz, 2H); ^13^C NMR (75 MHz, CDCl_3_): 13.2, 13.6, 27.8, 33.5, 43.6, 52.3, 61.5, 61.8, 122.0 (q, *J* = 28.0 Hz), 125.1, 128.2, 128.5, 129.8, 130.0, 130.4, 131.6, 134.2, 142.9, 143.8, 145.1, 167.1 (d, *J* = 125.0 Hz), 195.9; ESI-HRMS (MeOH) (*m*/*z*): calcd for [C_25_H_25_F_3_O_5_ + H]^+^: 463.1727, found: 463.1732.

#### Diethyl 6,6-dimethyl-3-(3-nitrophenyl)-8-oxo-5,6,7,8-tetrahydronaphthalene-1,2-dicarboxylate (4i)

Mp 140–141 °C; IR (KBr): 2949, 1738, 1587, 1237 cm^−1^; ^1^H NMR (300 MHz, CDCl_3_): 1.03 (t, *J* = 7.0 Hz, 3H). 1.12 (s, 6H), 1.39 (t, *J* = 7.0 Hz, 3H), 2.58 (s, 2H), 2.94 (s, 2H), 4.08 (q, *J* = 7.0 Hz, 2H), 4.43 (q, *J* = 7.0 Hz, 2H), 7.32 (s, 1H), 7.60 (dd, *J* = 7.5, 7.5 Hz, 1H), 7.67 (dt, *J* = 1.5, 7.5 Hz, 1H), 8.20–8.30 (m, 2H); ^13^C NMR (75 MHz, CDCl_3_): 13.6, 13.9, 28.1, 33.8, 43.8, 52.5, 61.9, 62.1, 123.0, 123.1, 129.1, 129.4, 129.9, 132.0, 134.1, 134.6, 141.0, 142.9, 145.5, 148.1, 166.3, 168.1, 196.1; ESI-HRMS (MeOH) (*m*/*z*): calcd for [C_24_H_25_NO_7_ + H]^+^: 440.1704, found: 440.1709.

#### Diethyl 3-(2,6-dichlorophenyl)-6,6-dimethyl-8-oxo-5,6,7,8-tetrahydronaphthalene-1,2-dicarboxylate (4k)

Mp 182–185 °C; IR (KBr): 2972, 1729, 1593 cm^−1^; ^1^H NMR (300 MHz, CDCl_3_): 0.95 (t, *J* = 7.0 Hz, 3H). 1.13 (s, 6H), 1.41 (t, *J* = 7.0 Hz, 3H), 2.60 (s, 2H), 2.94 (s, 2H), 4.05 (q, *J* = 7.0 Hz, 2H), 4.45 (q, *J* = 7.0 Hz, 2H), 7.16 (s, 1H), 7.28 (dd, *J* = 7.5, 7.5 Hz, 1H), 7.39 (dd, *J* = 1.5, 7.5 Hz, 2H); ^13^C NMR (75 MHz, CDCl_3_): 13.4, 13.9, 28.1, 33.8, 43.8, 52.7, 61.4, 61.9, 127.1, 129.2, 129.4, 129.5, 132.6, 134.1, 135.4, 138.0, 141.5, 145.9, 165.0, 168.5, 196.4; ESI-HRMS (MeOH) (*m*/*z*): calcd for [C_24_H_24_Cl_2_O_5_ + H]^+^: 463.1074, found: 463.1079.

## Conclusions

In summary, we introduced a novel three-component method for the synthesis of a series of polysubstituted dihydronaphthalene derivatives. Both the diene formation and the cycloaddition steps were performed sequentially in the same pot using all three required reactants. The process was easy to operate by using an aqueous DMAP medium with no extra catalyst or additive needed. Additionally, a single product was obtained for each of the reactions in a good overall yield.

## Author contributions

M. S. Abaee conceived and designed the work. Y. L. Nosood and E. Akbarzadeh performed the experiments and collected data. M. M. Mojtahedi reviewed the draft and performed the literature survey. A. Al-Harrasi conducted some of the analyses. All authors analyzed the data, discussed the results, and reviewed the manuscript.

## Conflicts of interest

There are no conflicts to declare.

## Supplementary Material

RA-015-D5RA04673D-s001

## Data Availability

The data supporting this article have been included as part of the SI. Supplementary information: Spectra of new compounds (^1^H NMR, ^13^C NMR). See DOI: https://doi.org/10.1039/d5ra04673d.
