# Supplementary material for: Tandem synthesis of dihydronaphthalen-1(2H)-one derivatives via aldol condensation-Diels–Alder-aromatization sequence of reactions
Source: RSC Adv. 2025 Sep 4;15(38):31806–11. doi: 10.1039/d5ra04673d (PMC12409611; doi:10.1039/d5ra04673d)
Supplement: RA-015-D5RA04673D-s001 [file RA-015-D5RA04673D-s001.pdf]

Supplementary Information (SI) for RSC Advances.
This journal is © The Royal Society of Chemistry 2025

Supporting Information

(NMR spectra of all products)

Tandem synthesis of dihydronaphthalen-1(2*H*)-one derivatives via aldol condensation-Diels-Alder-aromatization sequence of reactions

M. Saeed Abaee, Yazdanbakhsh L. Nosood, Elaheh Akbarzadeh, Mohammad M. Mojtahedi, and Ahmed Al-Harrasi

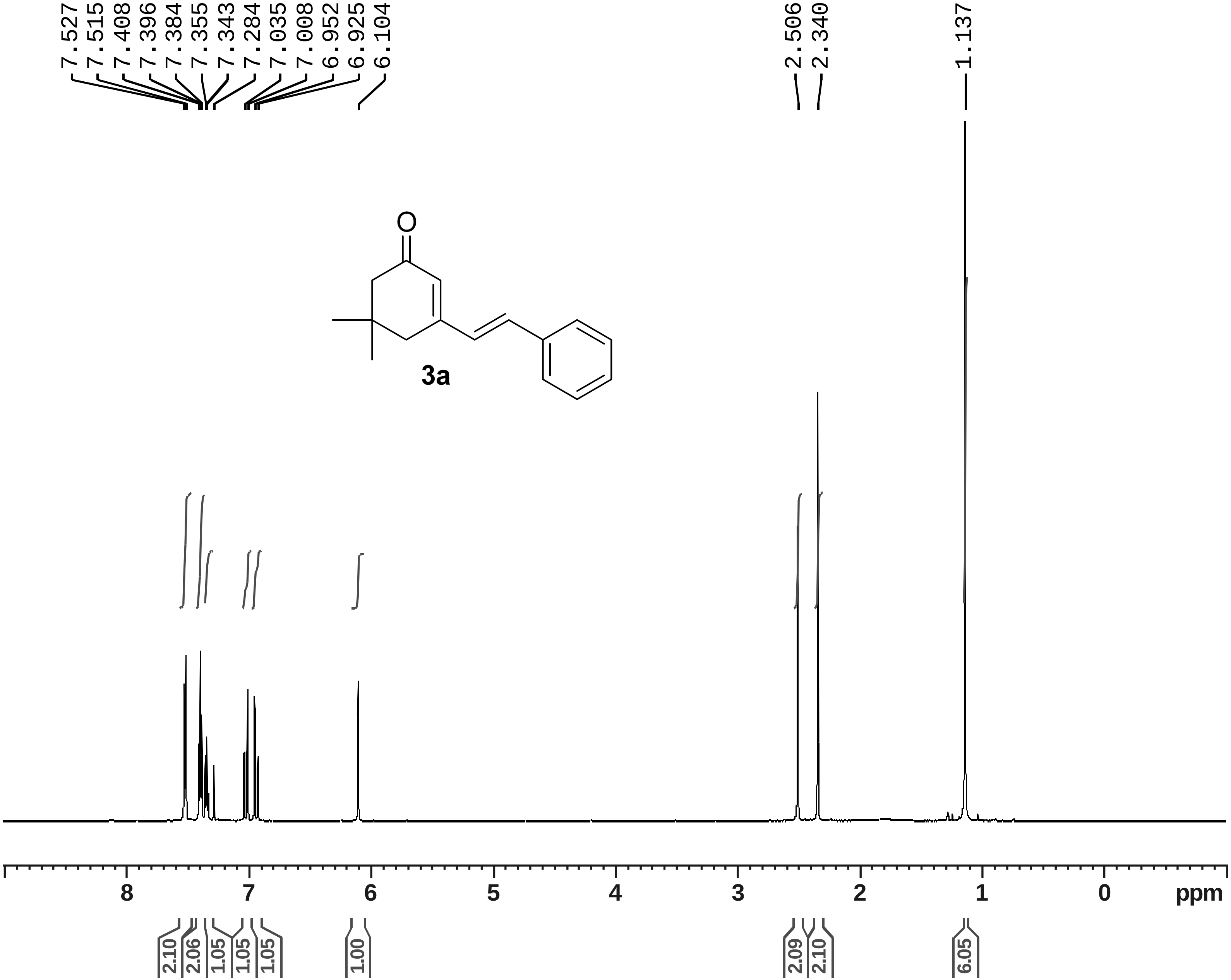
7.527
7.515
7.408
7.396
7.384
7.355
7.343
7.284
7.035
7.008
6.952
6.925
6.104
2.506
2.340
1.137
O
3a
8
7
6
5
4
3
2
1
0
ppm
2.10
2.06
1.05
1.05
1.05
1.00
2.09
2.10
6.05

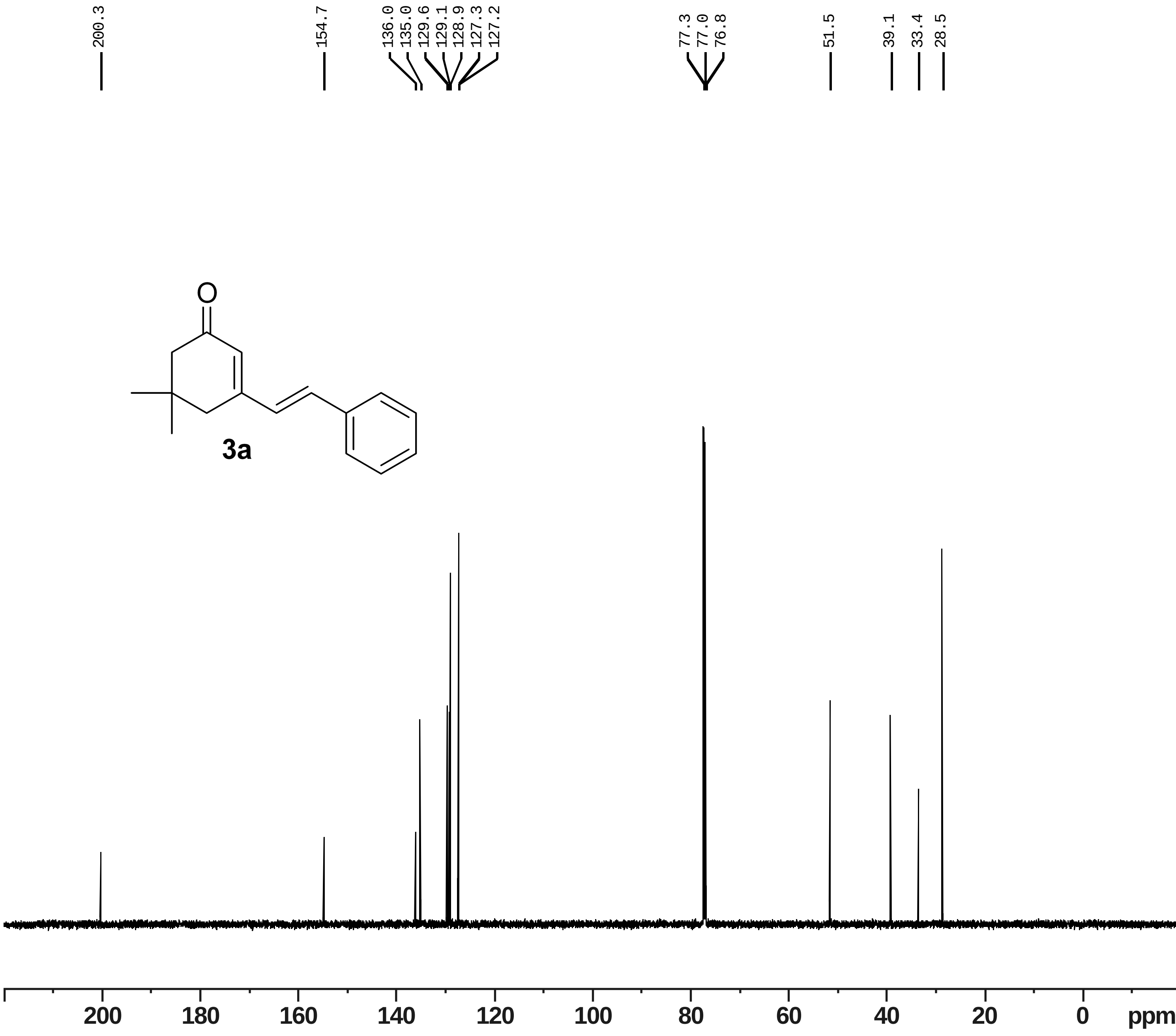
200.3
154.7
136.0
135.0
129.6
129.1
128.9
127.3
127.2
77.3
77.0
76.8
51.5
39.1
33.4
28.5
O
3a
200
180
160
140
120
100
80
60
40
20
0
ppm

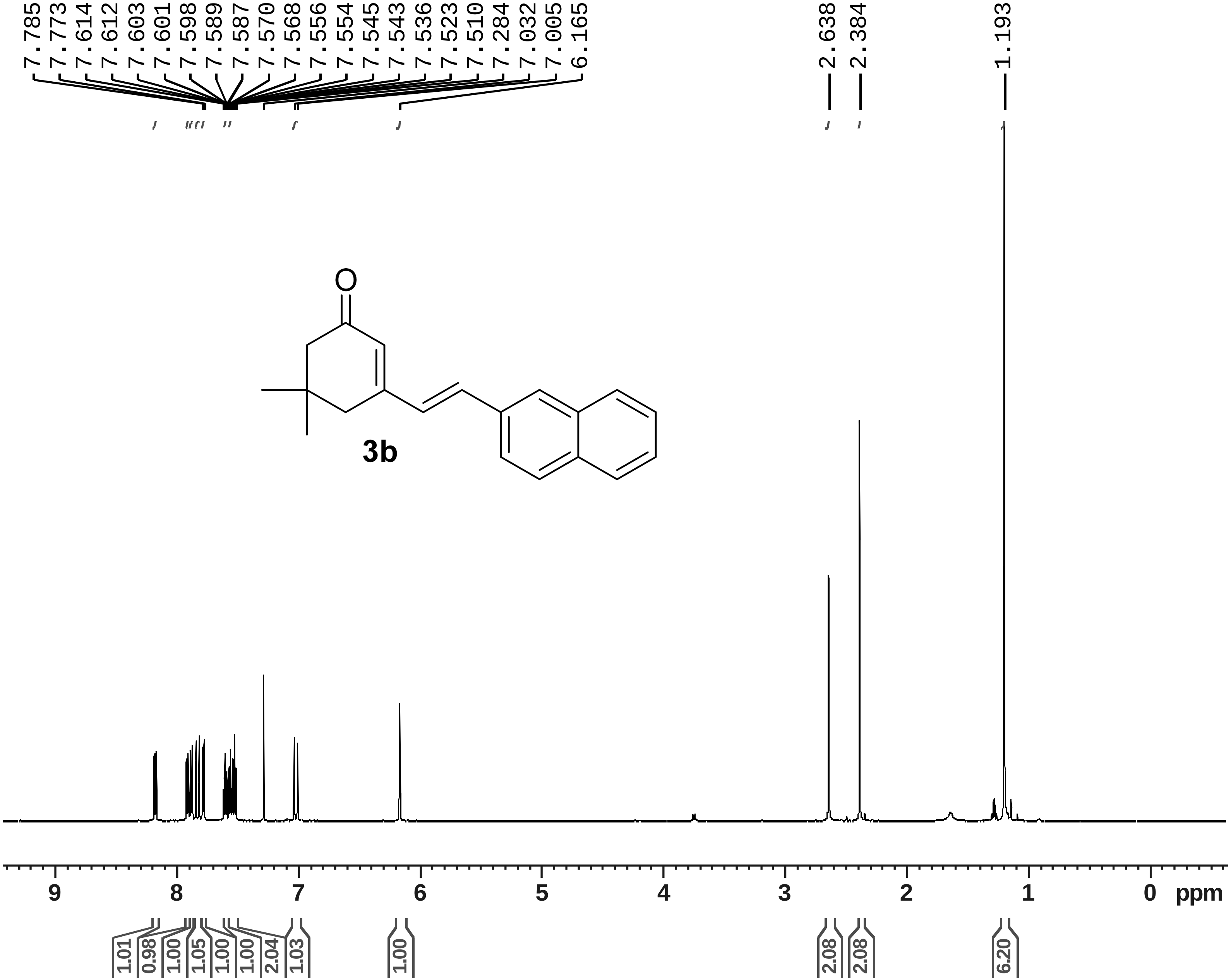
7.785
7.773
7.614
7.612
7.603
7.601
7.598
7.589
7.587
7.570
7.568
7.556
7.554
7.545
7.543
7.536
7.523
7.510
7.284
7.032
7.005
6.165
2.638
2.384
1.193
O
3b
9
8
7
6
5
4
3
2
1
0
ppm
1.01
0.98
1.00
1.05
1.00
1.00
2.04
1.03
1.00
2.08
2.08
6.20

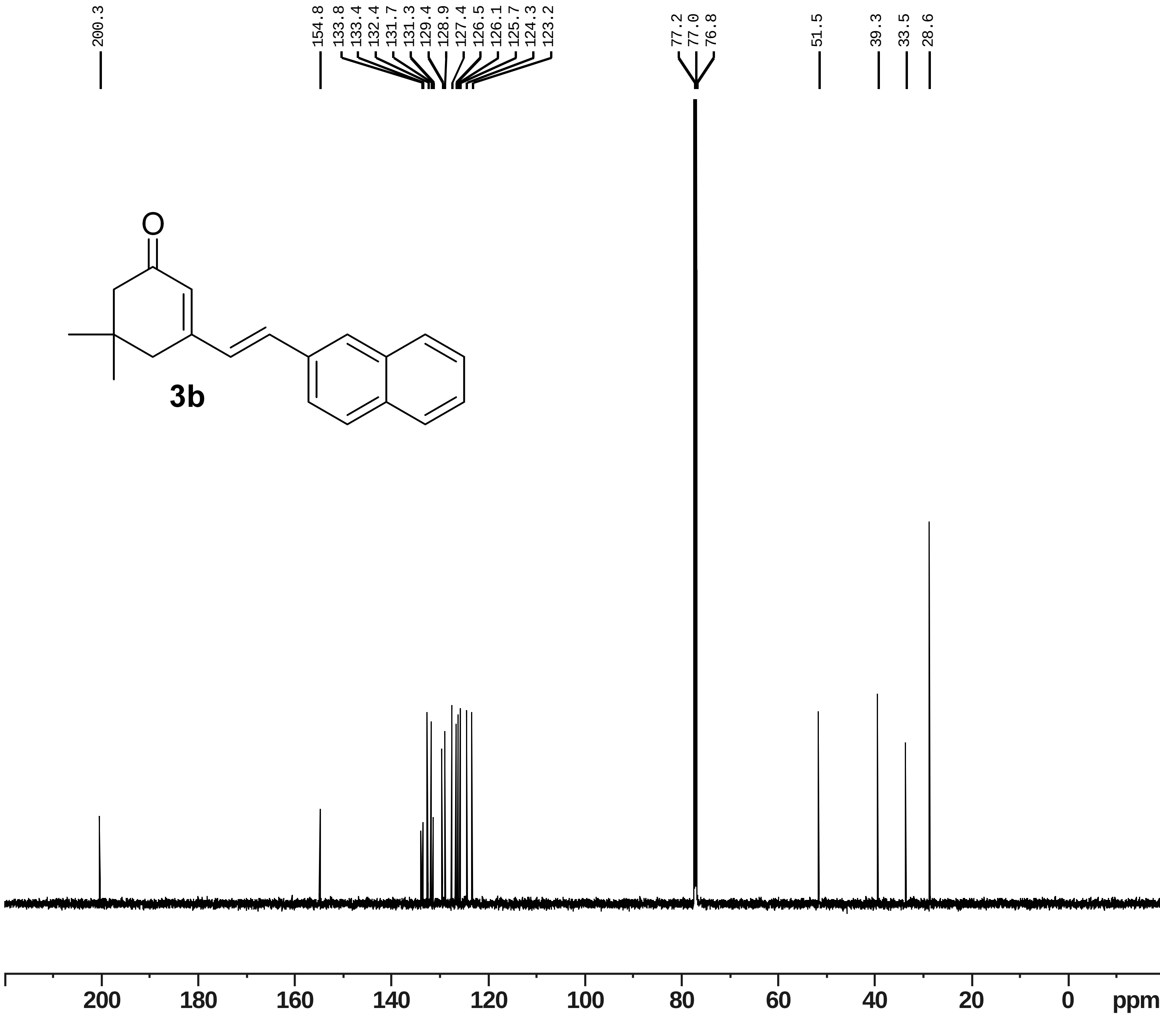
200.3
154.8
133.8
133.4
132.4
131.7
131.3
129.4
128.9
127.4
126.5
126.1
125.7
124.3
123.2
77.2
77.0
76.8
51.5
39.3
33.5
28.6
O
3b
200
180
160
140
120
100
80
60
40
20
0
ppm

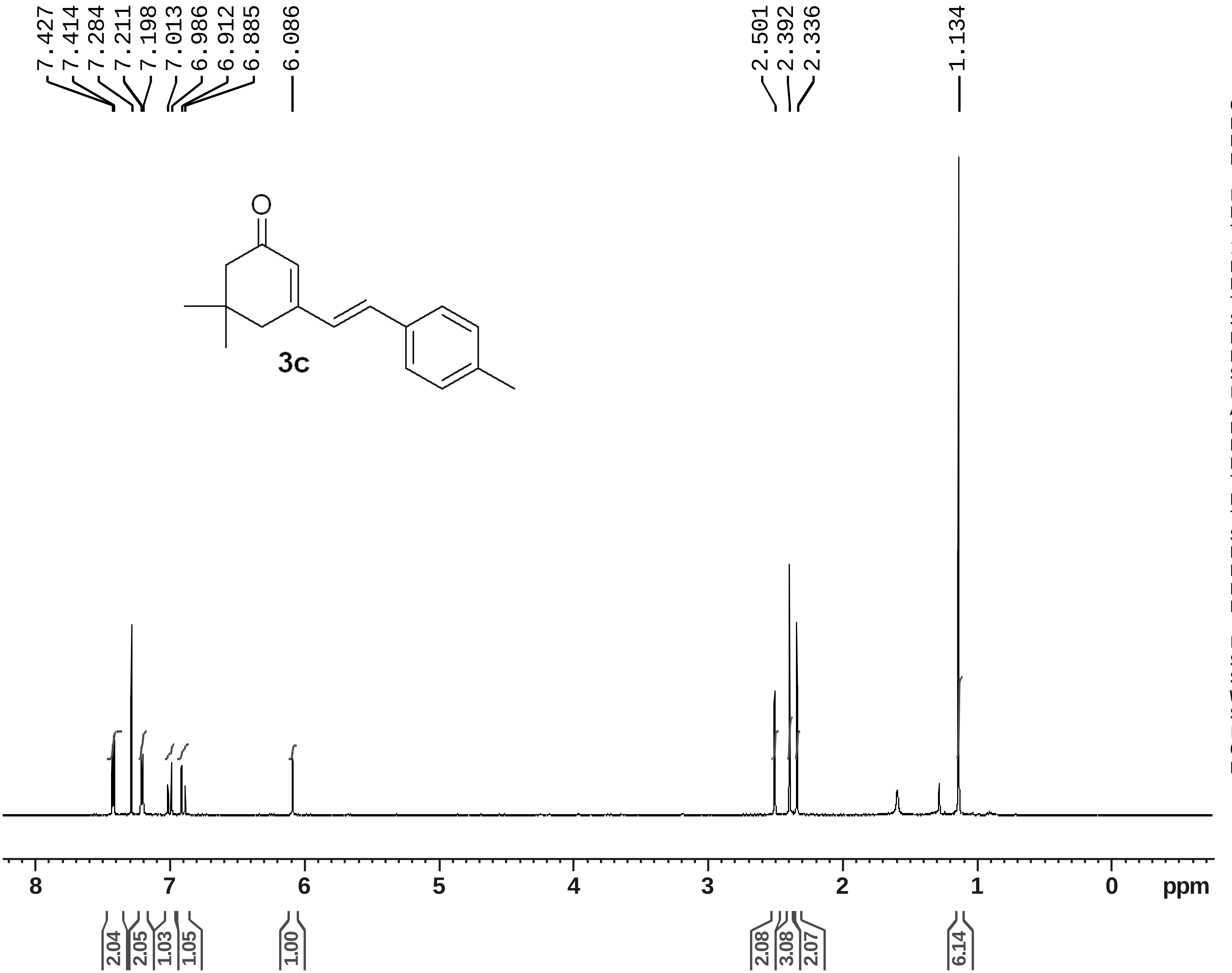
7.427
7.414
7.284
7.211
7.198
7.013
6.986
6.912
6.885
6.086
2.501
2.392
2.336
1.134
O
3c
8
7
6
5
4
3
2
1
0
ppm
2.04
2.05
1.03
1.05
1.00
2.08
3.08
2.07
6.14

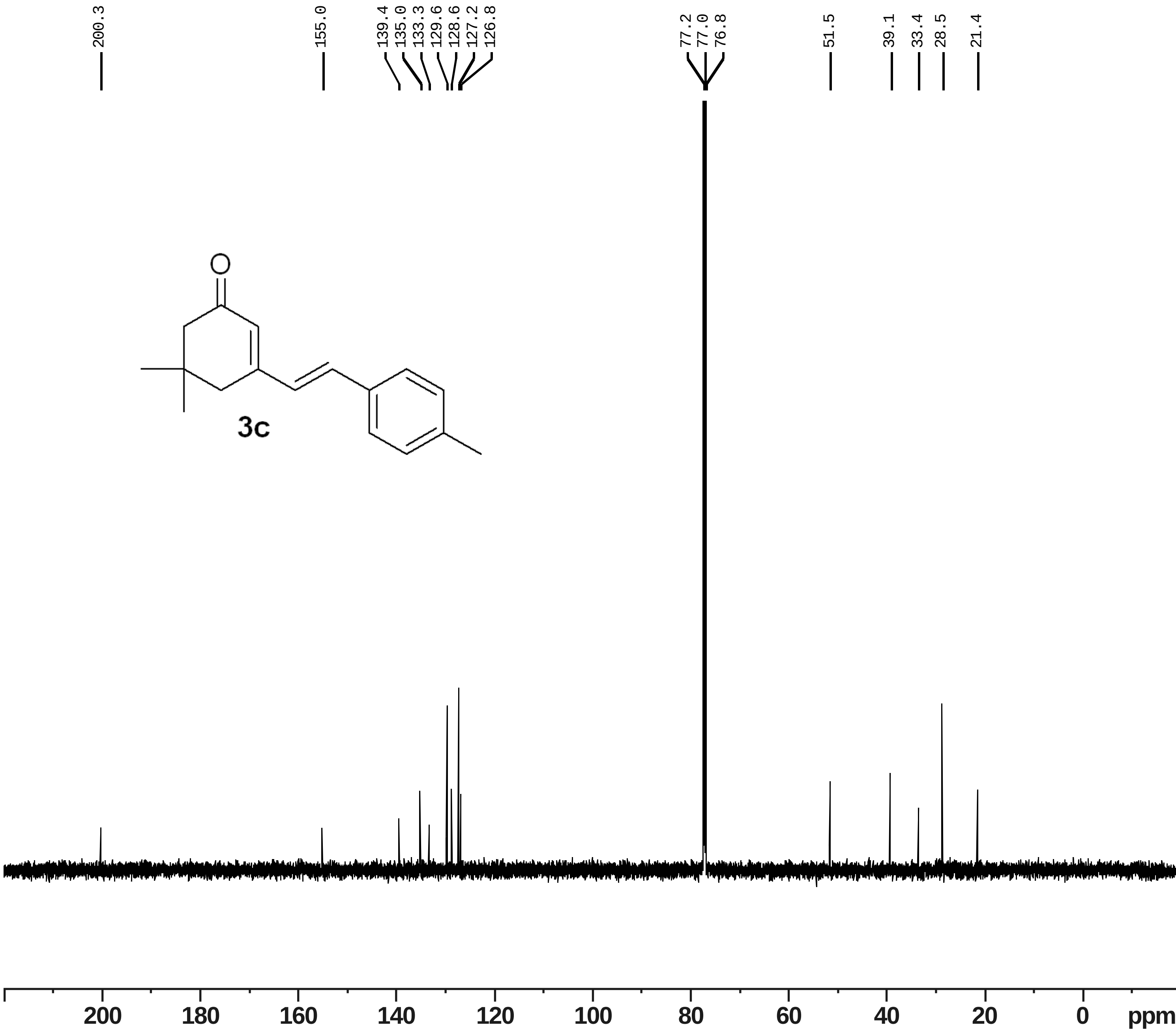
200.3
155.0
139.4
135.0
133.3
129.6
128.6
127.2
126.8
77.2
77.0
76.8
51.5
39.1
33.4
28.5
21.4
O
3c
200
180
160
140
120
100
80
60
40
20
0
ppm

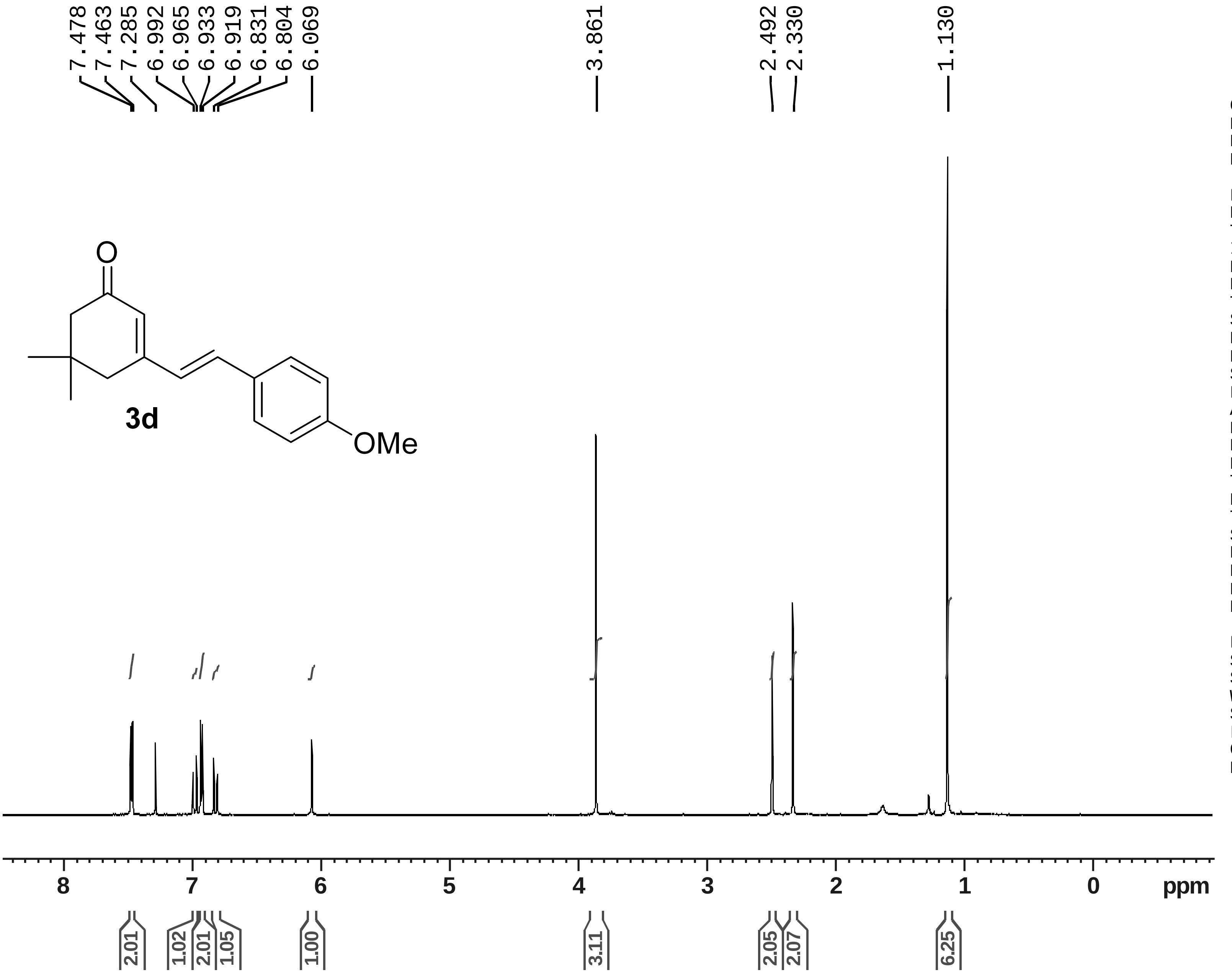

7.478
7.463
7.285
6.992
6.965
6.933
6.919
6.831
6.804
6.069
3.861
2.492
2.330
1.130
O
OMe
3d
8
7
6
5
4
3
2
1
0
ppm
2.01
1.02
2.01
1.05
1.00
3.11
2.05
2.07
6.25

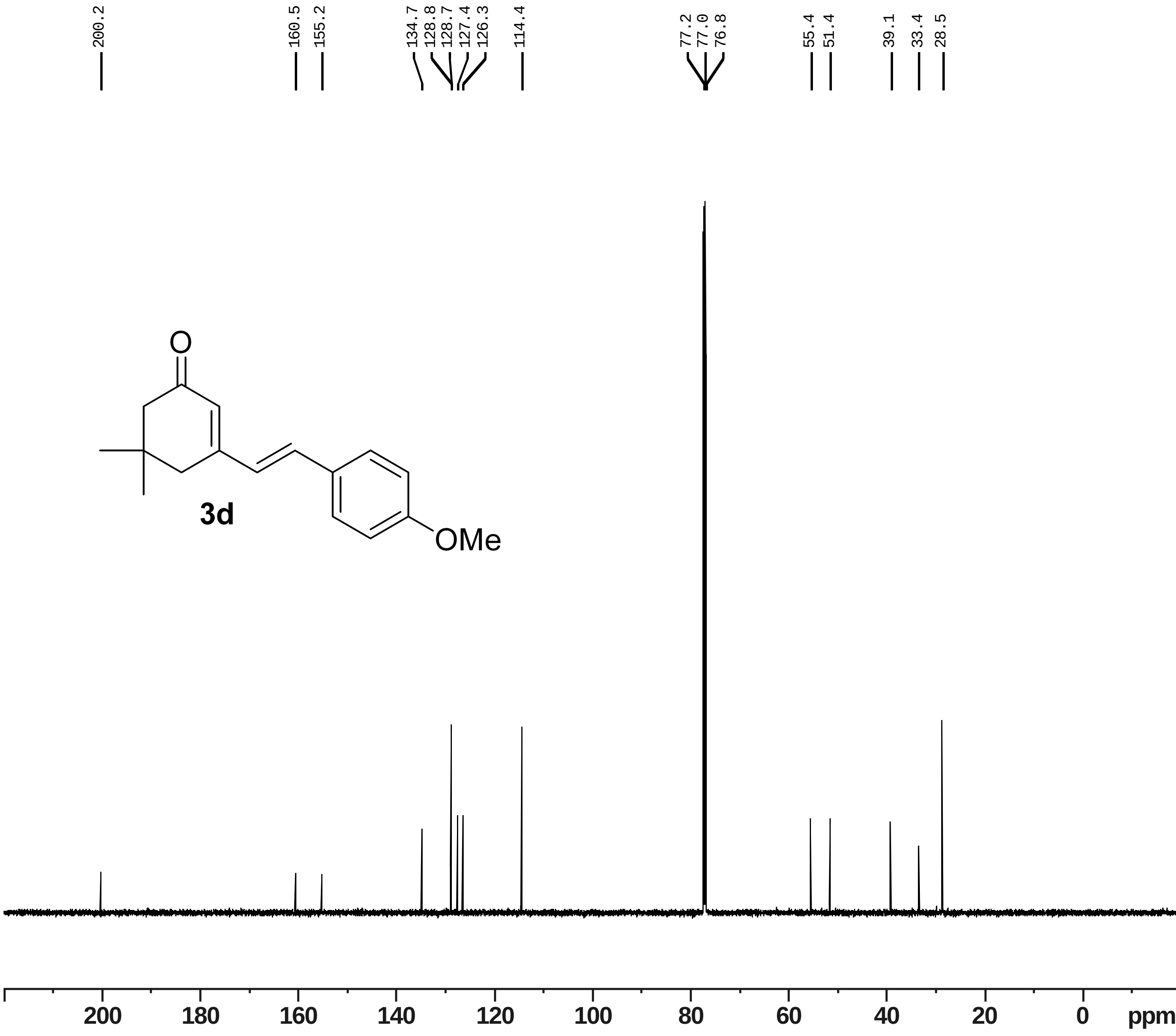
200.2
160.5
155.2
134.7
128.8
128.7
127.4
126.3
114.4
77.2
77.0
76.8
55.4
51.4
39.1
33.4
28.5
O
OMe
3d
200
180
160
140
120
100
80
60
40
20
0
ppm

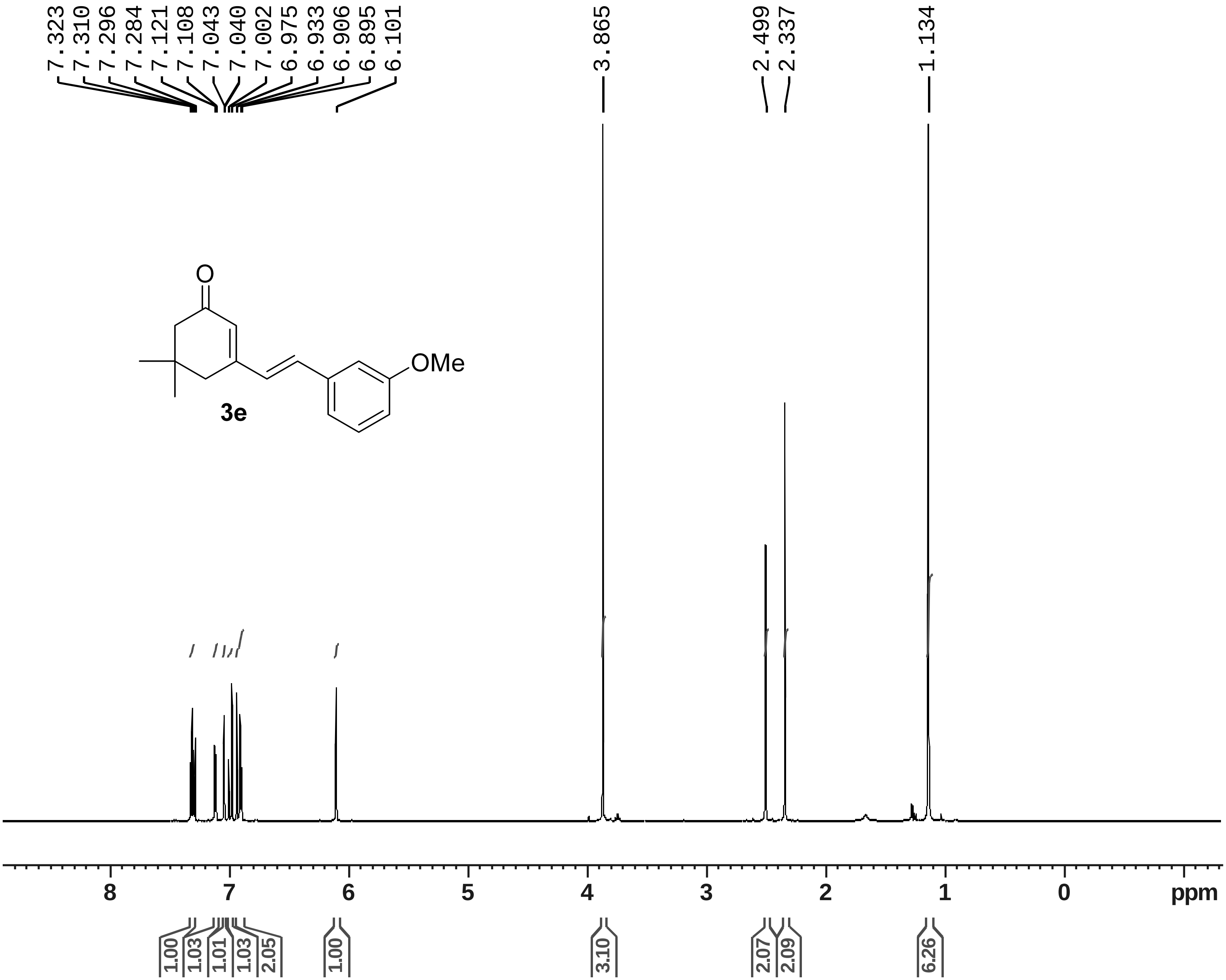
7.323
7.310
7.296
7.284
7.121
7.108
7.043
7.040
7.002
6.975
6.933
6.906
6.895
6.101
3.865
2.499
2.337
1.134
O
OMe
3e
8
7
6
5
4
3
2
1
0
ppm
1.00
1.03
1.01
1.03
2.05
1.00
3.10
2.07
2.09
6.26

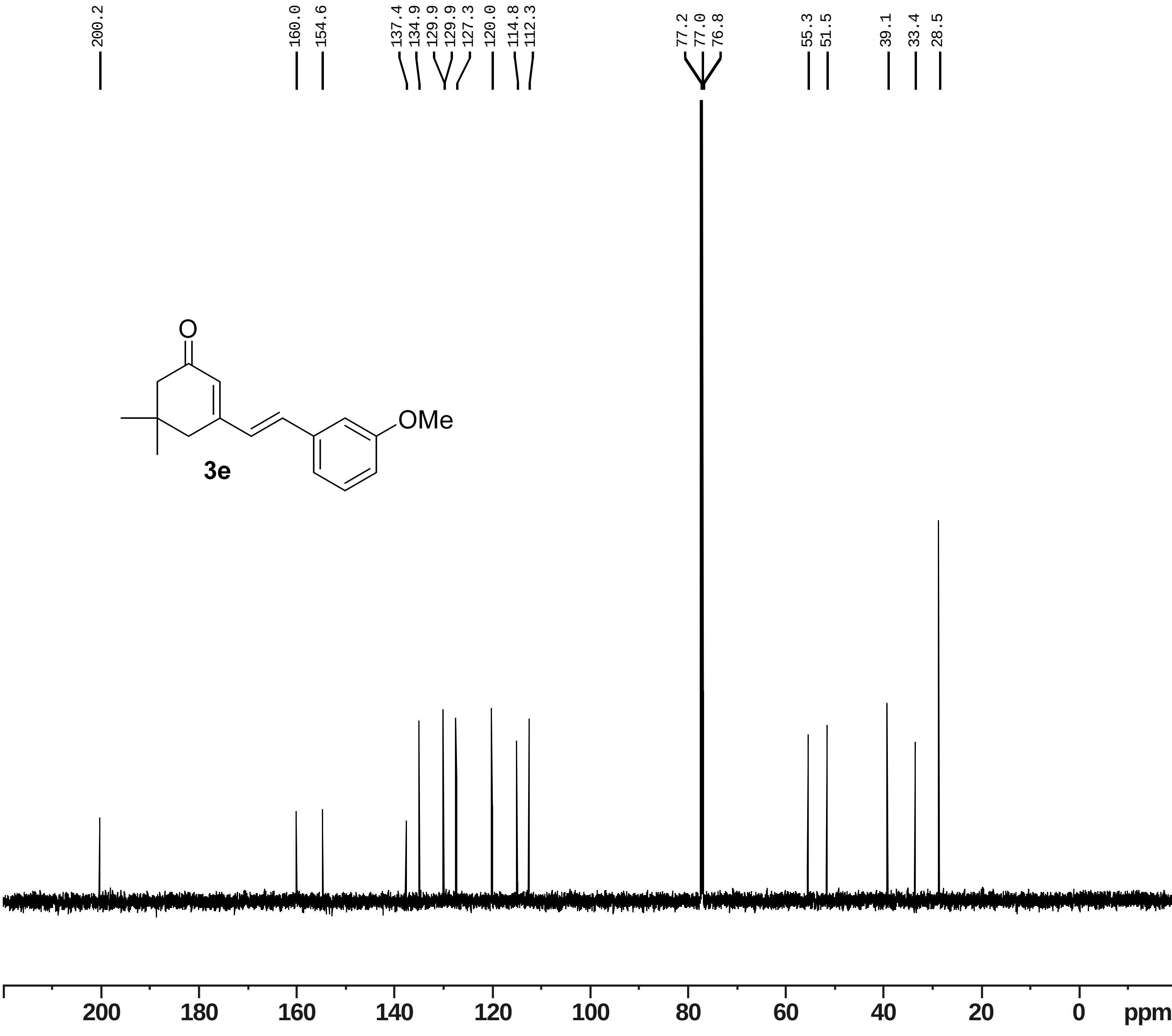
200.2
160.0
154.6
137.4
134.9
129.9
129.9
127.3
120.0
114.8
112.3
77.2
77.0
76.8
55.3
51.5
39.1
33.4
28.5
O
OMe
3e
200
180
160
140
120
100
80
60
40
20
0
ppm

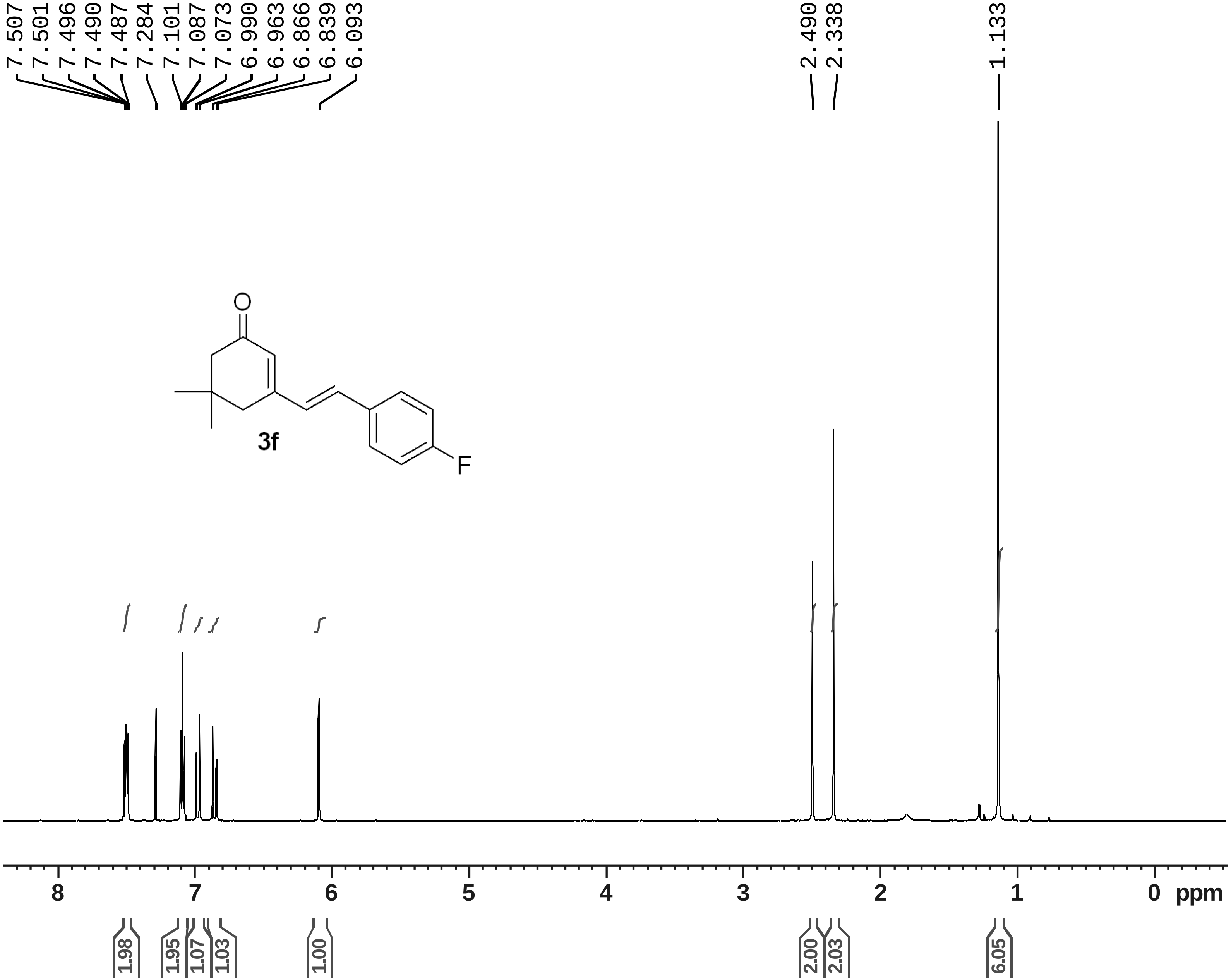
7.507
7.501
7.496
7.490
7.487
7.284
7.101
7.087
7.073
6.990
6.963
6.866
6.839
6.093
2.490
2.338
1.133
O
F
3f
8
7
6
5
4
3
2
1
0
ppm
1.98
1.95
1.07
1.03
1.00
2.00
2.03
6.05

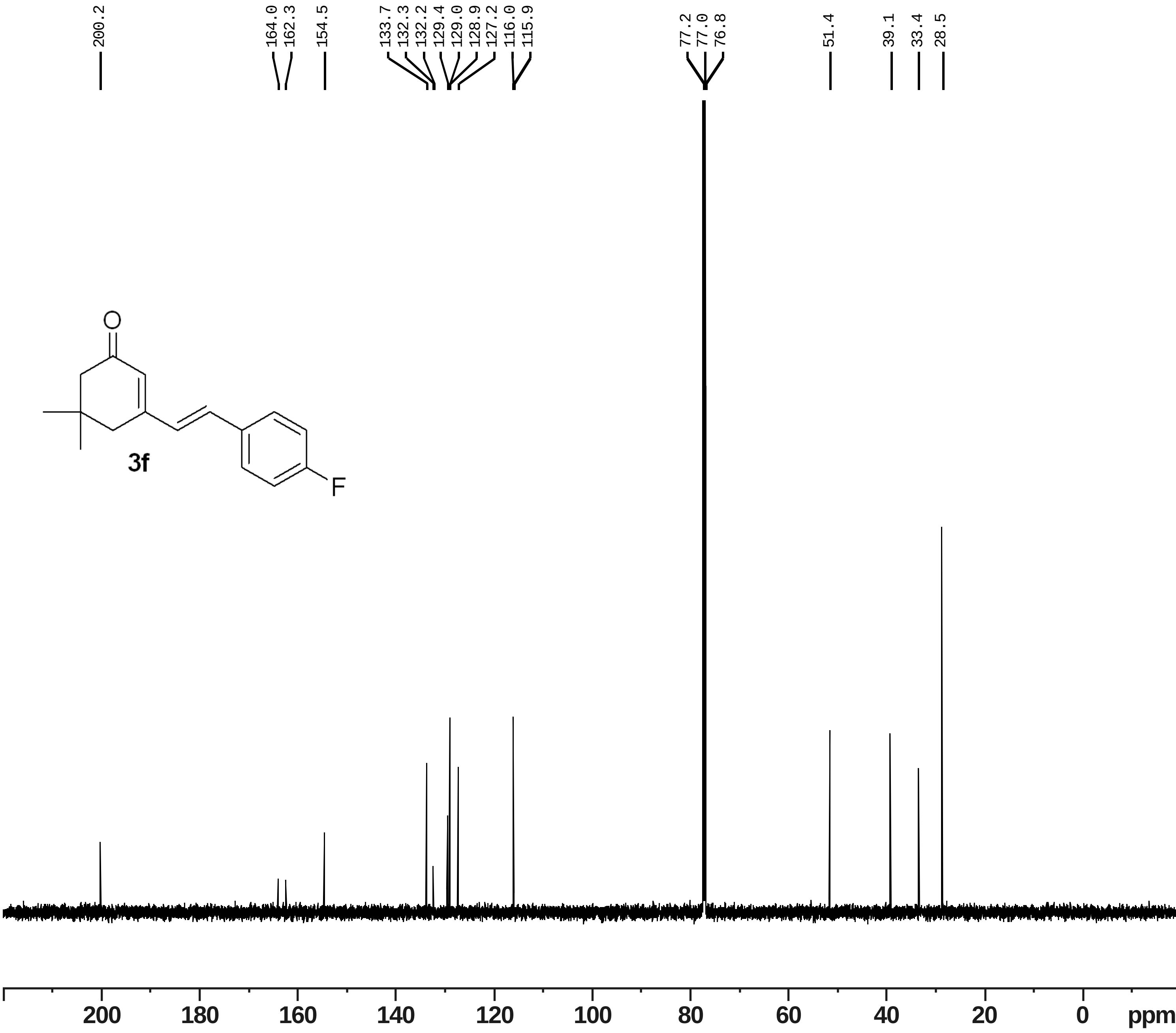
200.2
164.0
162.3
154.5
133.7
132.3
132.2
129.4
129.0
128.9
127.2
116.0
115.9
77.2
77.0
76.8
51.4
39.1
33.4
28.5
O
F
3f
200
180
160
140
120
100
80
60
40
20
0
ppm

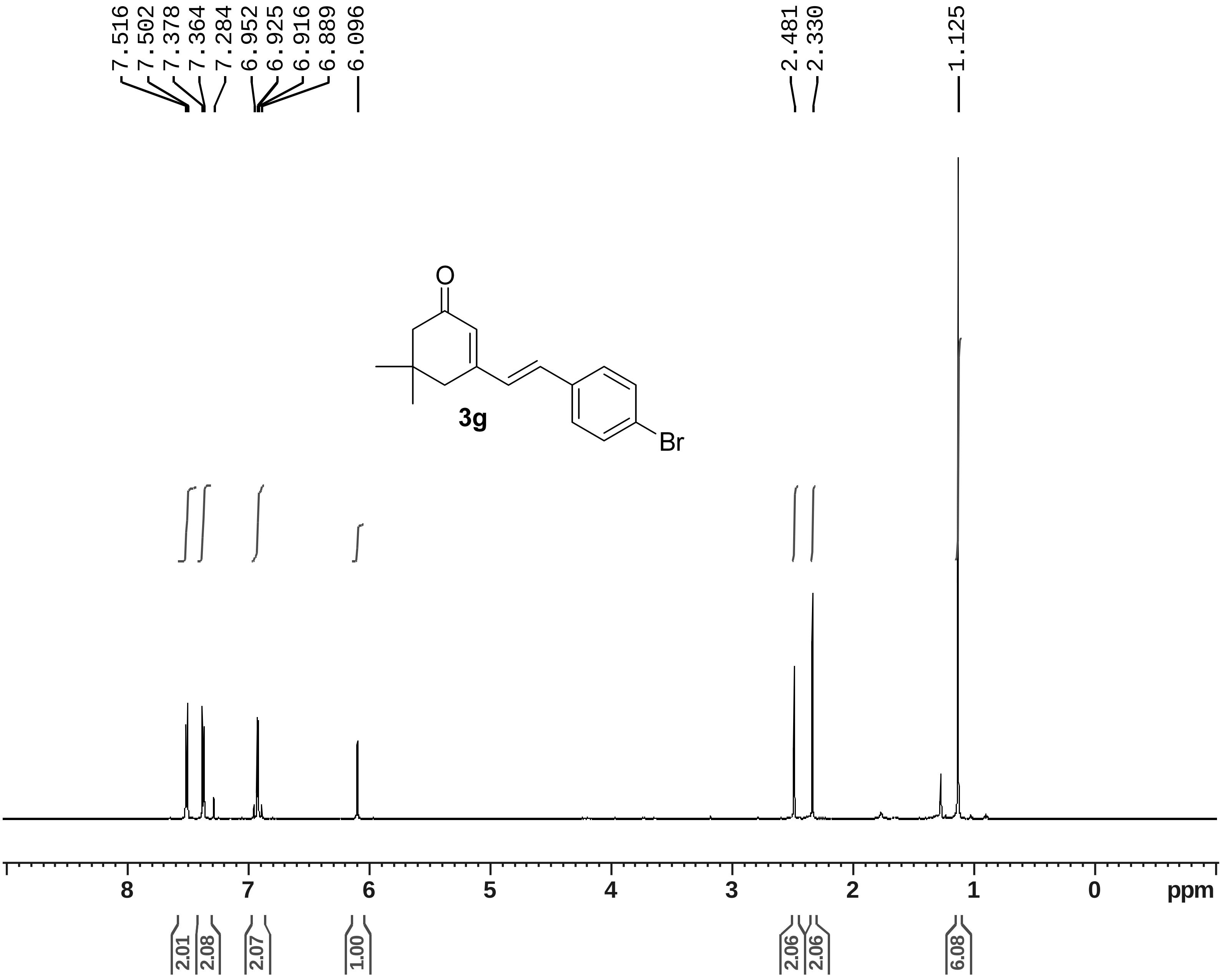
7.516
7.502
7.378
7.364
7.284
6.952
6.925
6.916
6.889
6.096
2.481
2.330
1.125
O
Br
3g
8
7
6
5
4
3
2
1
0
ppm
2.01
2.08
2.07
1.00
2.06
2.06
6.08

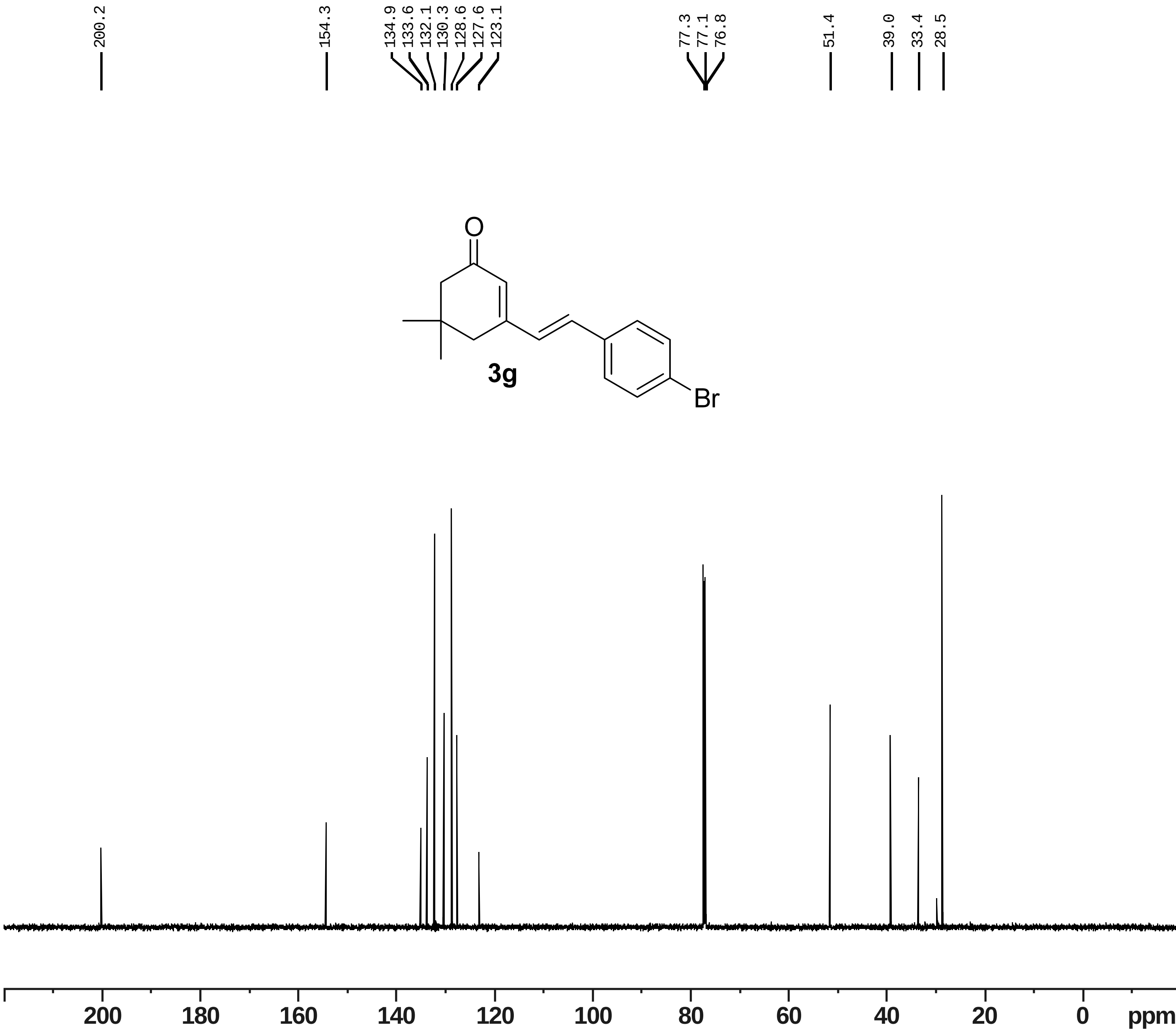
200.2
154.3
134.9
133.6
132.1
130.3
128.6
127.6
123.1
77.3
77.1
76.8
51.4
39.0
33.4
28.5
O
Br
3g
200
180
160
140
120
100
80
60
40
20
0
ppm

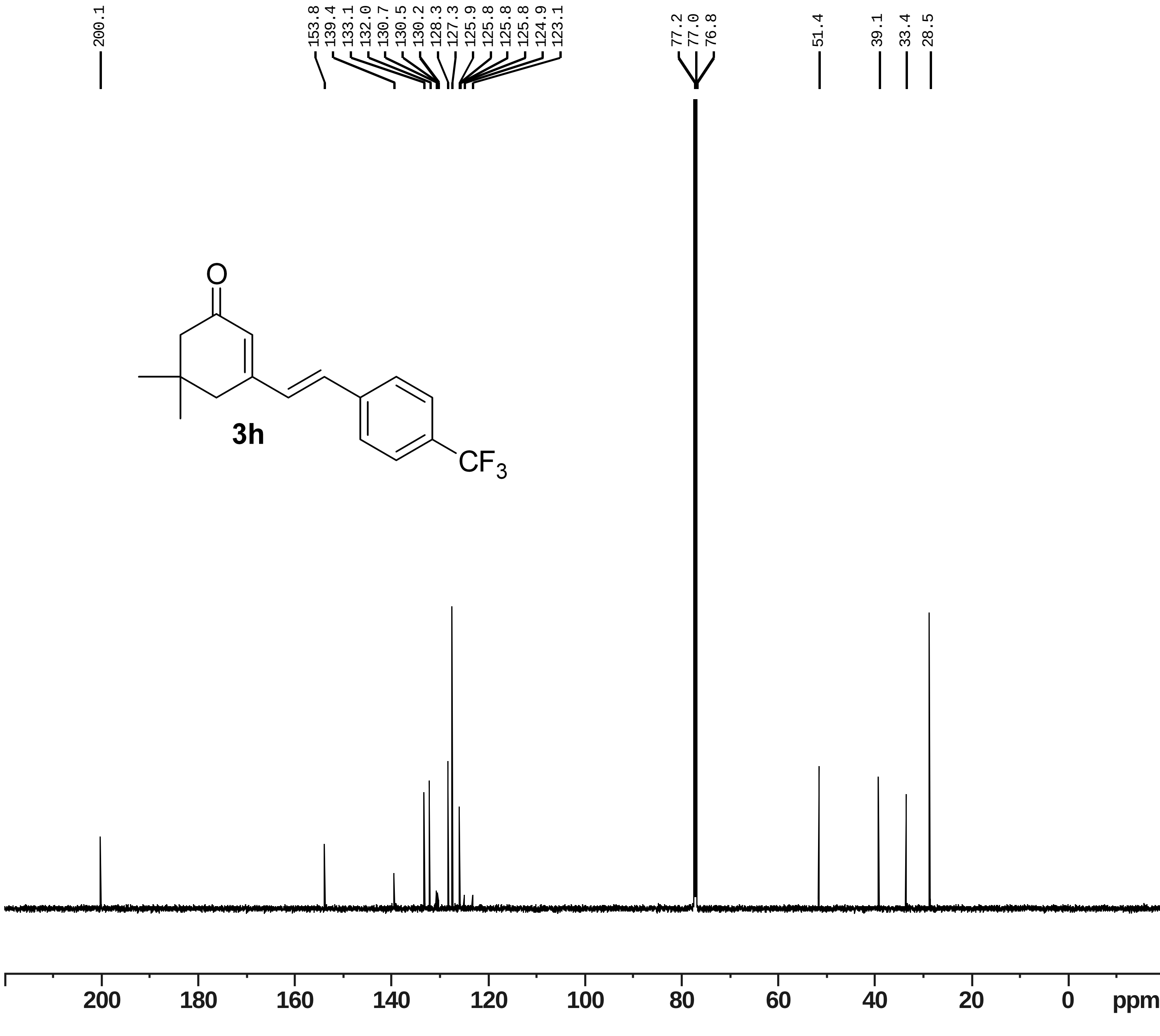
200.1
153.8
139.4
133.1
132.0
130.7
130.5
130.2
128.3
127.3
125.9
125.8
125.8
125.8
124.9
123.1
77.2
77.0
76.8
51.4
39.1
33.4
28.5
O
3h
CF3
200
180
160
140
120
100
80
60
40
20
0
ppm

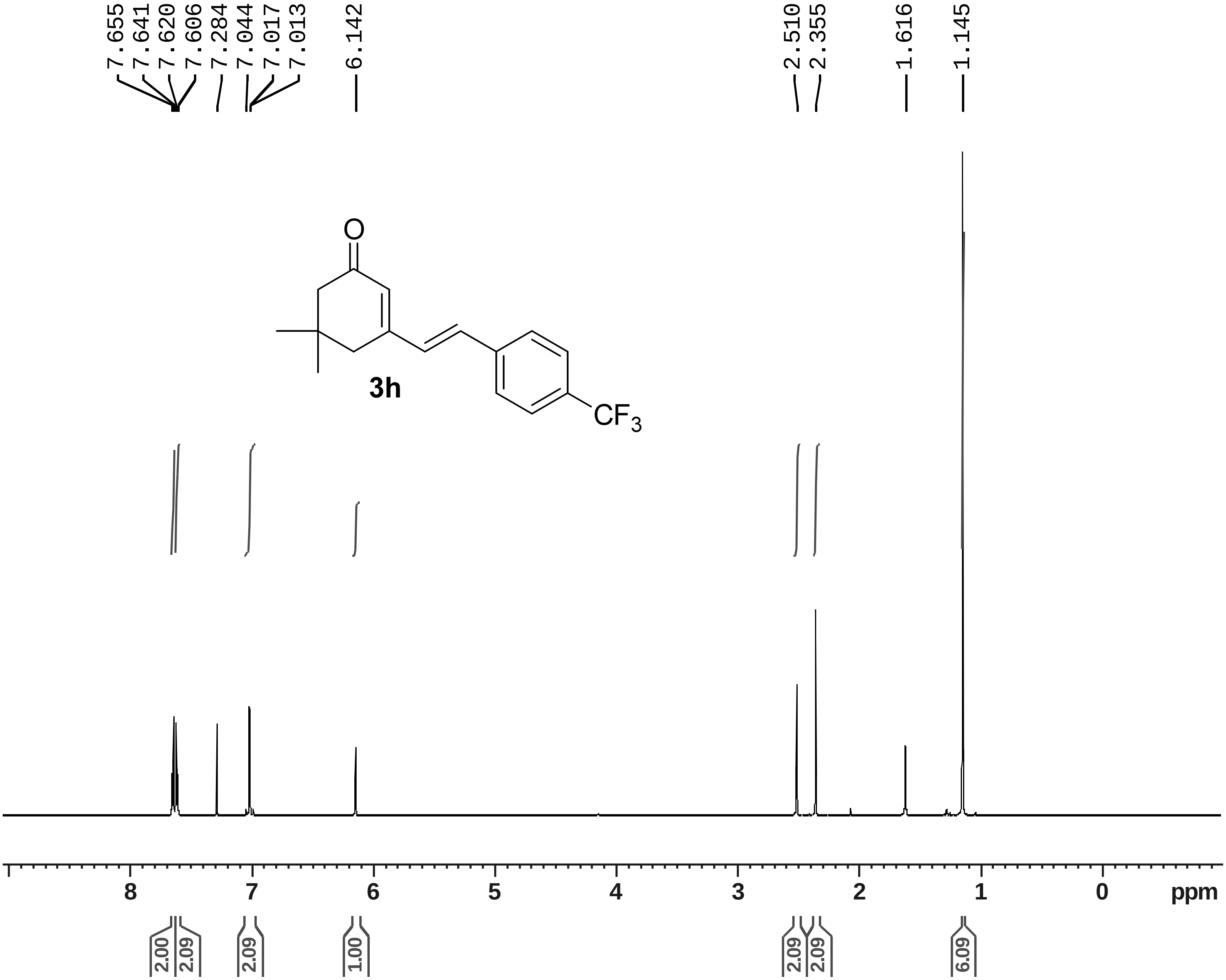

7.655
7.641
7.620
7.606
7.284
7.044
7.017
7.013
6.142
2.510
2.355
1.616
1.145
O
CF3
3h
8
7
6
5
4
3
2
1
0
ppm
2.00
2.09
2.09
1.00
2.09
2.09
6.09

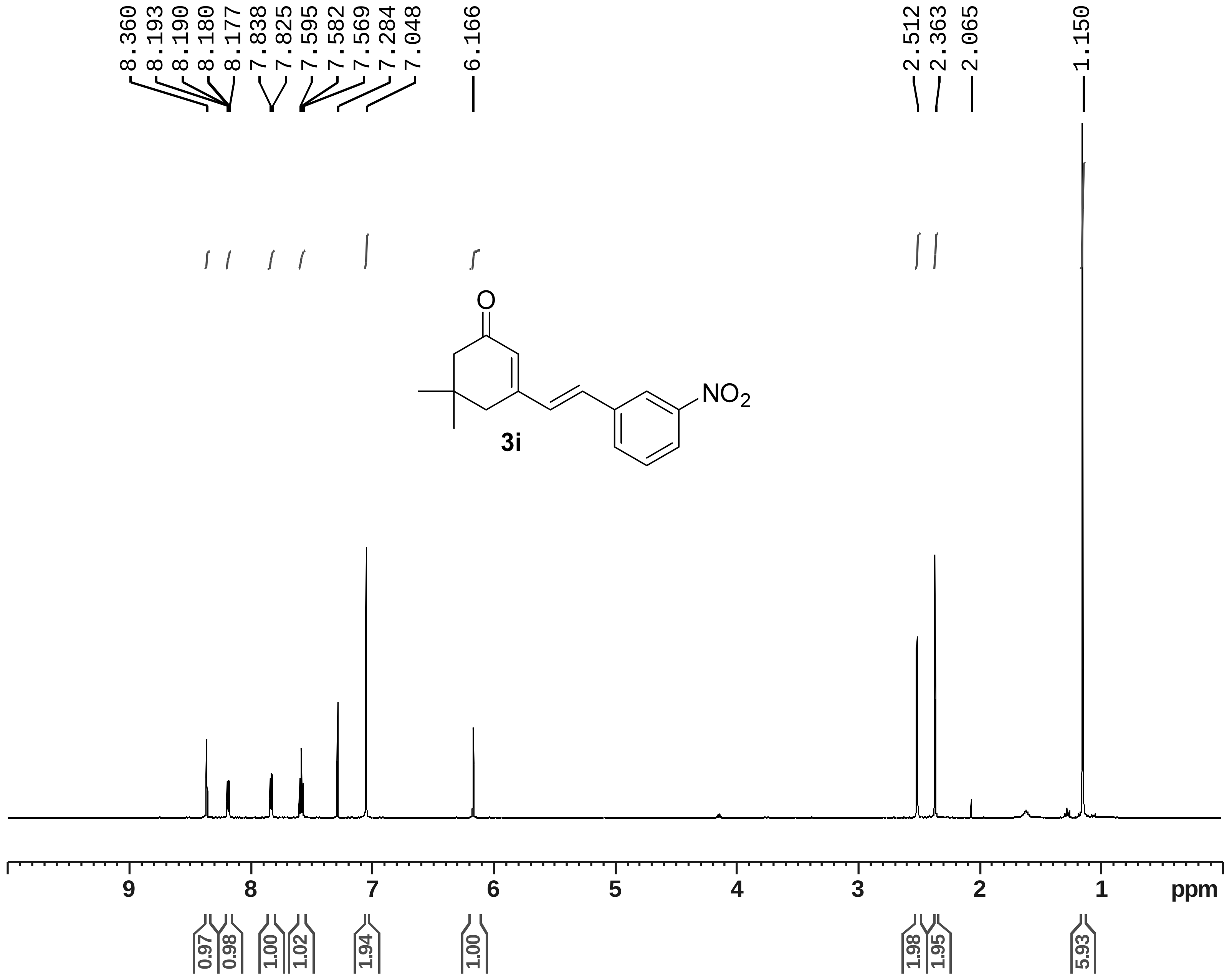
8.360
8.193
8.190
8.180
8.177
7.838
7.825
7.595
7.582
7.569
7.284
7.048
6.166
2.512
2.363
2.065
1.150
O
NO2
3i
9
8
7
6
5
4
3
2
1
ppm
0.97
0.98
1.00
1.02
1.94
1.00
1.98
1.95
5.93

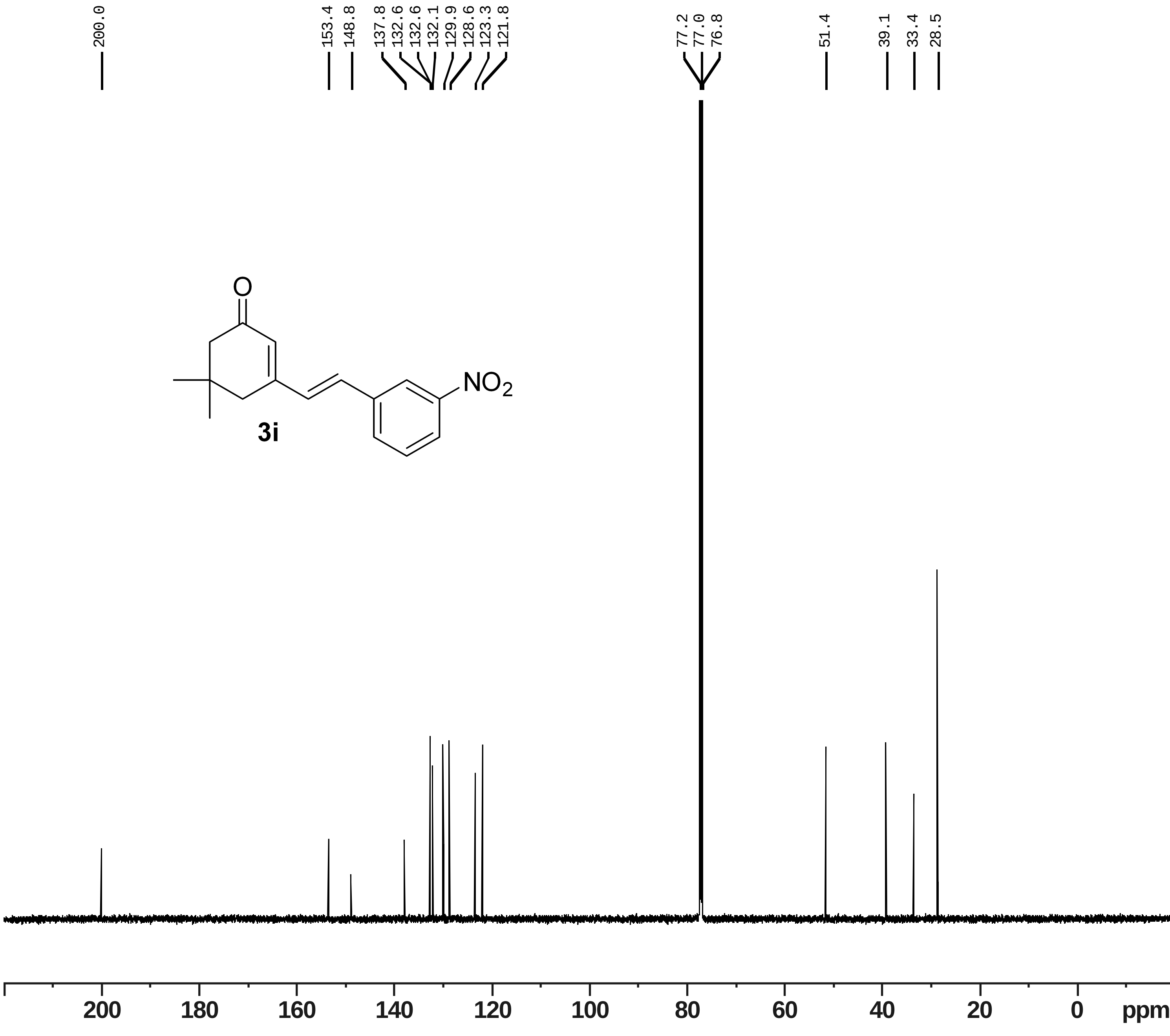
200.0
153.4
148.8
137.8
132.6
132.6
132.1
129.9
128.6
123.3
121.8
77.2
77.0
76.8
51.4
39.1
33.4
28.5
O
NO2
3i
200
180
160
140
120
100
80
60
40
20
0
ppm

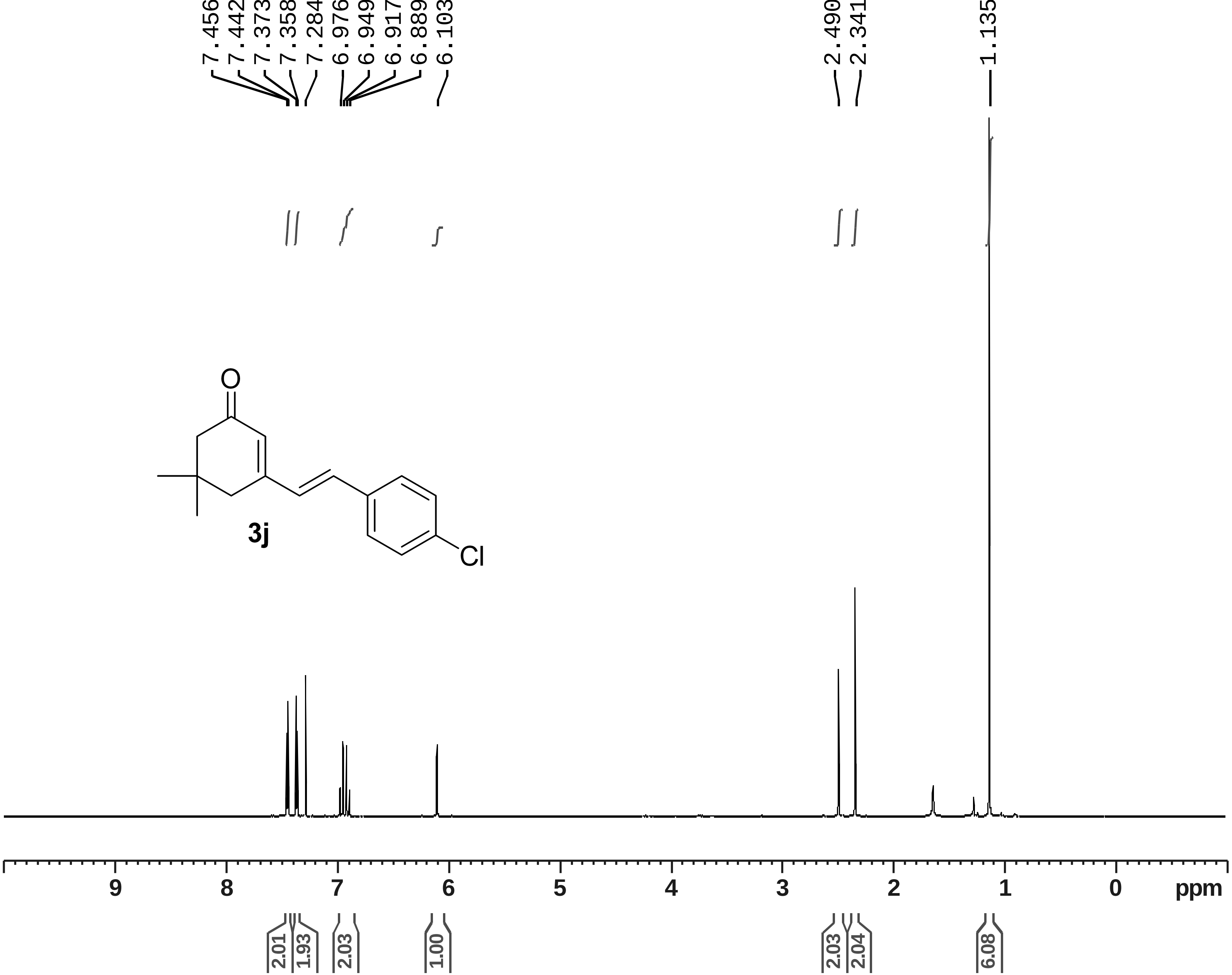

7.456
7.442
7.373
7.358
7.284
6.976
6.949
6.917
6.889
6.103
2.490
2.341
1.135
O
Cl
3j
9
8
7
6
5
4
3
2
1
0
ppm
2.01
1.93
2.03
1.00
2.03
2.04
6.08

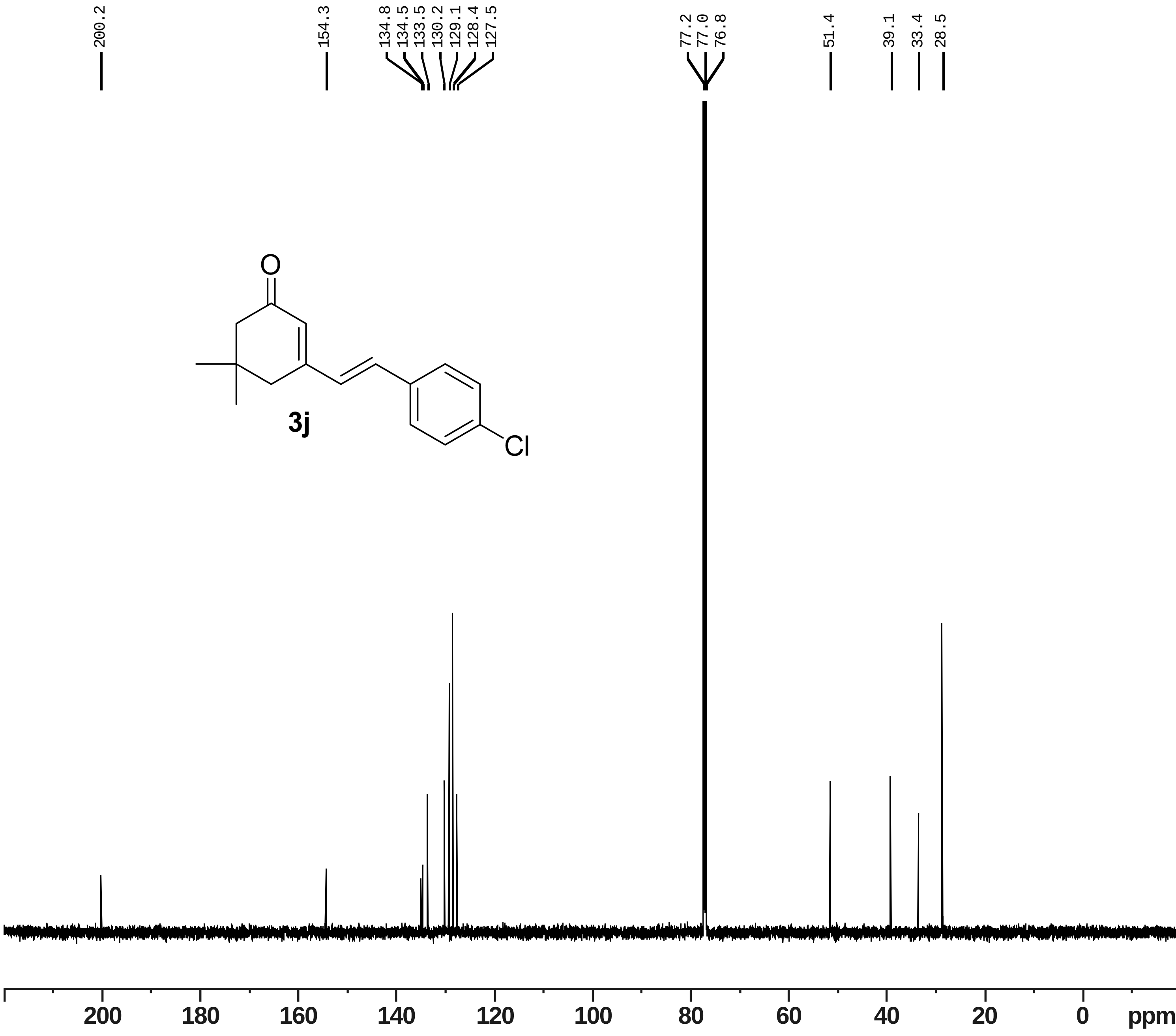
200.2
154.3
134.8
134.5
133.5
130.2
129.1
128.4
127.5
77.2
77.0
76.8
51.4
39.1
33.4
28.5
O
Cl
3j
200
180
160
140
120
100
80
60
40
20
0
ppm

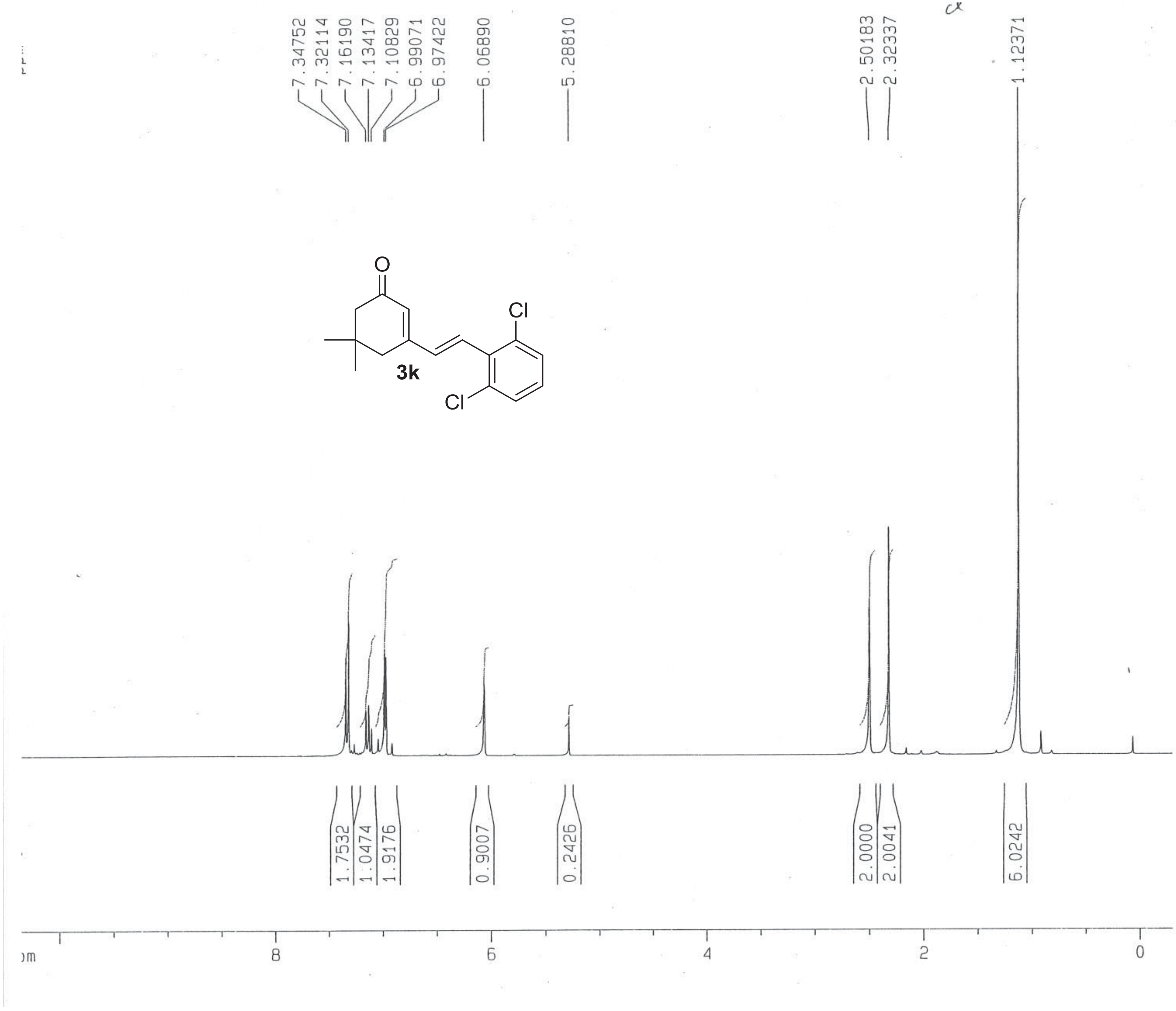

7.34752
7.32114
7.16190
7.13417
7.10829
6.99071
6.97422
6.06890
5.28810
2.50183
2.32337
1.12371
O
Cl
Cl
3k
1.7532
1.0474
1.9176
0.9007
0.2426
2.0000
2.0041
6.0242
8
6
4
2
0

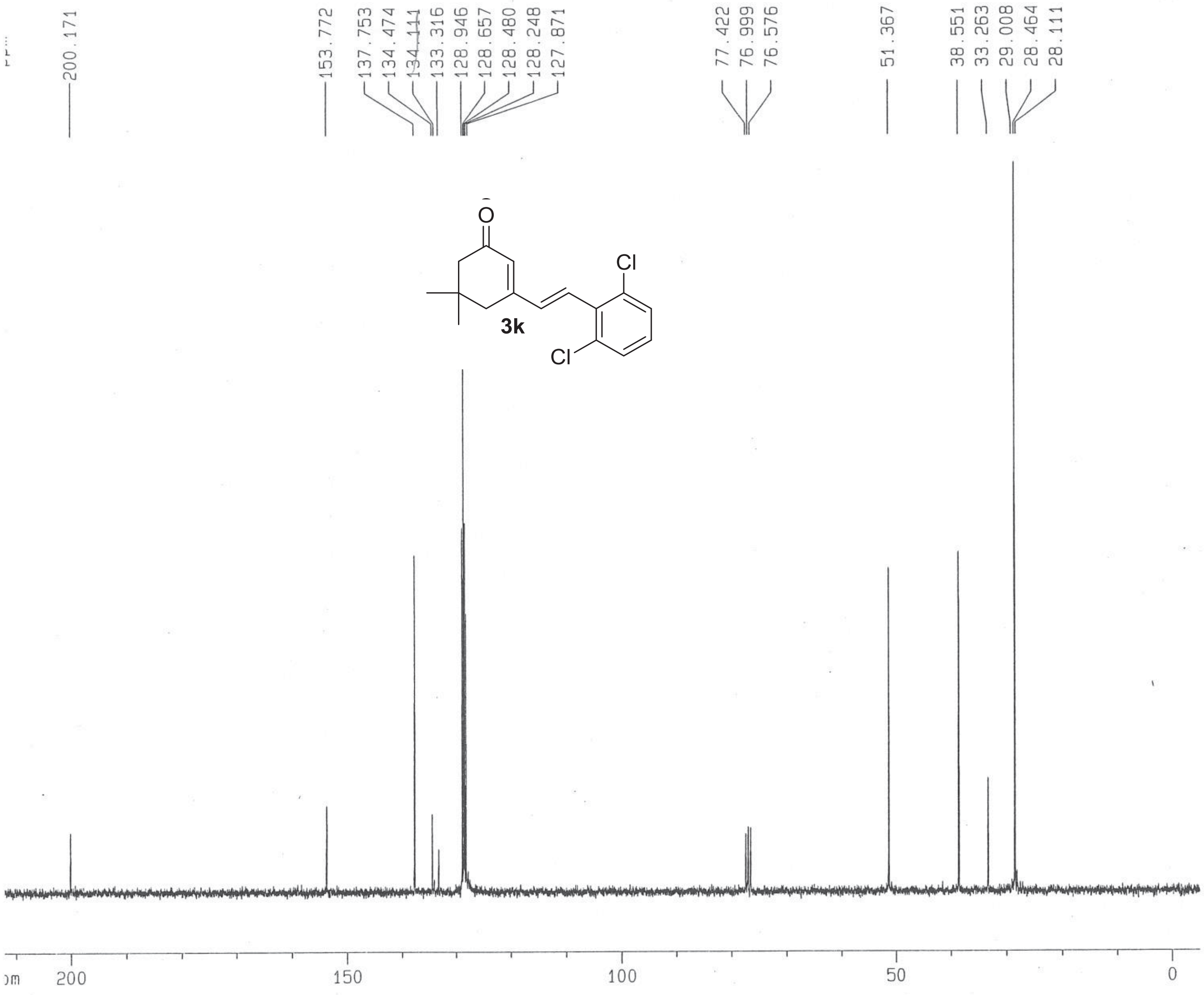
200.171
153.772
137.753
134.474
134.111
133.316
128.946
128.657
128.480
128.248
127.871
77.422
76.999
76.576
51.367
38.551
33.263
29.008
28.464
28.111
O
Cl
Cl
3k
200
150
100
50
0

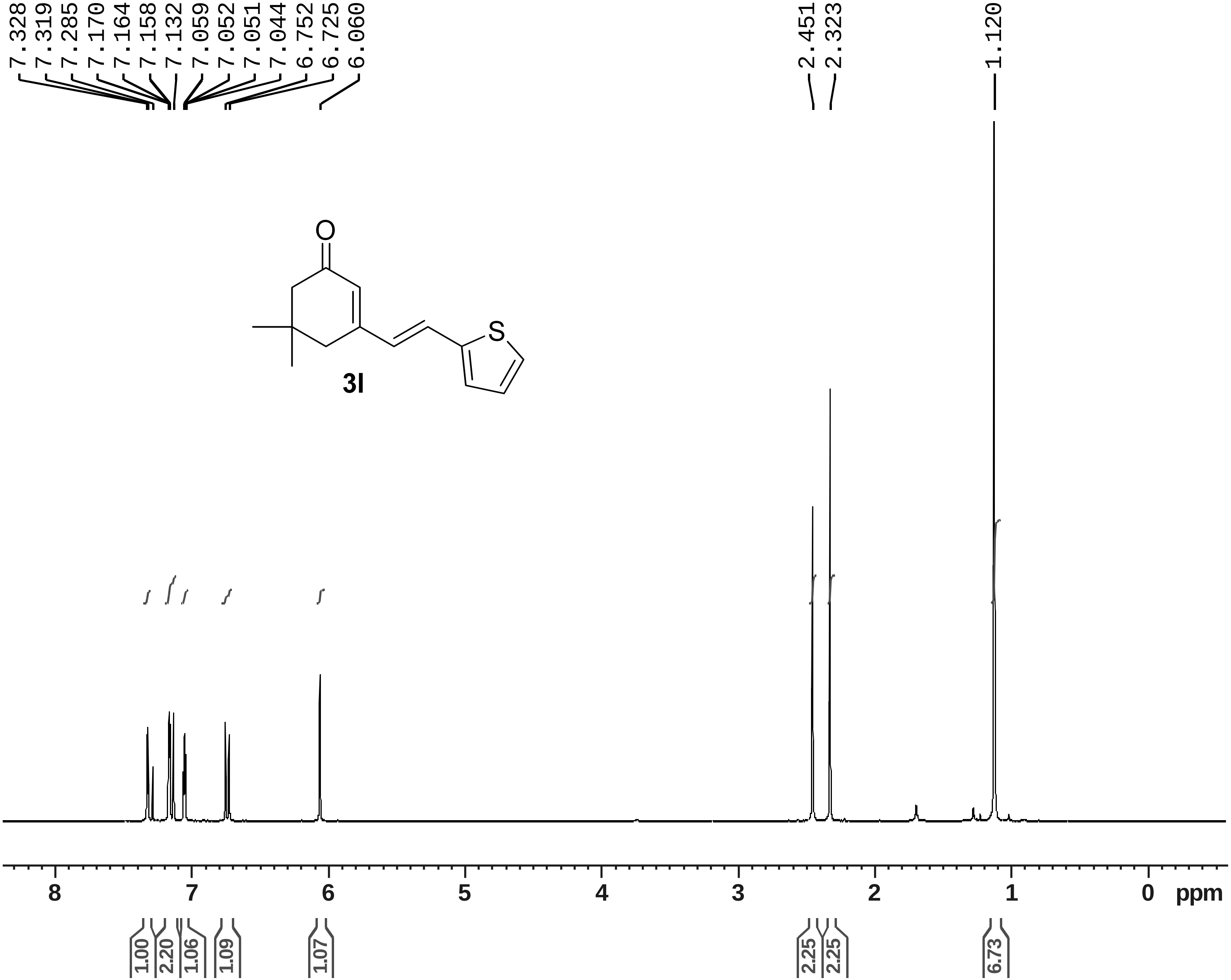
7.328
7.319
7.285
7.170
7.164
7.158
7.132
7.059
7.052
7.051
7.044
6.752
6.725
6.060
2.451
2.323
1.120
O
S
3l
8
7
6
5
4
3
2
1
0
ppm
1.00
2.20
1.06
1.09
1.07
2.25
2.25
6.73

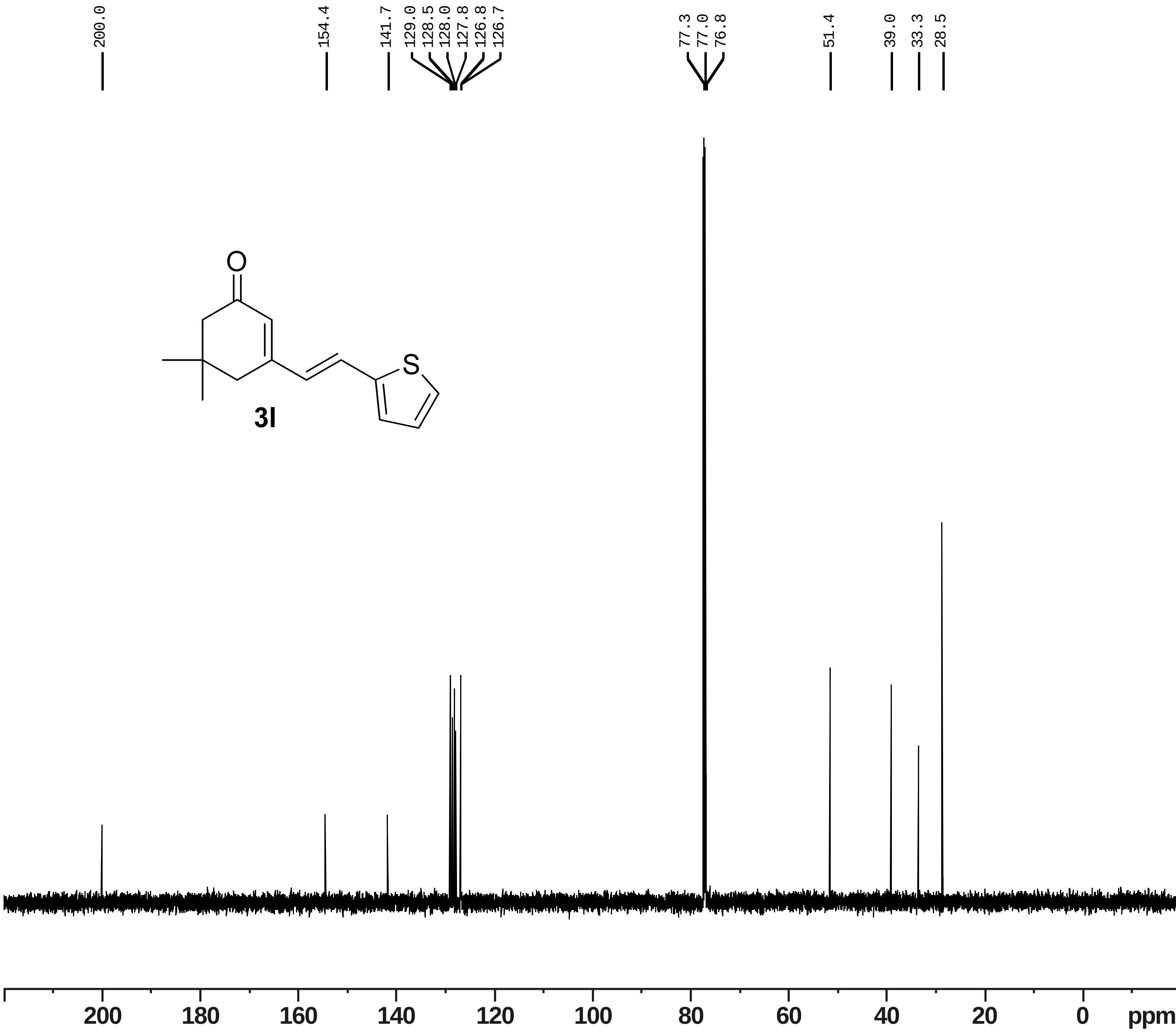
200.0
154.4
141.7
129.0
128.5
128.0
127.8
126.8
126.7
77.3
77.0
76.8
51.4
39.0
33.3
28.5
O
S
3l
200
180
160
140
120
100
80
60
40
20
0
ppm

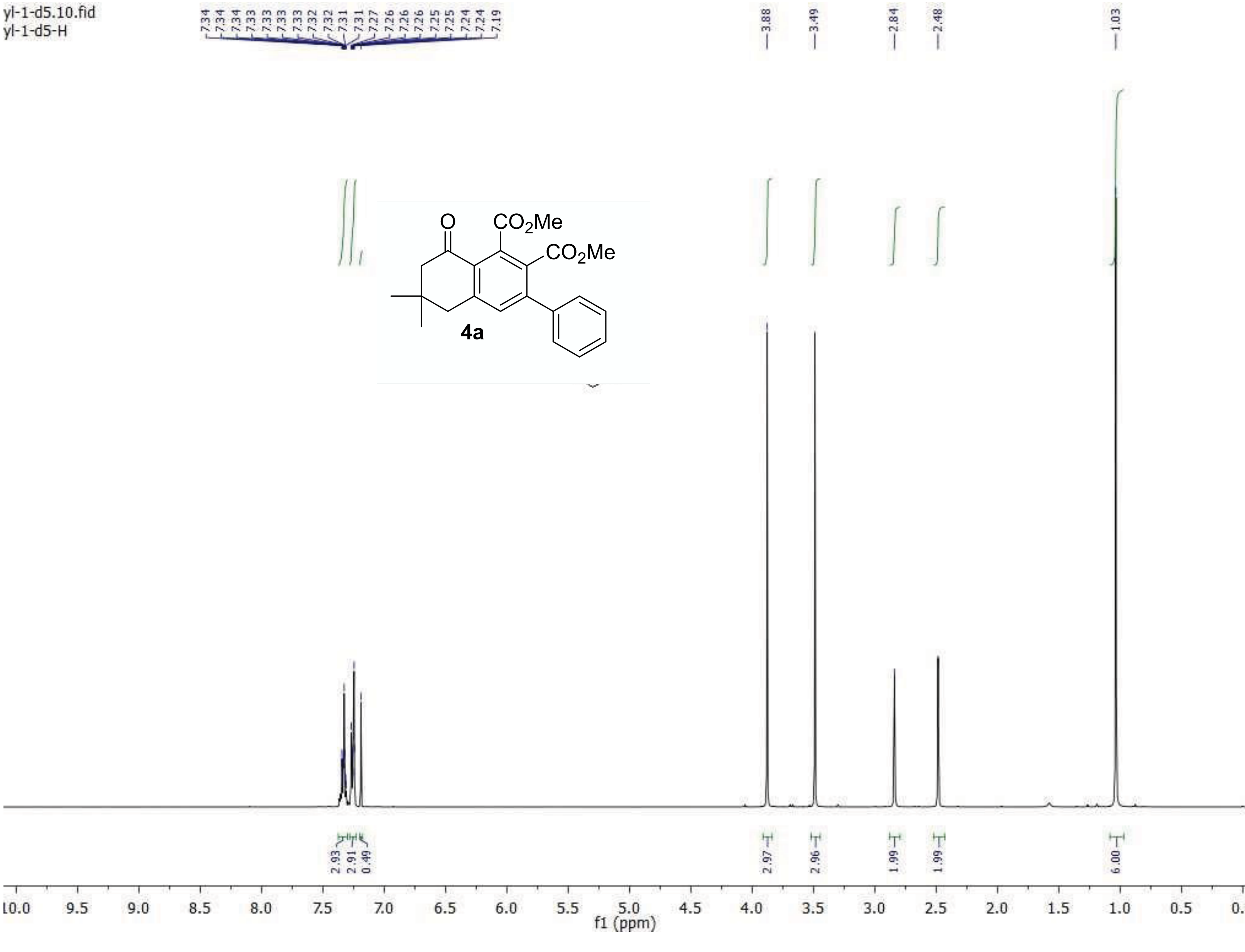

yl-1-d5.10.fid
yl-1-d5-H
7.34 7.34 7.34 7.33 7.33 7.33 7.33 7.32 7.32 7.31 7.31 7.27 7.26 7.26 7.26 7.25 7.25 7.24 7.24 7.19
3.88
3.49
2.84
2.48
1.03
O
CO2Me
CO2Me
4a
2.93
2.91
0.49
2.97
2.96
1.99
1.99
6.00
10.0 9.5 9.0 8.5 8.0 7.5 7.0 6.5 6.0 5.5 5.0 4.5 4.0 3.5 3.0 2.5 2.0 1.5 1.0 0.5
f1 (ppm)

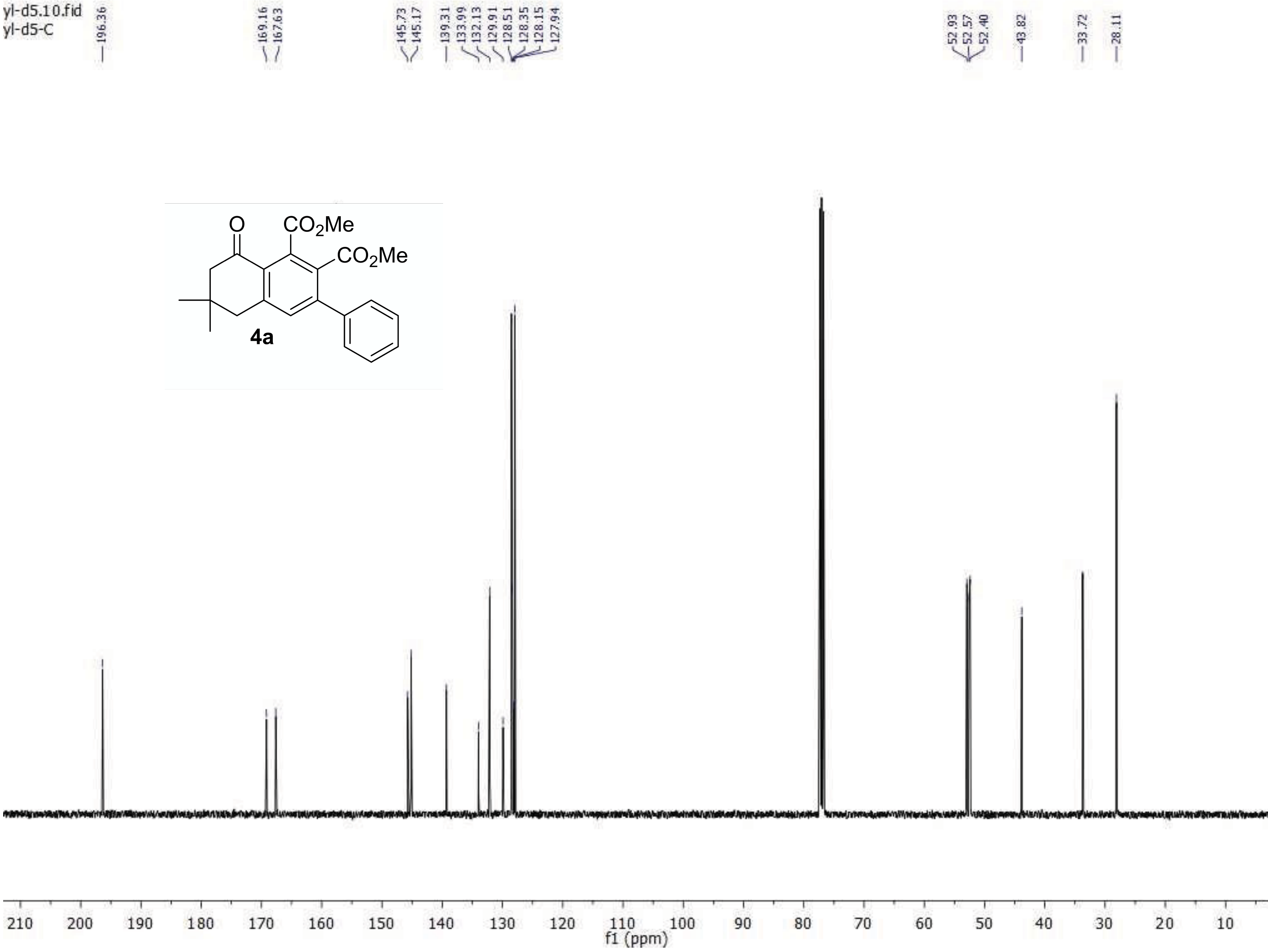

yl-d5.10.fid
yl-d5-C
196.36
169.16
167.63
145.73
145.17
139.31
133.99
132.13
129.91
128.51
128.35
128.15
127.94
52.93
52.57
52.40
43.82
33.72
28.11
O
CO2Me
CO2Me
4a
210 200 190 180 170 160 150 140 130 120 110 100 90 80 70 60 50 40 30 20 10
f1 (ppm)

Spectrum Plot Report

Sample Name	IS-9	Rack Position		Instrument	Instrument 1	Acq Operator	PARTHA
Inj Vol (ul)	10	Plate Position		IRM Status	All ions missed		
Data File	IS-9.d	Acq Method	APCI POS ION MEOH MS.m	Comment	367.1545	Acq Time (Local)	29-Apr-25 2:44:09 PM (UTC+04:00)

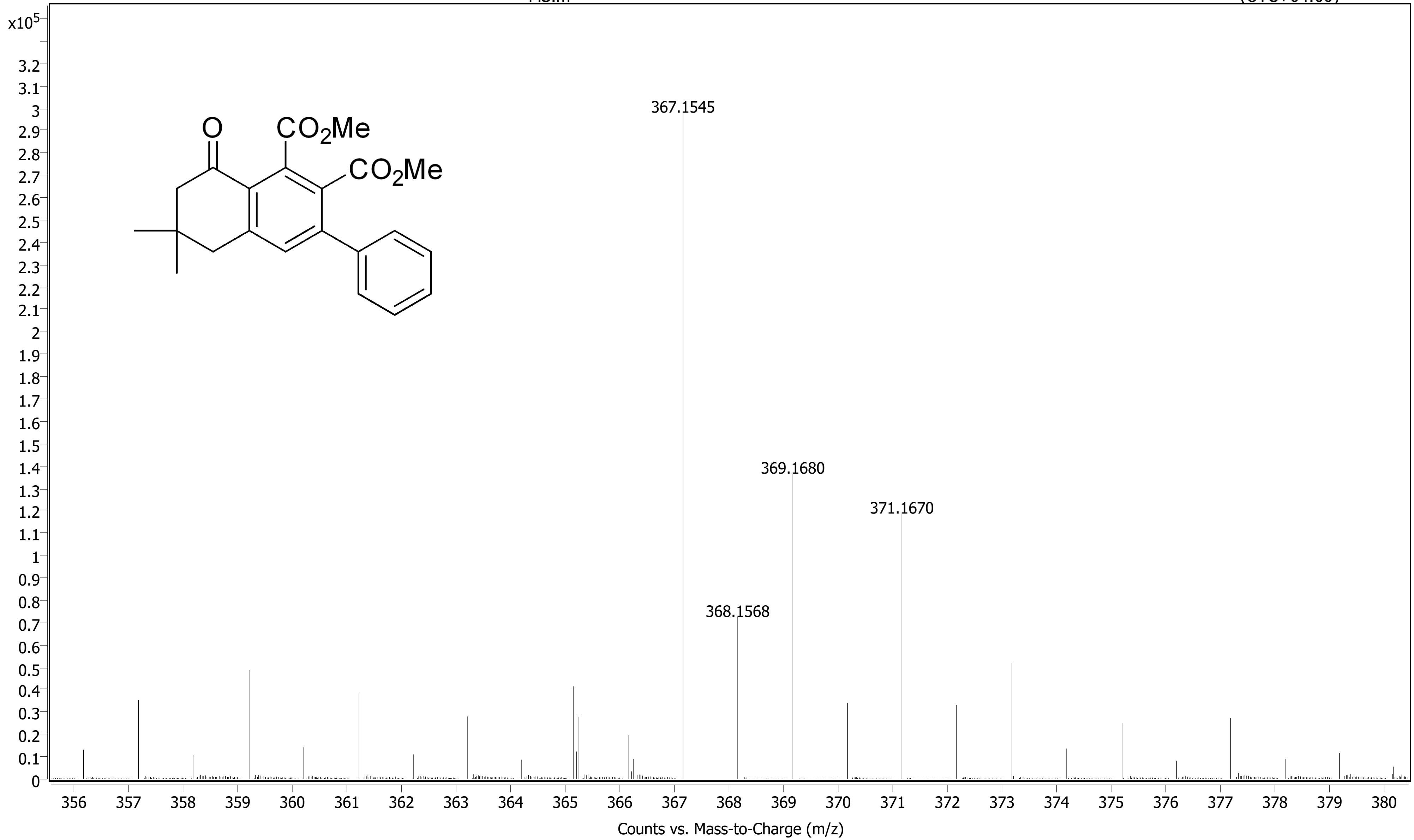

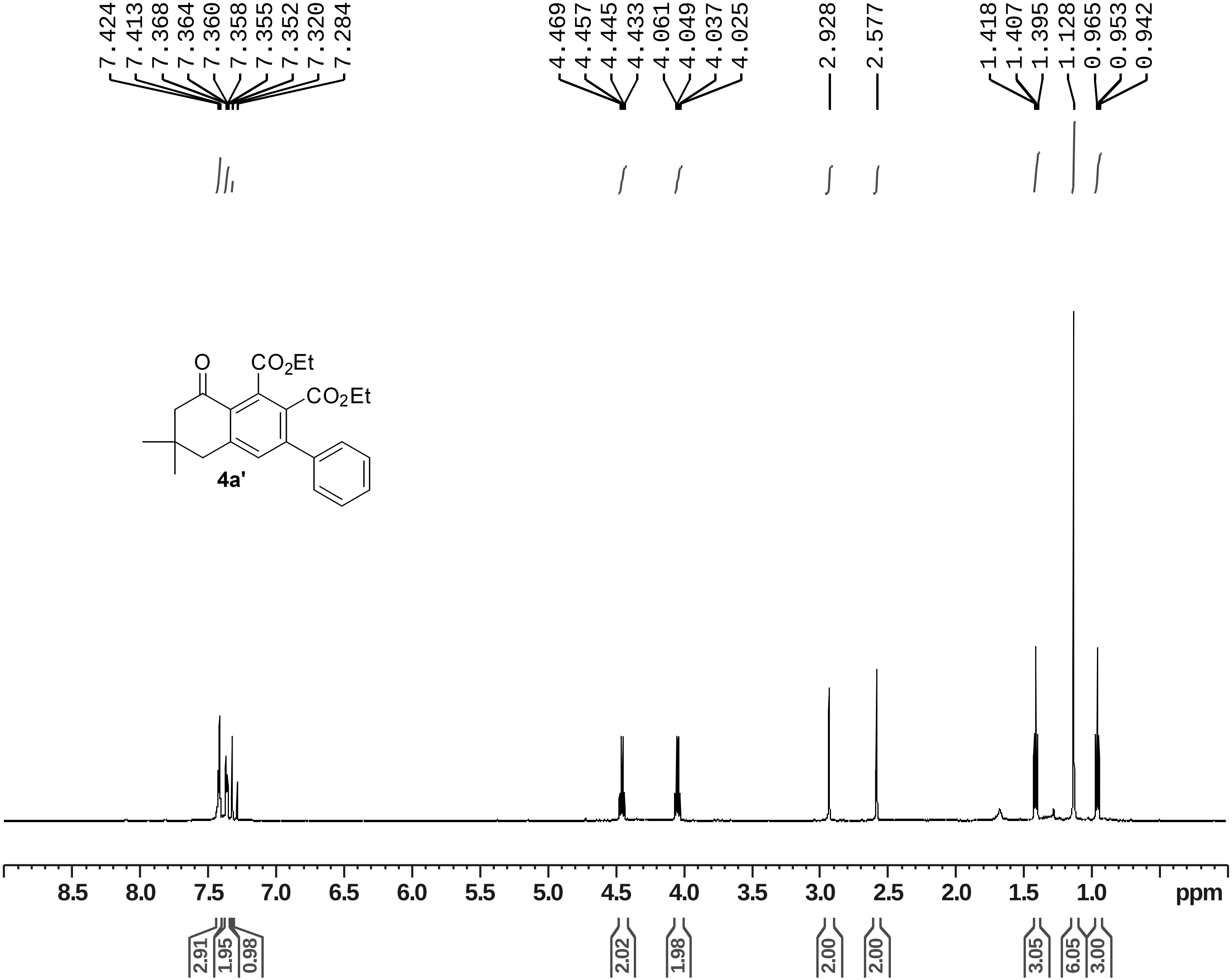
7.424
7.413
7.368
7.364
7.360
7.358
7.355
7.352
7.320
7.284
4.469
4.457
4.445
4.433
4.061
4.049
4.037
4.025
2.928
2.577
1.418
1.407
1.395
1.128
0.965
0.953
0.942
O
CO2Et
CO2Et
4a'
8.5
8.0
7.5
7.0
6.5
6.0
5.5
5.0
4.5
4.0
3.5
3.0
2.5
2.0
1.5
1.0
ppm
2.91
1.95
0.98
2.02
1.98
2.00
2.00
3.05
6.05
3.00

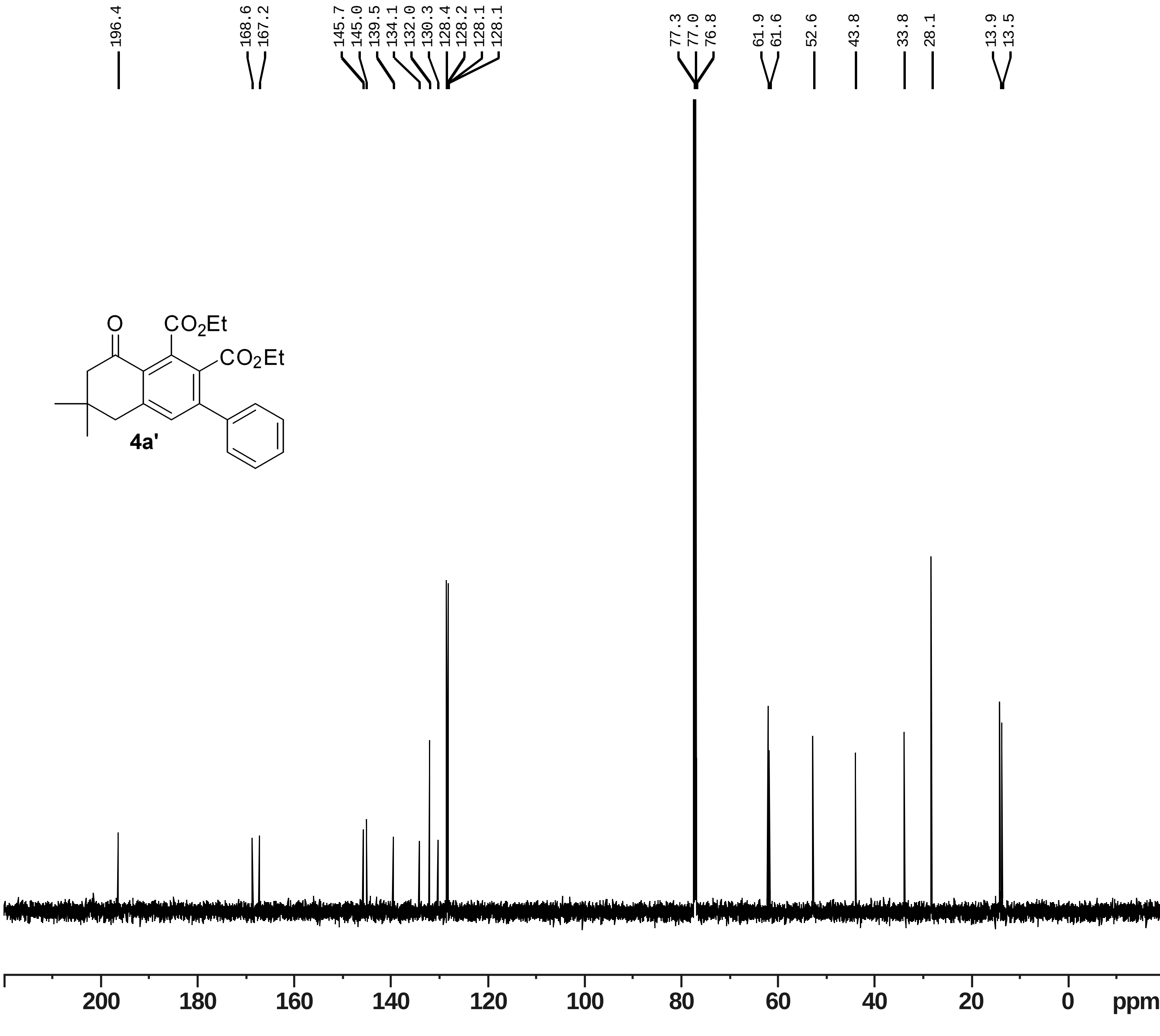
196.4
168.6
167.2
145.7
145.0
139.5
134.1
132.0
130.3
128.4
128.2
128.1
128.1
77.3
77.0
76.8
61.9
61.6
52.6
43.8
33.8
28.1
13.9
13.5
O
CO2Et
CO2Et
4a'
200
180
160
140
120
100
80
60
40
20
0
ppm

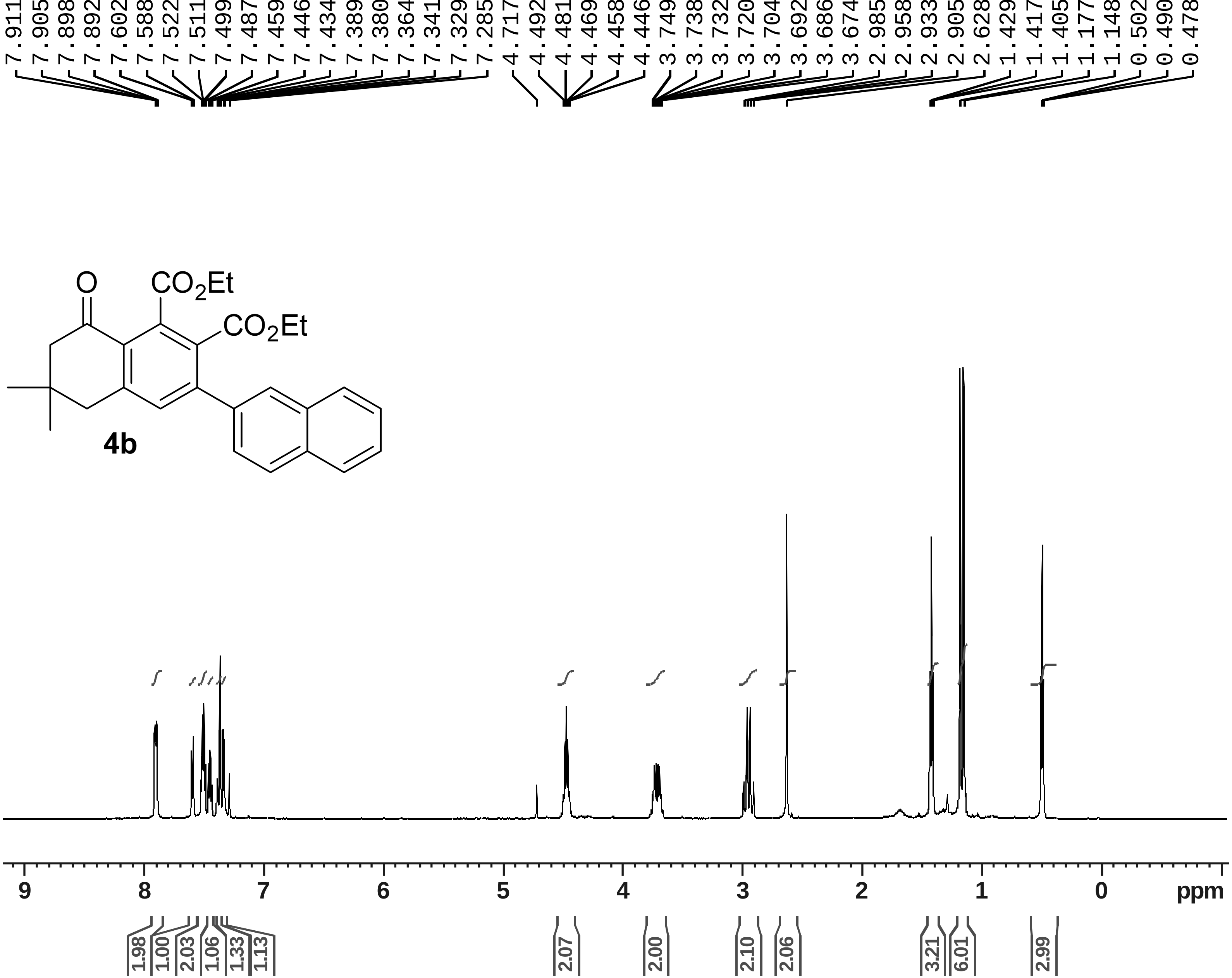
7.911
7.905
7.898
7.892
7.602
7.588
7.522
7.511
7.499
7.487
7.459
7.446
7.434
7.389
7.380
7.364
7.341
7.329
7.285
4.717
4.492
4.481
4.469
4.458
4.446
3.749
3.738
3.732
3.720
3.704
3.692
3.686
3.674
2.985
2.958
2.933
2.905
2.628
1.429
1.417
1.405
1.177
1.148
0.502
0.490
0.478
O
CO2Et
CO2Et
4b
9
8
7
6
5
4
3
2
1
0
ppm
1.98
1.00
2.03
1.06
1.33
1.13
2.07
2.00
2.10
2.06
3.21
6.01
2.99

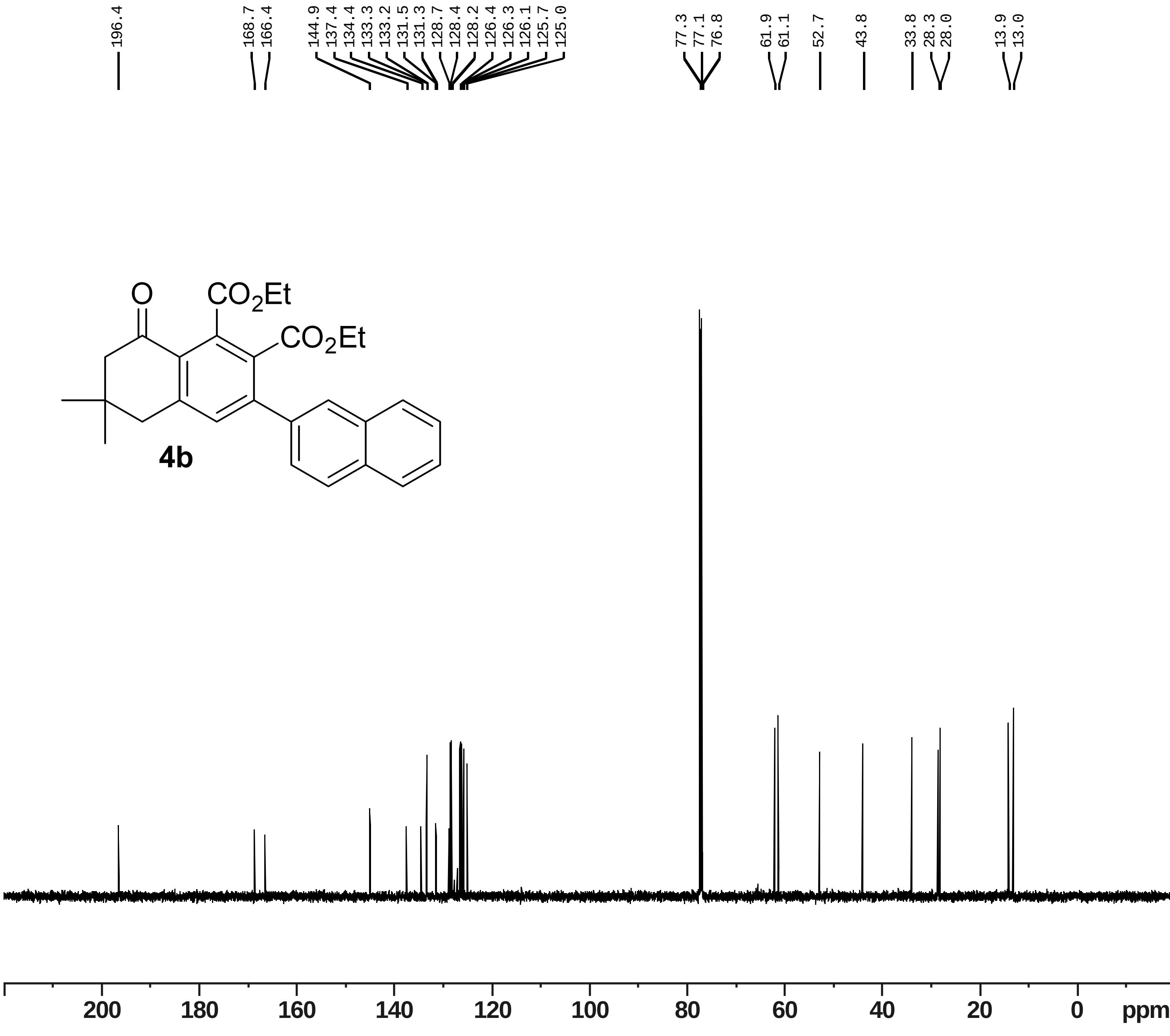
196.4
168.7
166.4
144.9
137.4
134.4
133.3
133.2
131.5
131.3
128.7
128.4
128.2
126.4
126.3
126.1
125.7
125.0
77.3
77.1
76.8
61.9
61.1
52.7
43.8
33.8
28.3
28.0
13.9
13.0
O
CO2Et
CO2Et
4b
200
180
160
140
120
100
80
60
40
20
0
ppm

Spectrum Plot Report

Agilent

Sample Name	IS-21	Rack Position		Instrument	Instrument 1	Acq Operator	PARTHA
Inj Vol (ul)	10	Plate Position		IRM Status	Success		
Data File	IS-21.d	Acq Method	APCI POS ION CDCL3 MS.m	Comment	445.2010	Acq Time (Local)	02-Jun-25 10:57:18 AM (UTC+04:00)

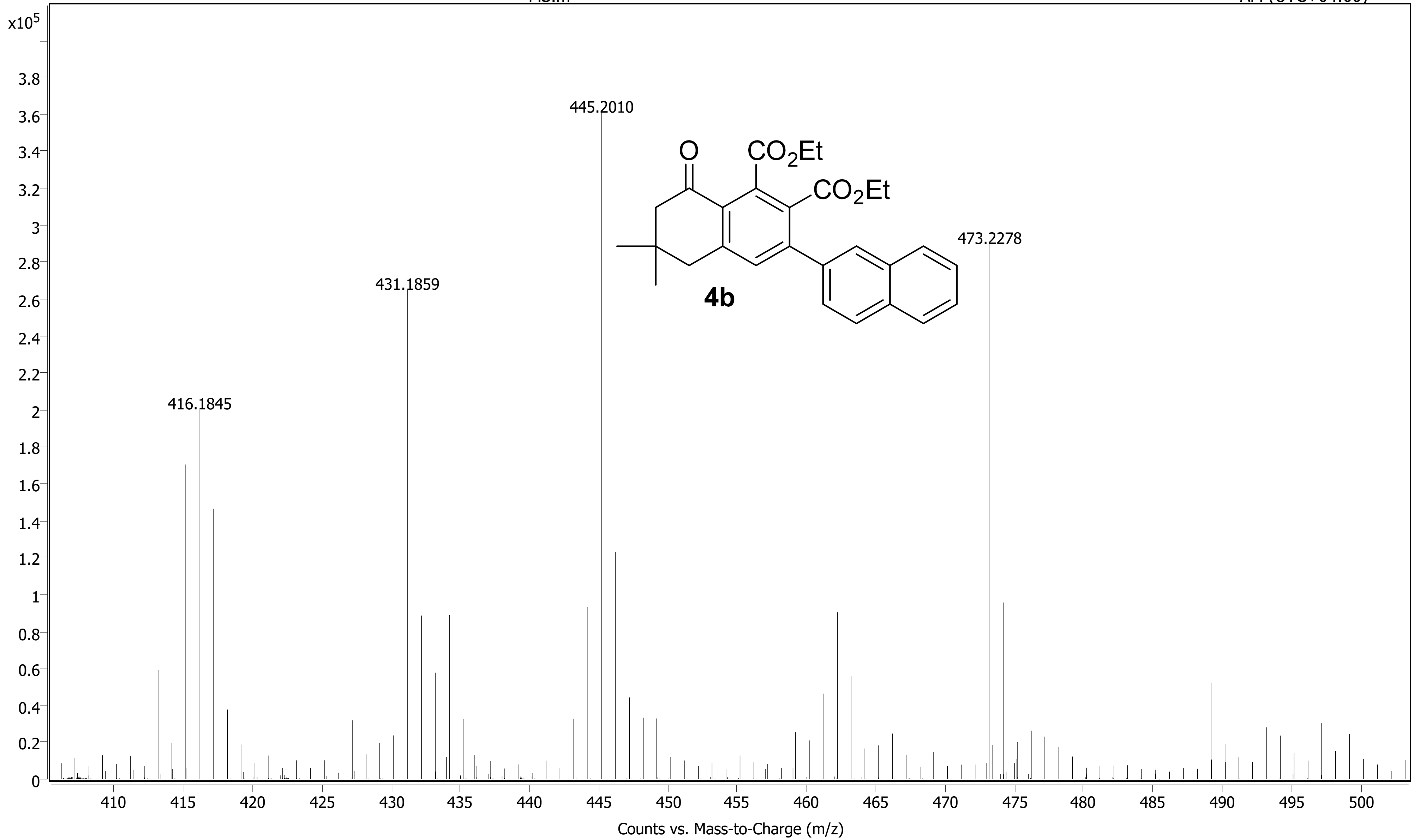

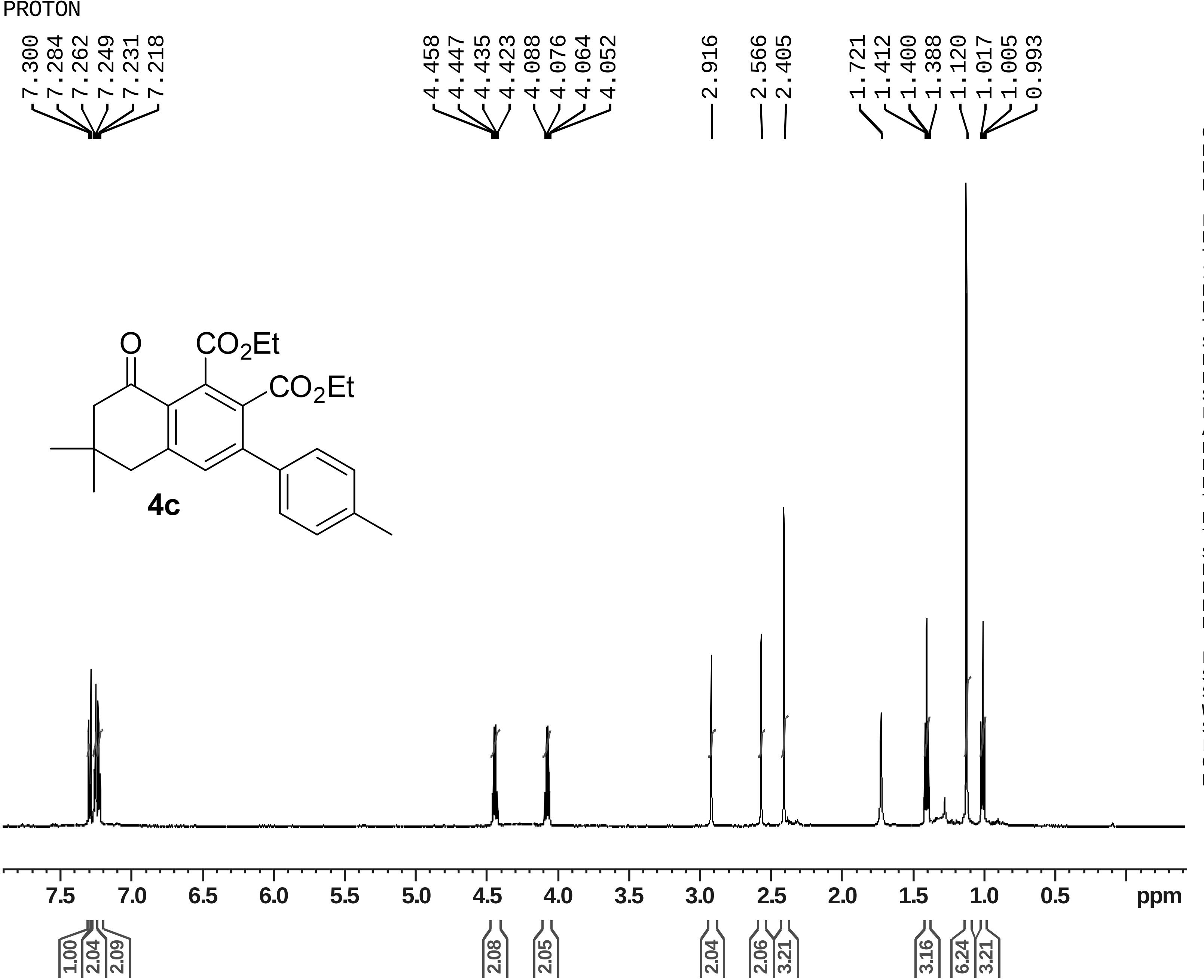

PROTON
7.300
7.284
7.262
7.249
7.231
7.218
4.458
4.447
4.435
4.423
4.088
4.076
4.064
4.052
2.916
2.566
2.405
1.721
1.412
1.400
1.388
1.120
1.017
1.005
0.993
O
CO2Et
CO2Et
4c
7.5
7.0
6.5
6.0
5.5
5.0
4.5
4.0
3.5
3.0
2.5
2.0
1.5
1.0
0.5
ppm
1.00
2.04
2.09
2.08
2.05
2.04
2.06
3.21
3.16
6.24
3.21

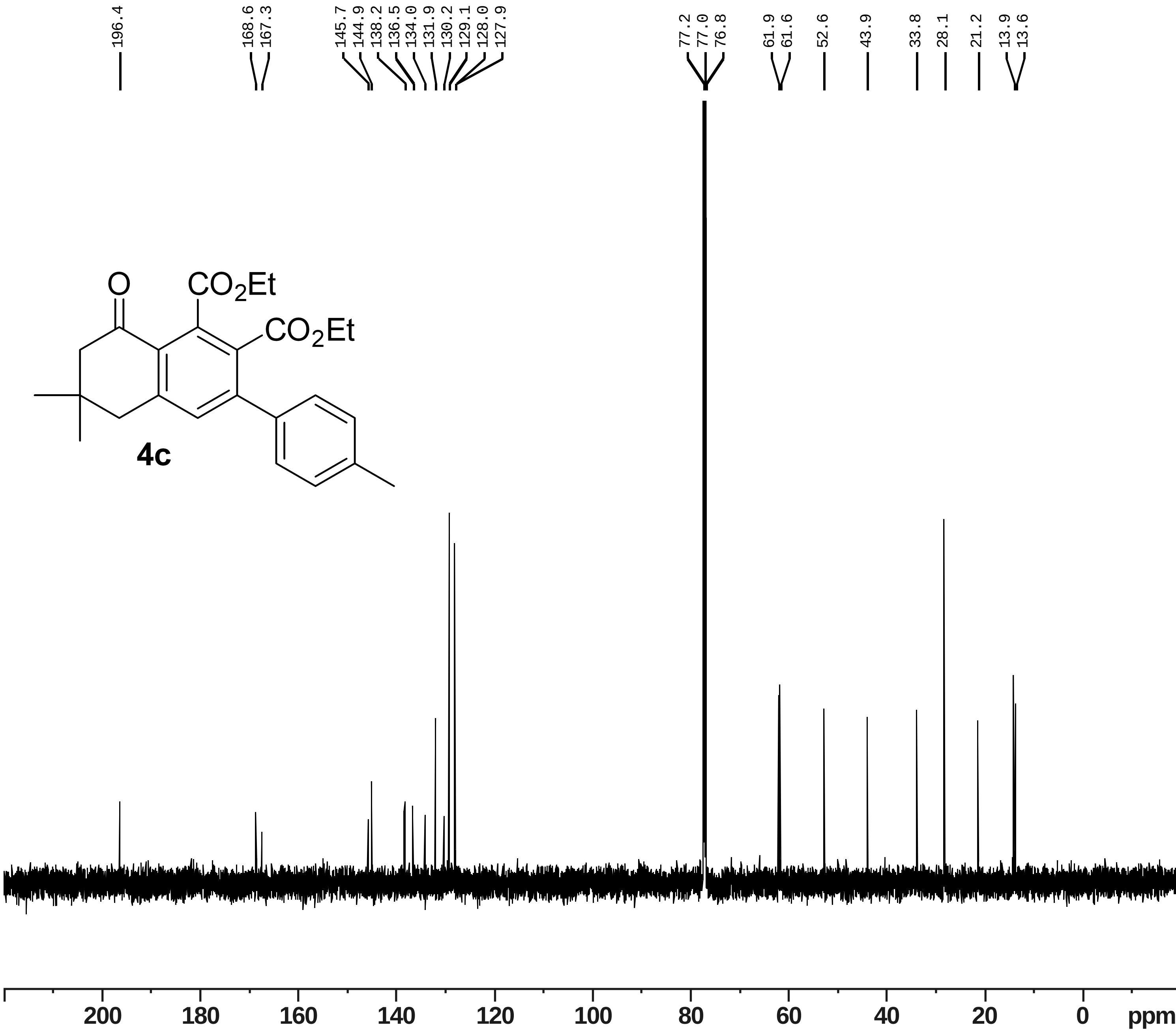
196.4
168.6
167.3
145.7
144.9
138.2
136.5
134.0
131.9
130.2
129.1
128.0
127.9
77.2
77.0
76.8
61.9
61.6
52.6
43.9
33.8
28.1
21.2
13.9
13.6
O
CO2Et
CO2Et
4c
200
180
160
140
120
100
80
60
40
20
0
ppm

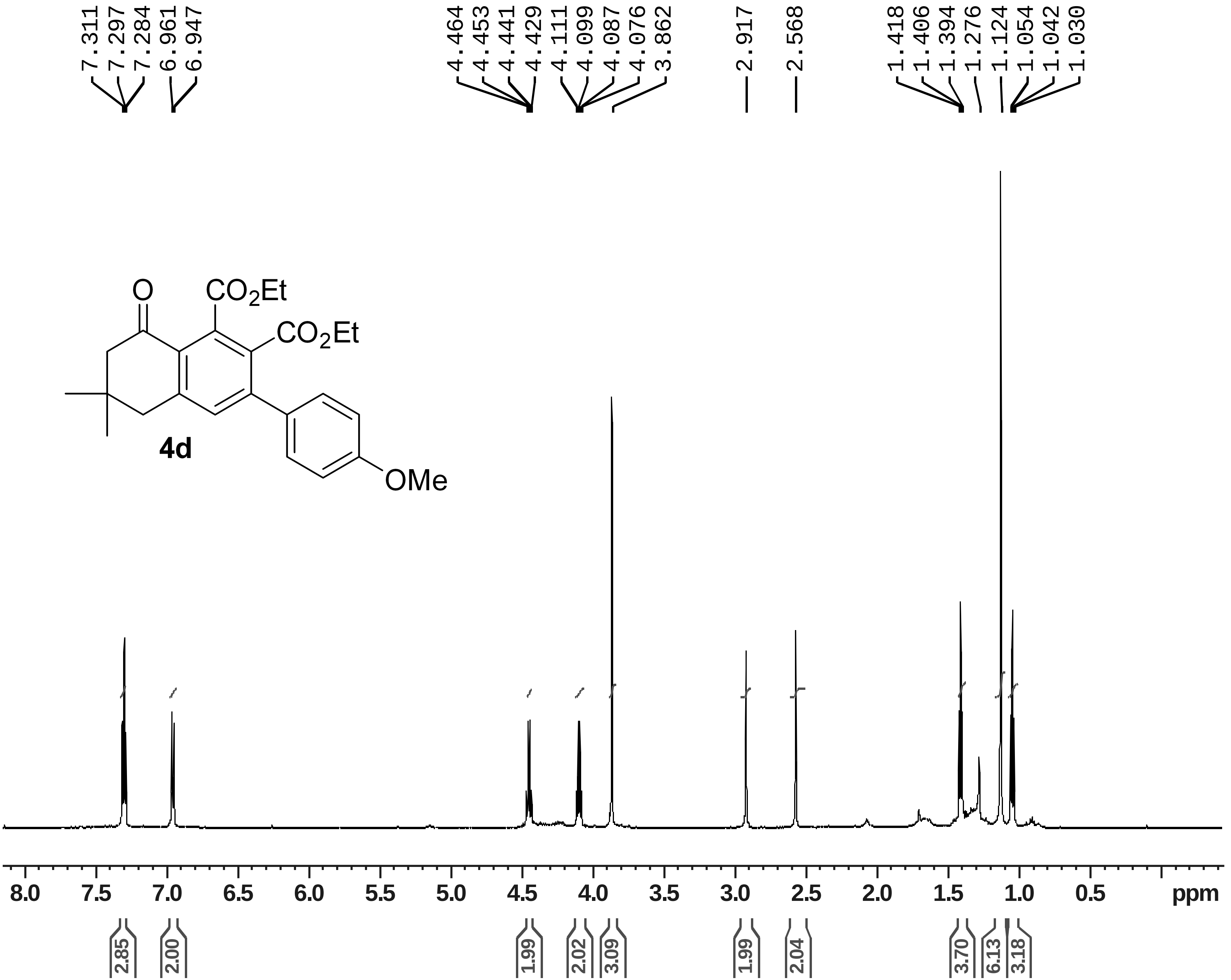
7.311
7.297
7.284
6.961
6.947
4.464
4.453
4.441
4.429
4.111
4.099
4.087
4.076
3.862
2.917
2.568
1.418
1.406
1.394
1.276
1.124
1.054
1.042
1.030
O
CO2Et
CO2Et
OMe
4d
8.0
7.5
7.0
6.5
6.0
5.5
5.0
4.5
4.0
3.5
3.0
2.5
2.0
1.5
1.0
0.5
ppm
2.85
2.00
1.99
2.02
3.09
1.99
2.04
3.70
6.13
3.18

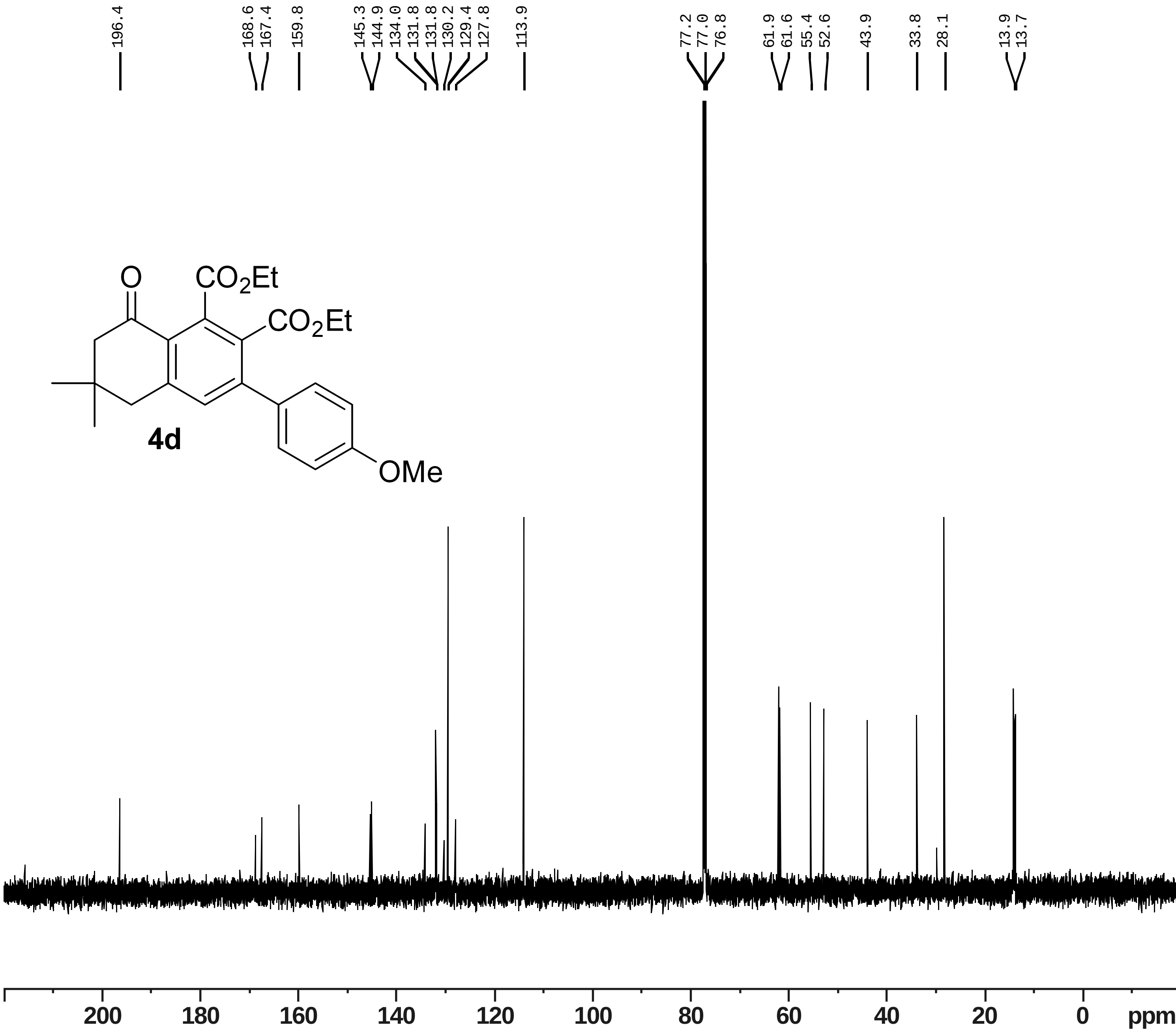
196.4
168.6
167.4
159.8
145.3
144.9
134.0
131.8
131.8
130.2
129.4
127.8
113.9
77.2
77.0
76.8
61.9
61.6
55.4
52.6
43.9
33.8
28.1
13.9
13.7
O
CO2Et
CO2Et
OMe
4d
200
180
160
140
120
100
80
60
40
20
0
ppm

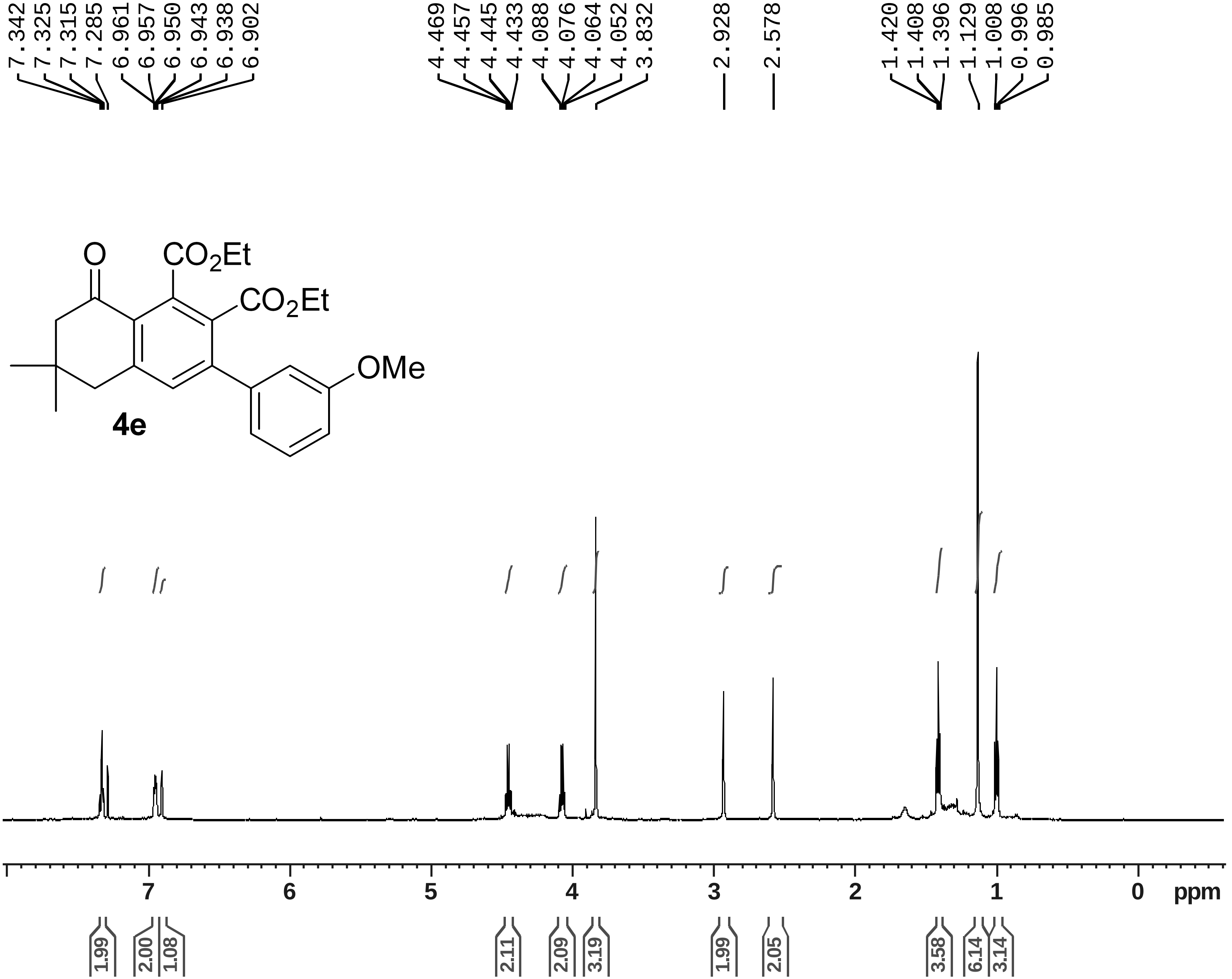
7.342
7.325
7.315
7.285
6.961
6.957
6.950
6.943
6.938
6.902
4.469
4.457
4.445
4.433
4.088
4.076
4.064
4.052
3.832
2.928
2.578
1.420
1.408
1.396
1.129
1.008
0.996
0.985
O
CO2Et
CO2Et
OMe
4e
7
6
5
4
3
2
1
0
ppm
1.99
2.00
1.08
2.11
2.09
3.19
1.99
2.05
3.58
6.14
3.14

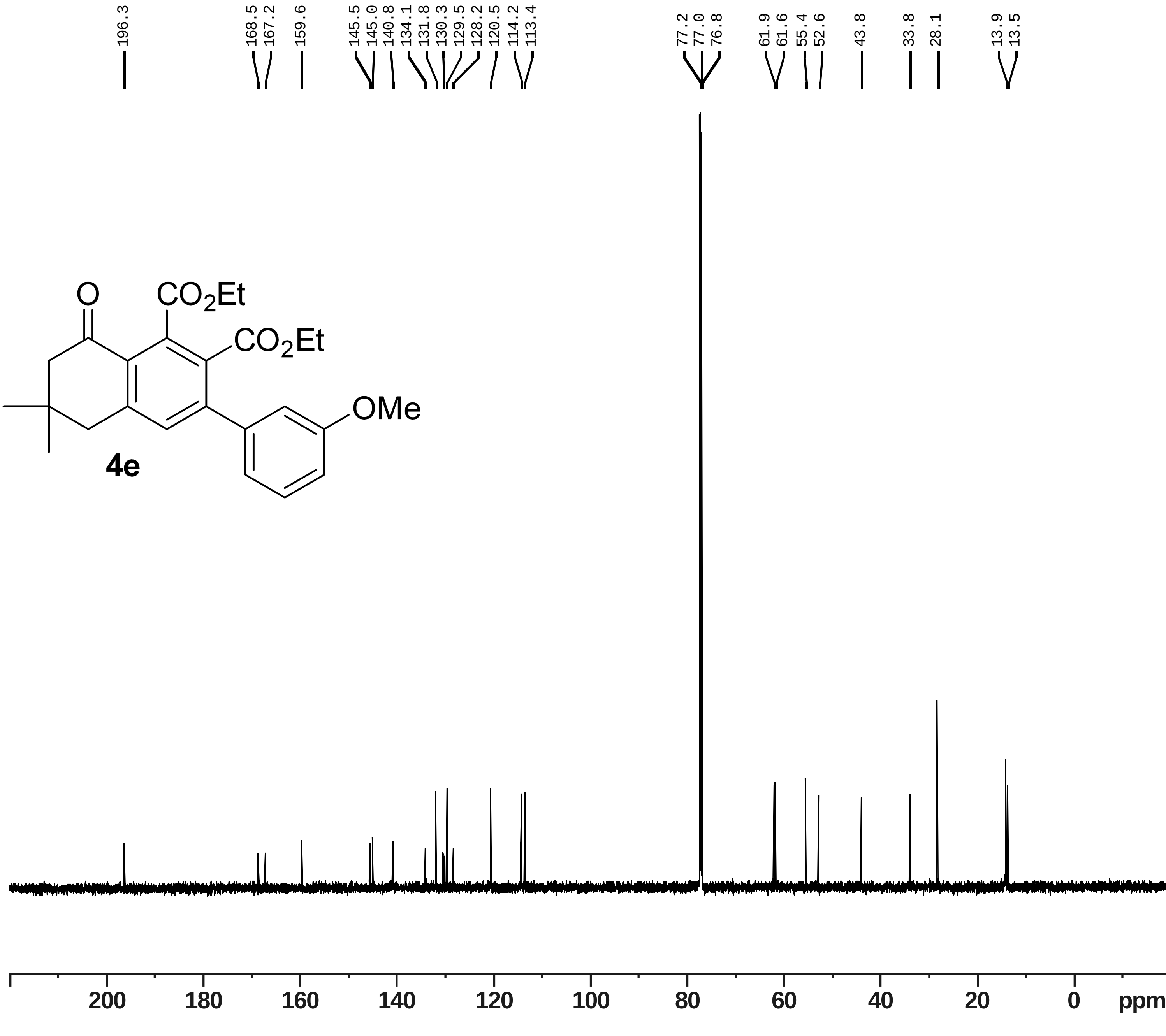
196.3
168.5
167.2
159.6
145.5
145.0
140.8
134.1
131.8
130.3
129.5
128.2
120.5
114.2
113.4
77.2
77.0
76.8
61.9
61.6
55.4
52.6
43.8
33.8
28.1
13.9
13.5
O
CO2Et
CO2Et
OMe
4e
200
180
160
140
120
100
80
60
40
20
0
ppm

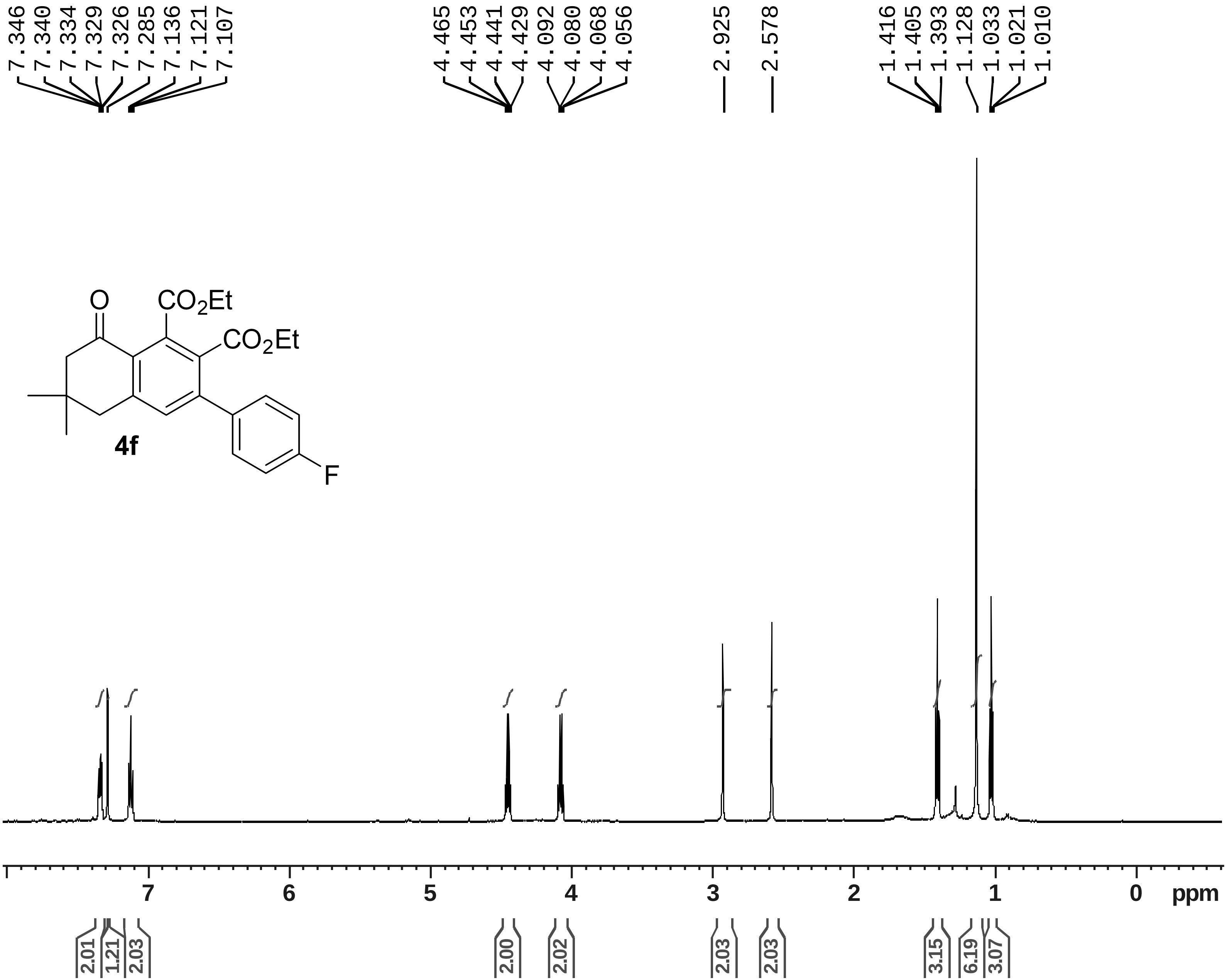
7.346
7.340
7.334
7.329
7.326
7.285
7.136
7.121
7.107
4.465
4.453
4.441
4.429
4.092
4.080
4.068
4.056
2.925
2.578
1.416
1.405
1.393
1.128
1.033
1.021
1.010
O
CO2Et
CO2Et
F
4f
7
6
5
4
3
2
1
0
ppm
2.01
1.21
2.03
2.00
2.02
2.03
2.03
3.15
6.19
3.07

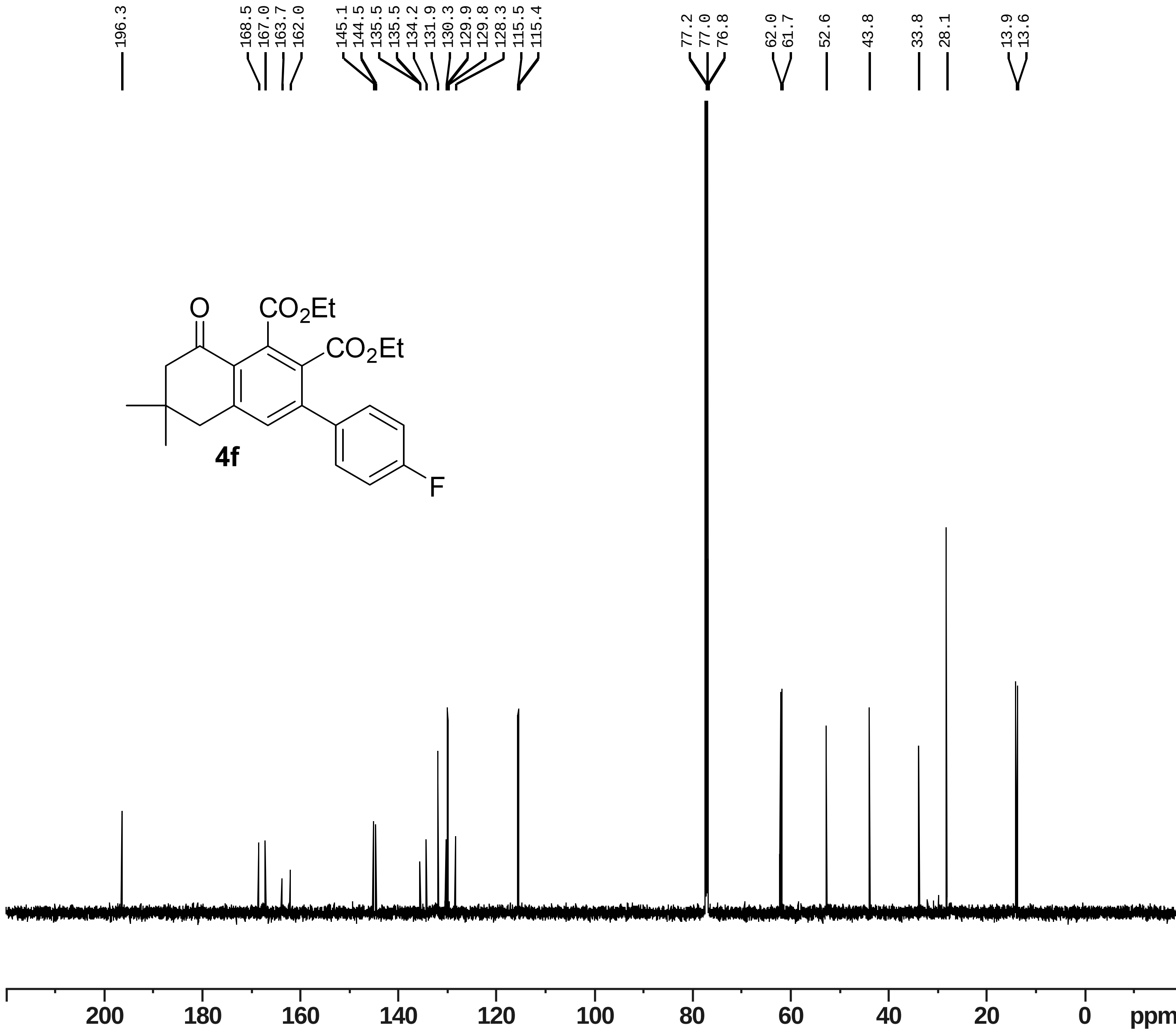

196.3
168.5
167.0
163.7
162.0
145.1
144.5
135.5
135.5
134.2
131.9
130.3
129.9
129.8
128.3
115.5
115.4
77.2
77.0
76.8
62.0
61.7
52.6
43.8
33.8
28.1
13.9
13.6
O
CO2Et
CO2Et
F
4f
200
180
160
140
120
100
80
60
40
20
0
ppm

Spectrum Plot Report

Sample Name	IS-20	Rack Position		Instrument	Instrument 1	Acq Operator	PARTHA
Inj Vol (ul)	10	Plate Position		IRM Status	Success		
Data File	IS-20.d	Acq Method	APCI POS ION CDCL3 MS.m	Comment	413.1759	Acq Time (Local)	02-Jun-25 10:51:49 AM (UTC+04:00)

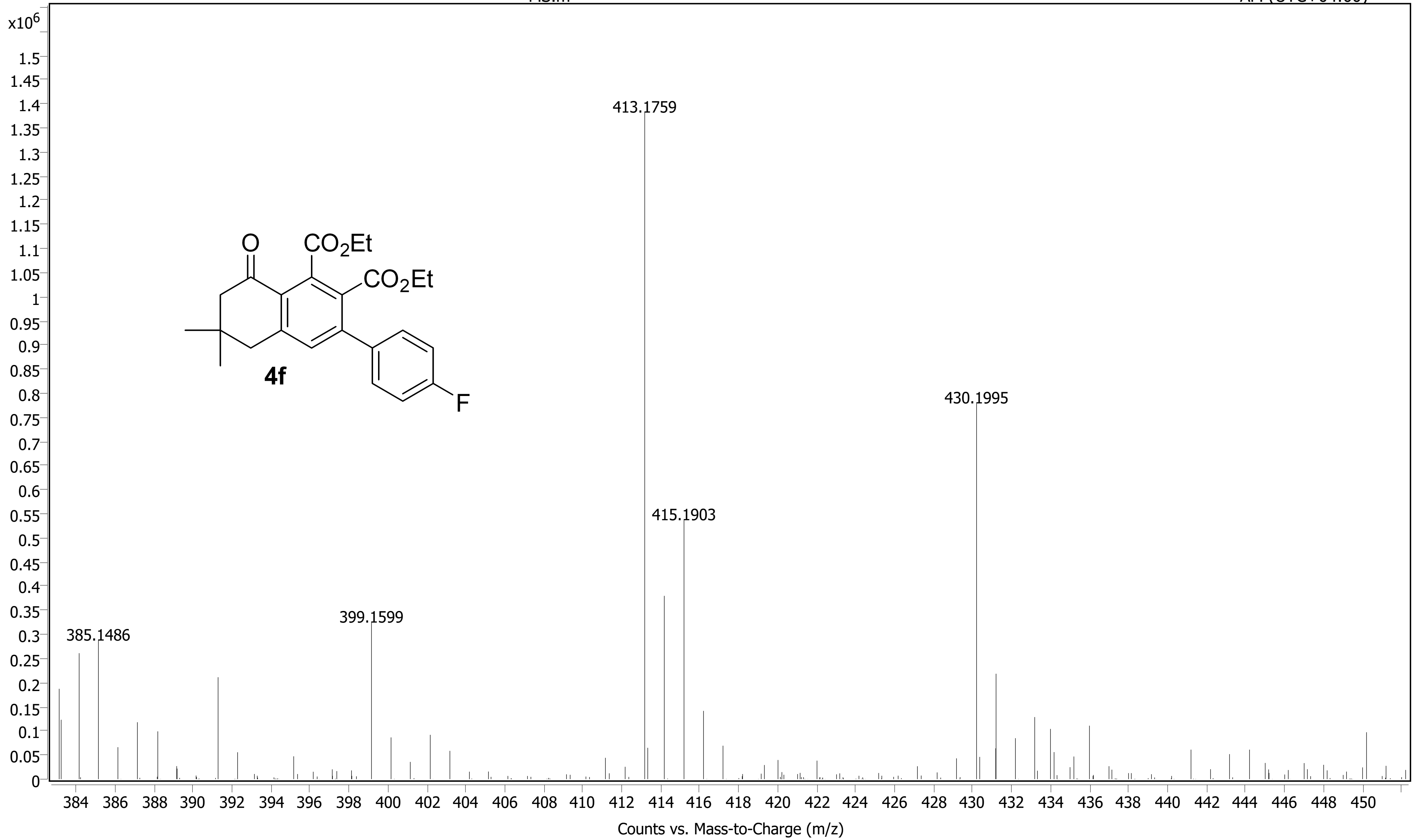

4f

Generated at 11:00 AM on 02-Jun-25

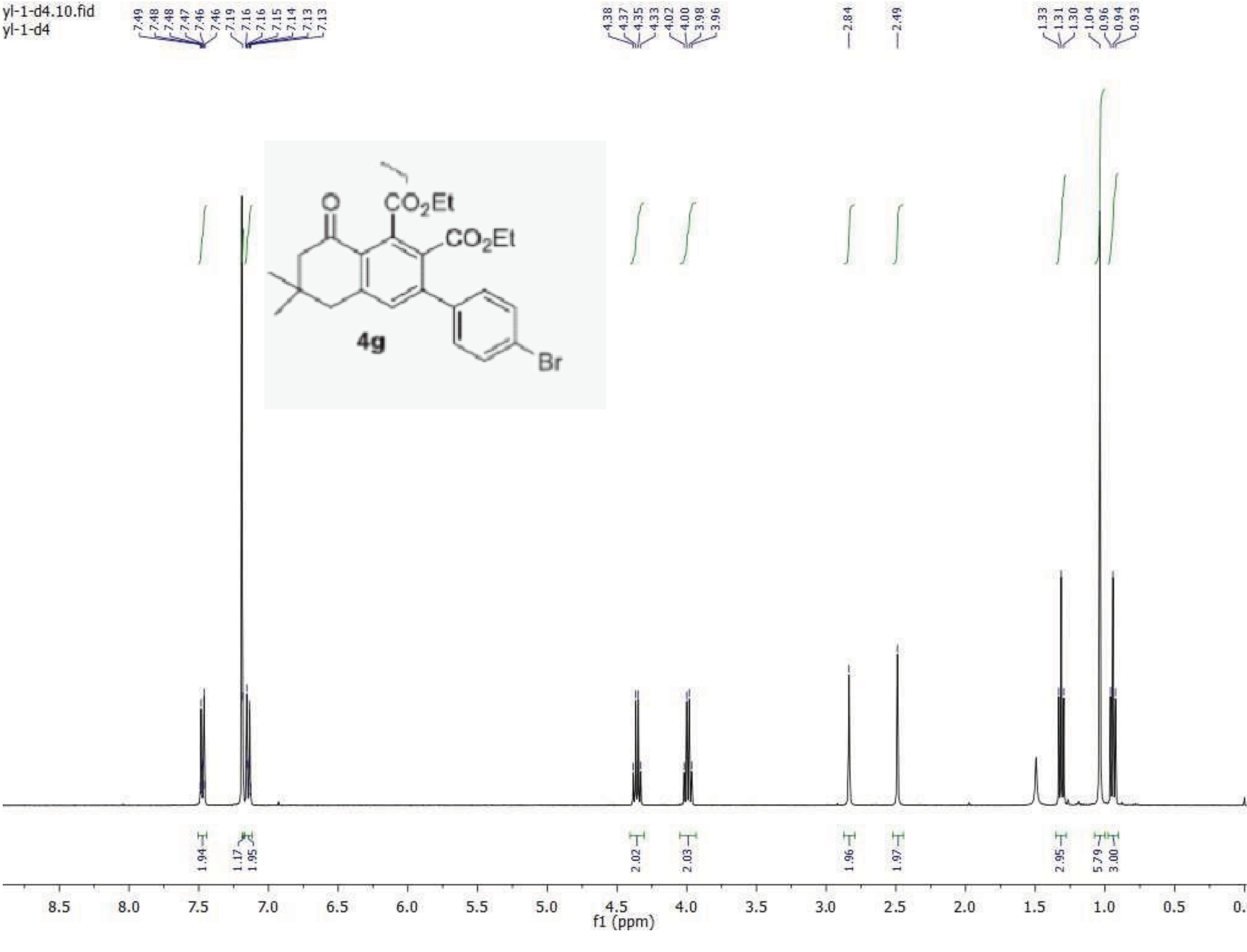

yl-1-d4.10.fid
yl-1-d4
7.49
7.48
7.48
7.47
7.46
7.46
7.19
7.16
7.16
7.15
7.14
7.13
7.13
4.38
4.37
4.35
4.33
4.02
4.00
3.98
3.96
2.84
2.49
1.33
1.31
1.30
1.04
0.96
0.94
0.93
CO2Et
CO2Et
4g
Br
1.94
1.17
1.95
2.02
2.03
1.96
1.97
2.95
5.79
3.00
8.5
8.0
7.5
7.0
6.5
6.0
5.5
5.0
4.5
4.0
3.5
3.0
2.5
2.0
1.5
1.0
0.5
f1 (ppm)

yl-d4.10.fid
yl-d4-C

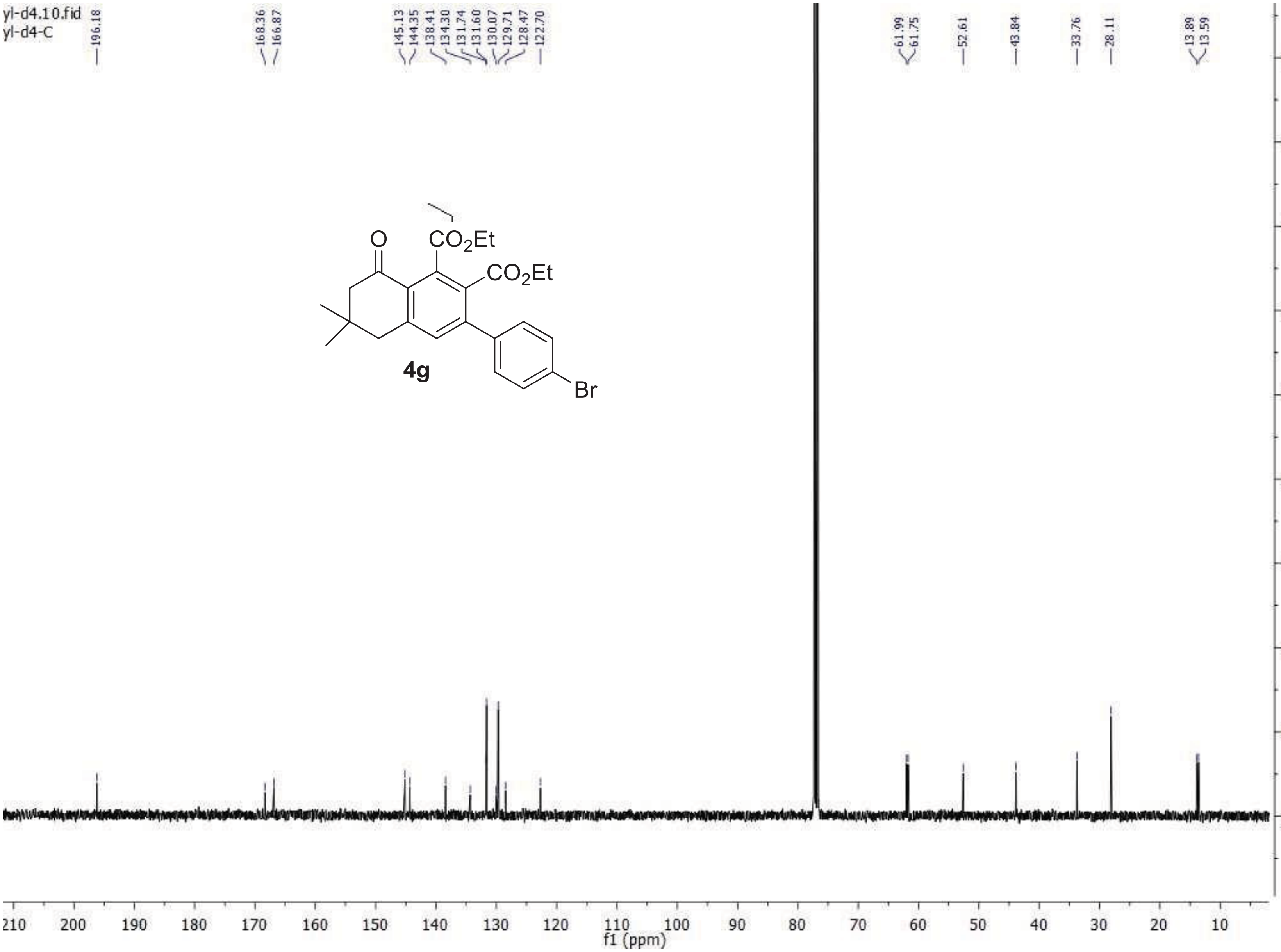

Spectrum Plot Report

Sample Name	IS-11	Rack Position		Instrument	Instrument 1	Acq Operator	PARTHA
Inj Vol (ul)	10	Plate Position		IRM Status	Success		
Data File	IS-11.d	Acq Method	APCI POS ION MEOH MS.m	Comment	473.0964	Acq Time (Local)	29-Apr-25 3:24:35 PM (UTC+04:00)

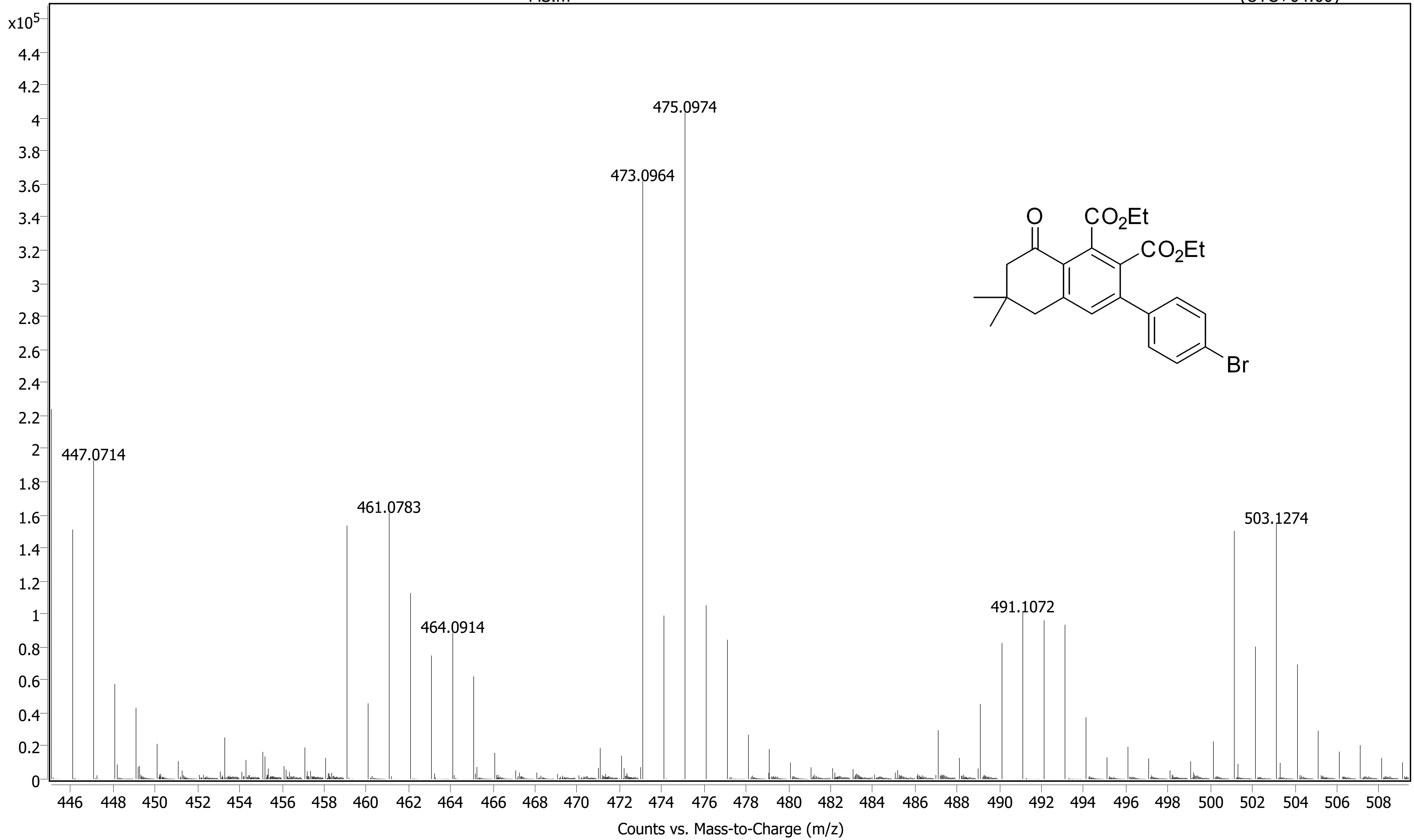

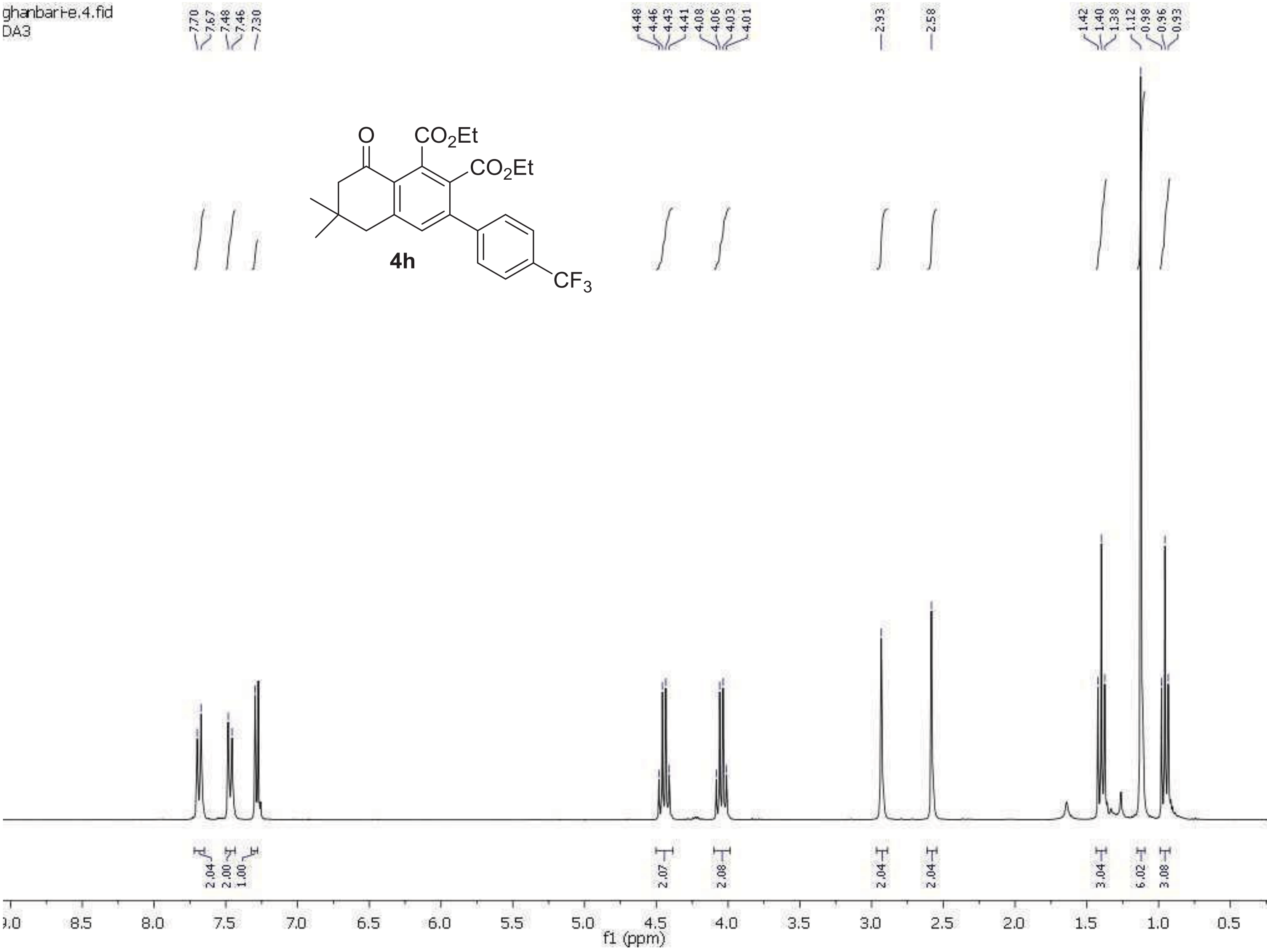
ghanbari-e.4.fid
DA3
7.70 7.67 7.48 7.46 7.30
4.48 4.46 4.43 4.41 4.08 4.06 4.03 4.01
2.93
2.58
1.42 1.40 1.38 1.12 0.98 0.96 0.93
O
CO2Et
CO2Et
4h
CF3
2.04 2.00 1.00 2.07 2.08 2.04 2.04 3.04 6.02 3.08
9.0 8.5 8.0 7.5 7.0 6.5 6.0 5.5 5.0 4.5 4.0 3.5 3.0 2.5 2.0 1.5 1.0 0.5
f1 (ppm)

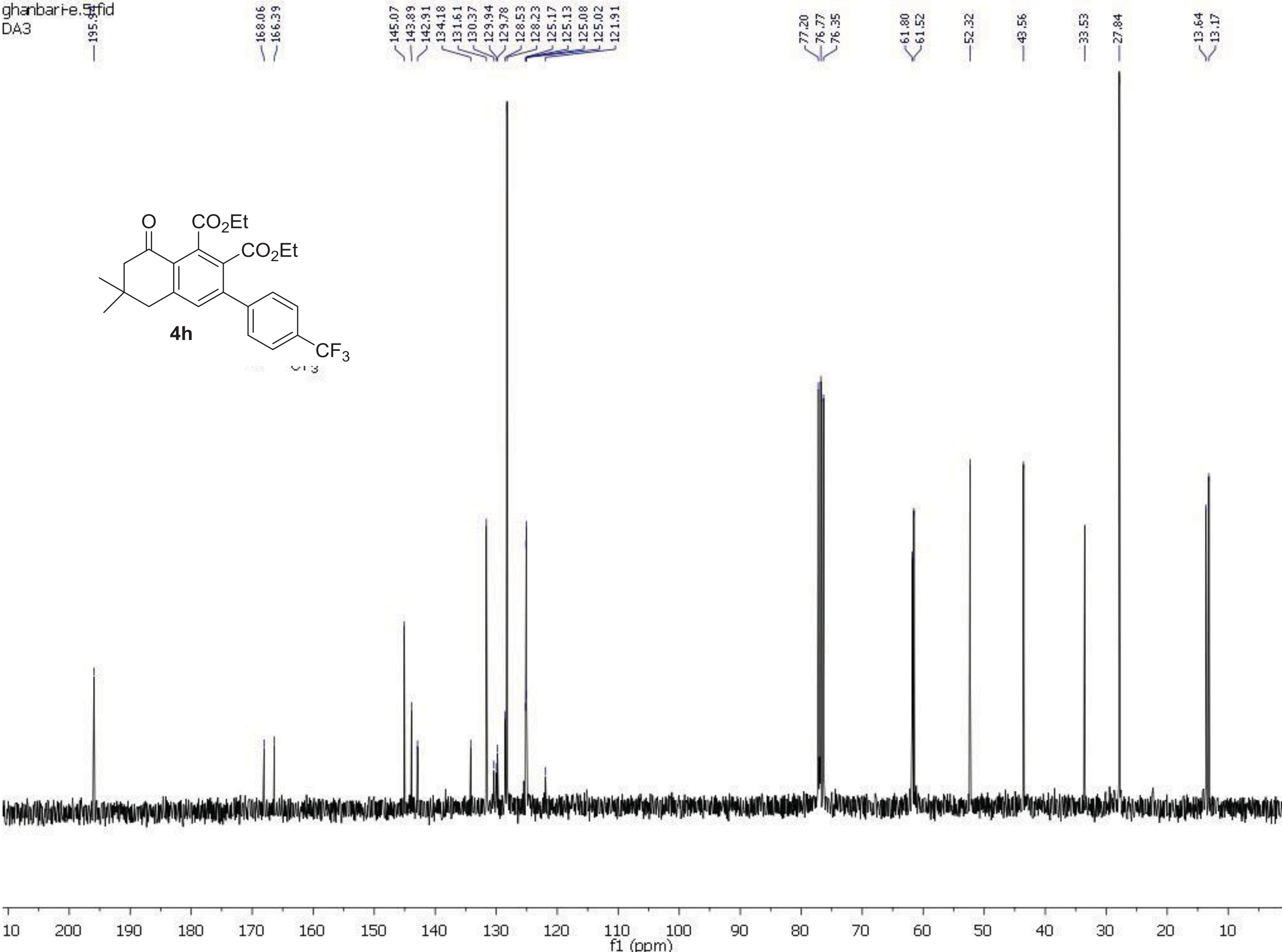

DA3
168.06
166.39
145.07
143.89
142.91
134.18
131.61
130.37
129.94
129.78
128.53
128.23
125.17
125.13
125.08
125.02
121.91
77.20
76.77
76.35
61.80
61.52
52.32
43.56
33.53
27.84
13.64
13.17
O
CO2Et
CO2Et
CF3
4h
f1 (ppm)

Spectrum Plot Report

Sample Name	IS-13	Rack Position		Instrument	Instrument 1	Acq Operator	PARTHA
Inj Vol (ul)	10	Plate Position		IRM Status	Success		
Data File	IS-13.d	Acq Method	APCI POS ION MEOH MS.m	Comment	463.1732	Acq Time (Local)	29-Apr-25 3:35:33 PM (UTC+04:00)

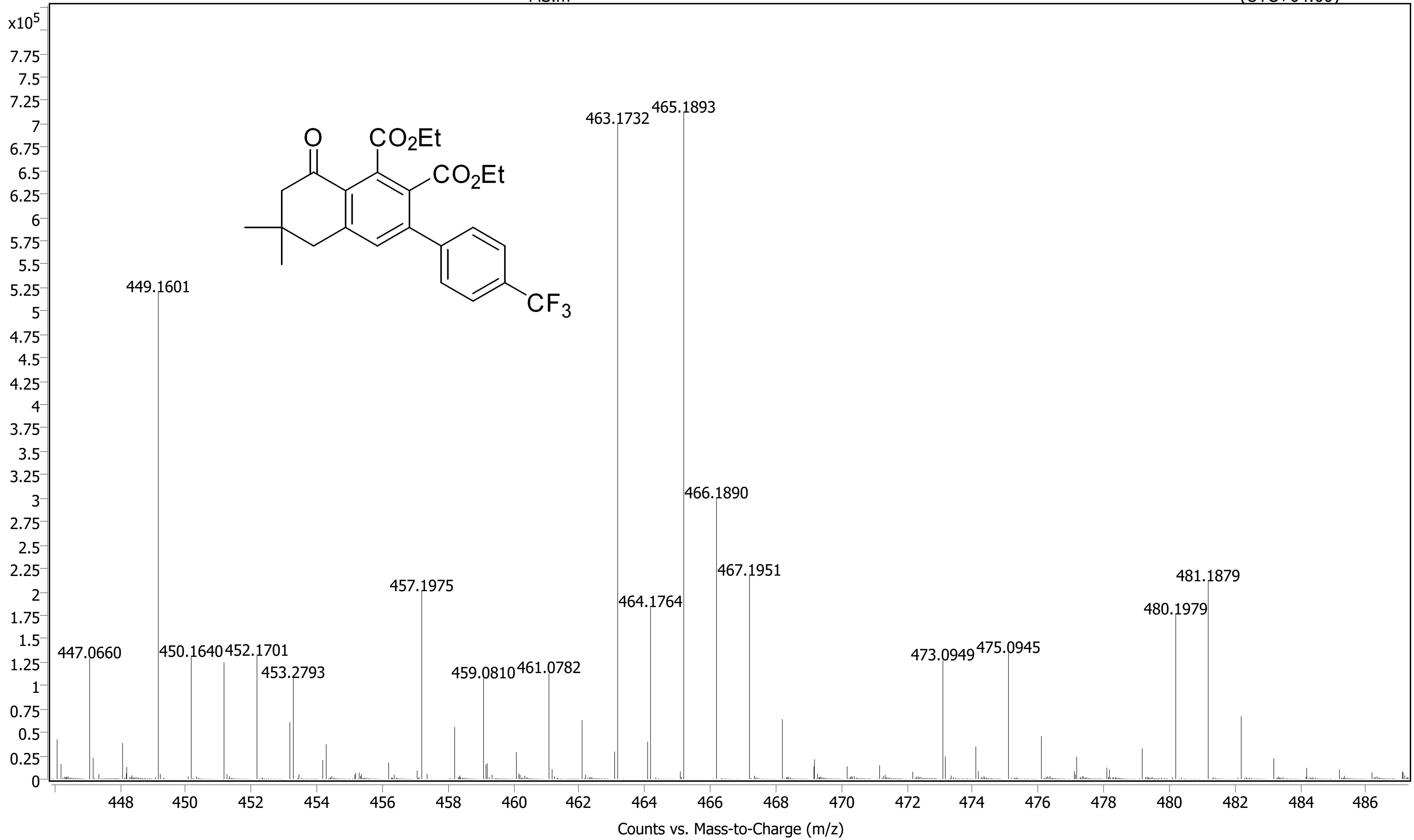

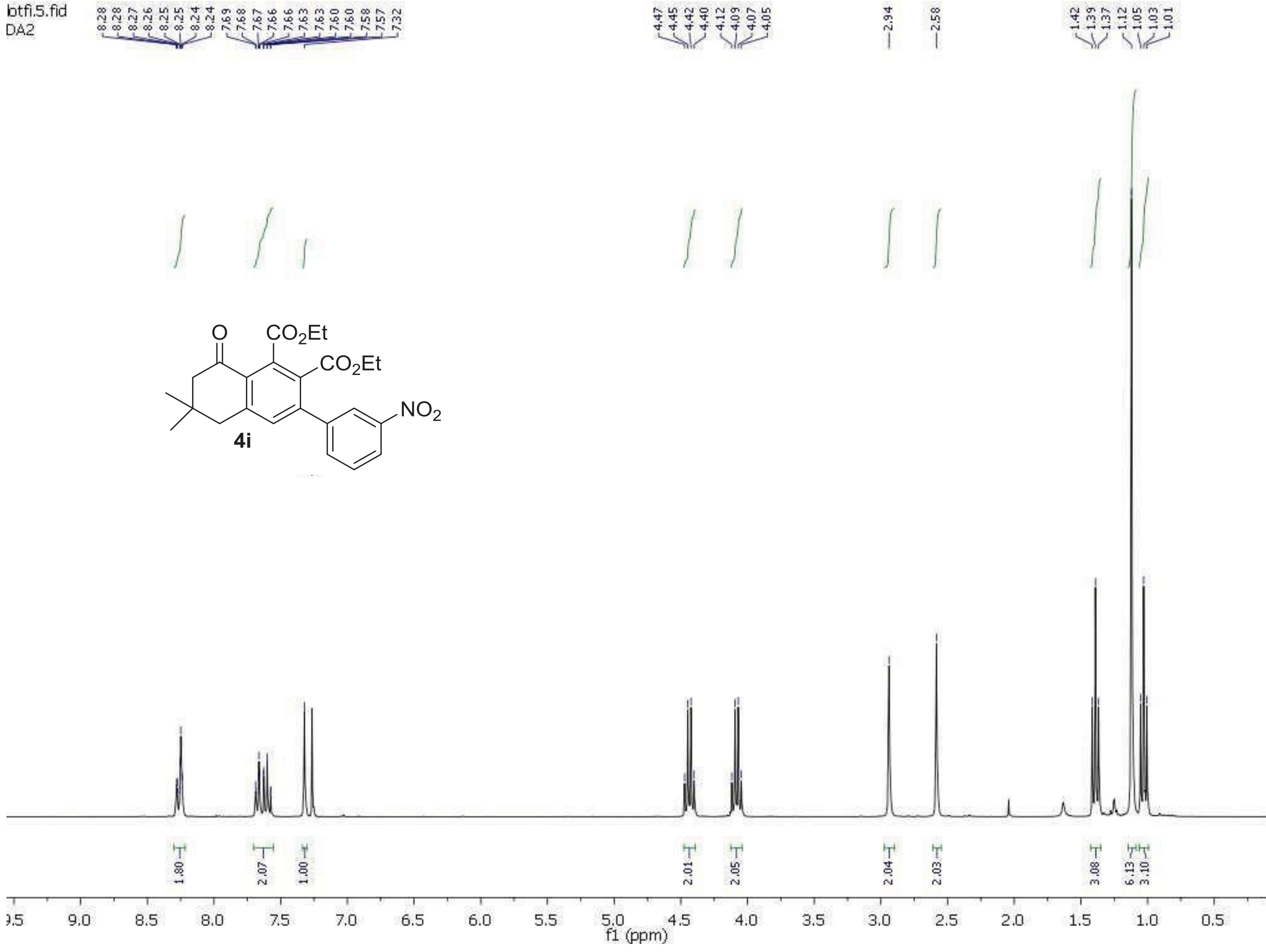

btfi.5.fid
DA2
8.28 8.28 8.27 8.26 8.25 8.25 8.24 8.24 7.69 7.68 7.67 7.66 7.66 7.63 7.63 7.60 7.60 7.58 7.57 7.32
4.47 4.45 4.42 4.40 4.12 4.09 4.07 4.05
2.94
2.58
1.42 1.39 1.37 1.12 1.05 1.03 1.01
O
CO2Et
CO2Et
NO2
4i
1.80
2.07
1.00
2.01
2.05
2.04
2.03
3.08
6.13
3.10
9.5 9.0 8.5 8.0 7.5 7.0 6.5 6.0 5.5 5.0 4.5 4.0 3.5 3.0 2.5 2.0 1.5 1.0 0.5
f1 (ppm)

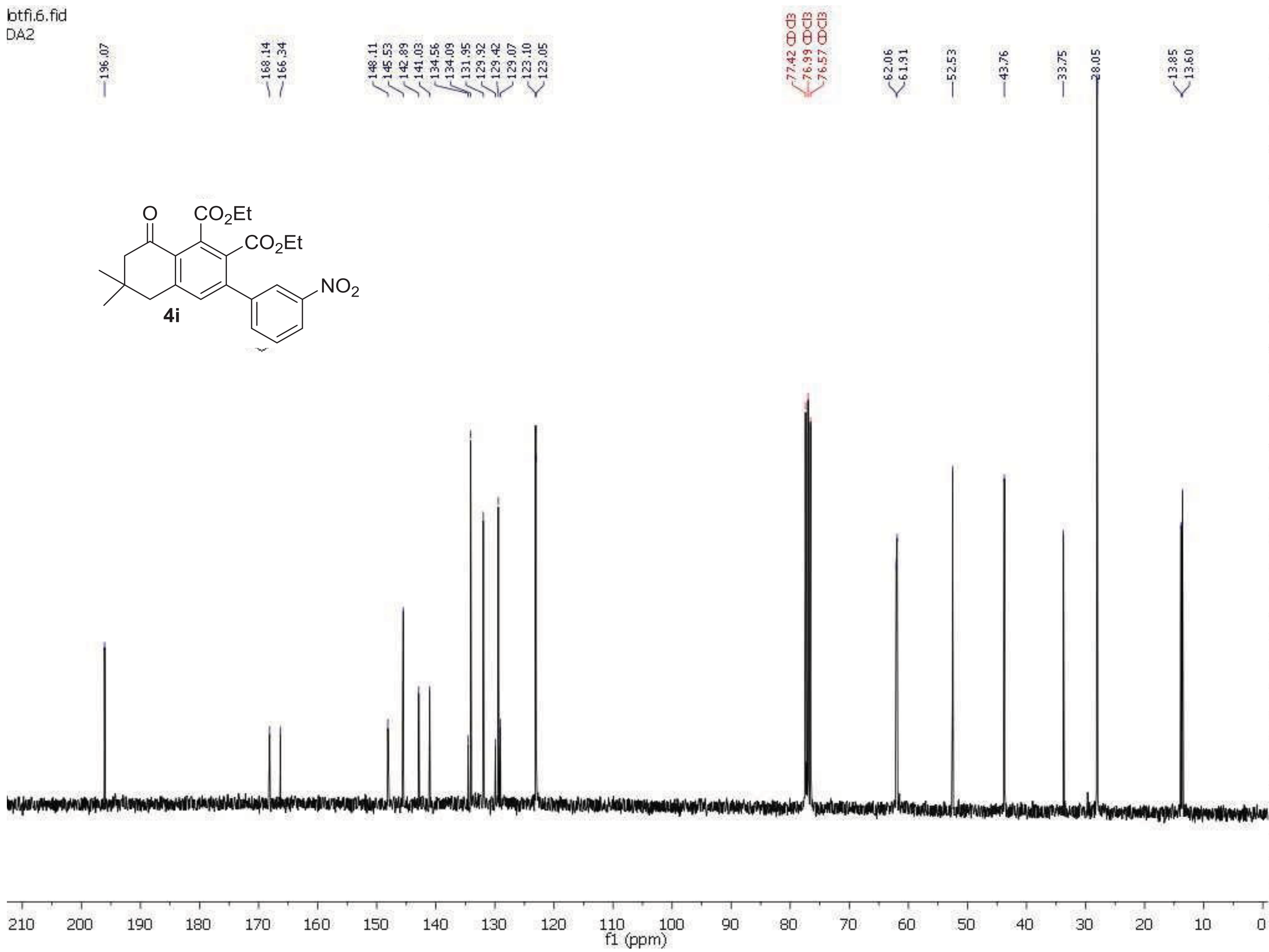
btfi.6.fid
DA2
196.07
168.14
166.34
148.11
145.53
142.89
141.03
134.56
134.09
131.95
129.92
129.42
129.07
123.10
123.05
77.42 CDCl3
76.99 CDCl3
76.57 CDCl3
62.06
61.91
52.53
43.76
33.75
28.05
13.85
13.60
O
CO_2Et
CO_2Et
NO_2
4i
210 200 190 180 170 160 150 140 130 120 110 100 90 80 70 60 50 40 30 20 10 0
f1 (ppm)

Spectrum Plot Report

Agilent

Sample Name	IS-12	Rack Position		Instrument	Instrument 1	Acq Operator	PARTHA
Inj Vol (ul)	10	Plate Position		IRM Status	Success		
Data File	IS-12.d	Acq Method	APCI POS ION MEOH MS.m	Comment	440.1709	Acq Time (Local)	29-Apr-25 3:29:58 PM (UTC+04:00)

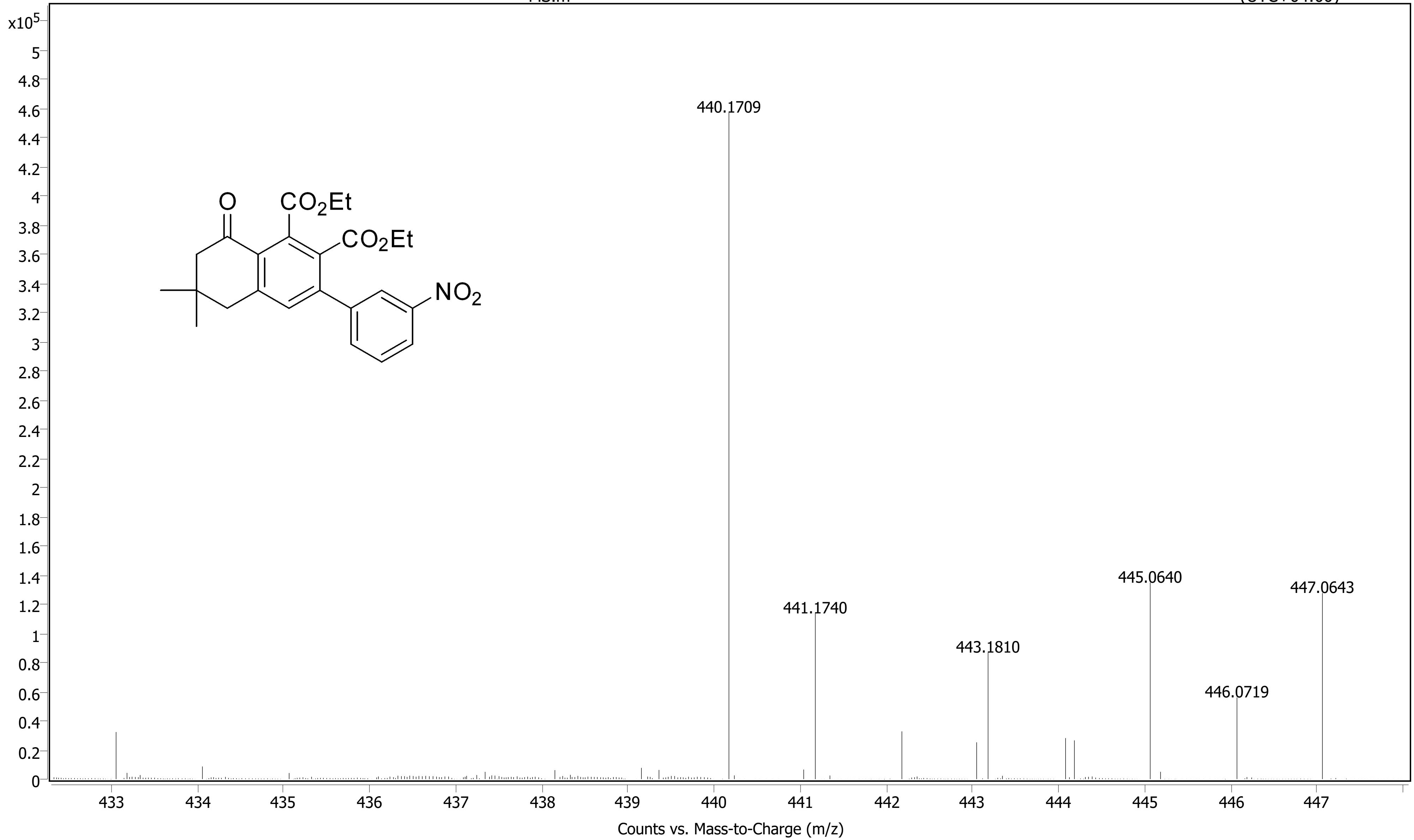

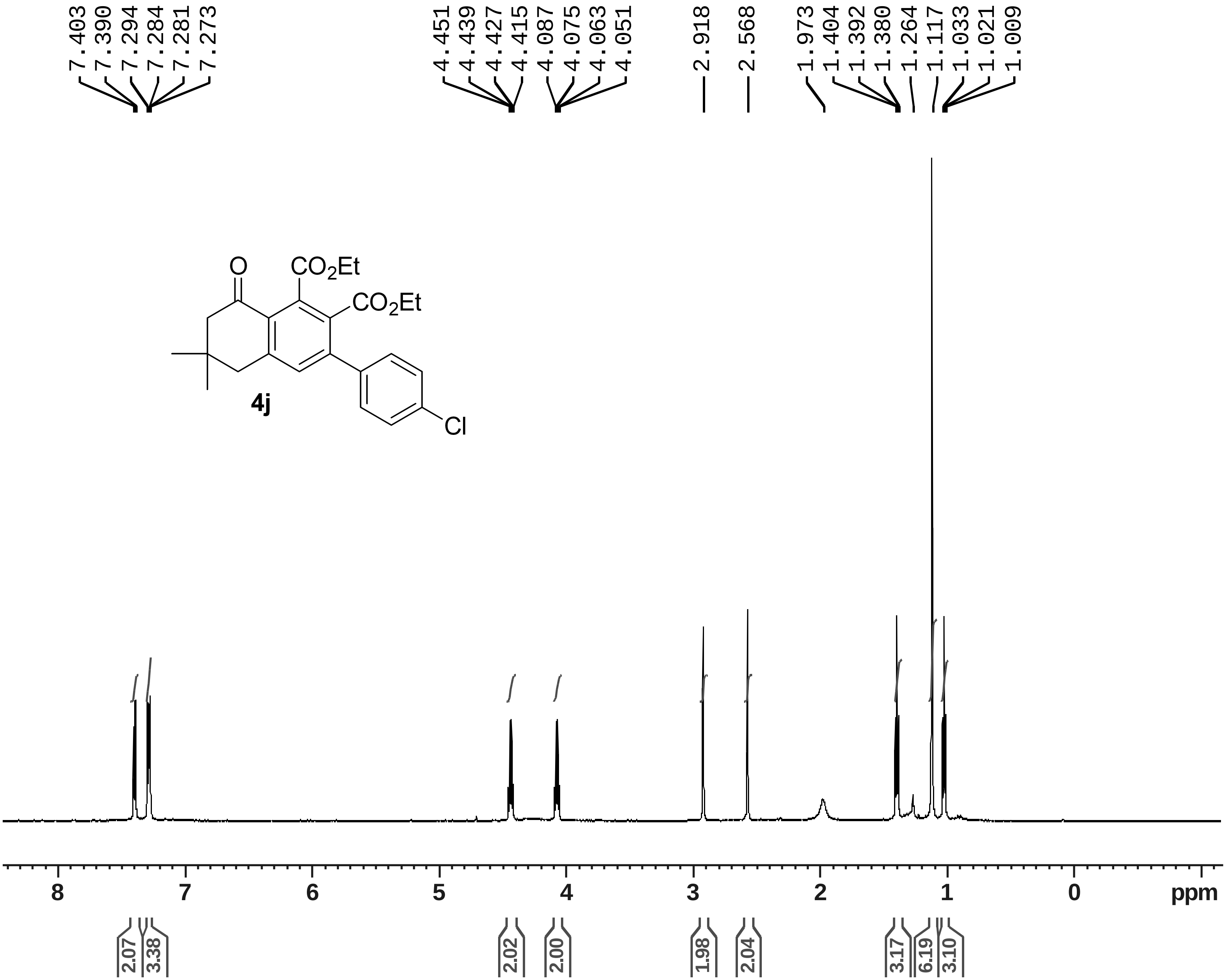

7.403
7.390
7.294
7.284
7.281
7.273
4.451
4.439
4.427
4.415
4.087
4.075
4.063
4.051
2.918
2.568
1.973
1.404
1.392
1.380
1.264
1.117
1.033
1.021
1.009
O
CO2Et
CO2Et
Cl
4j
8
7
6
5
4
3
2
1
0
ppm
2.07
3.38
2.02
2.00
1.98
2.04
3.17
6.19
3.10

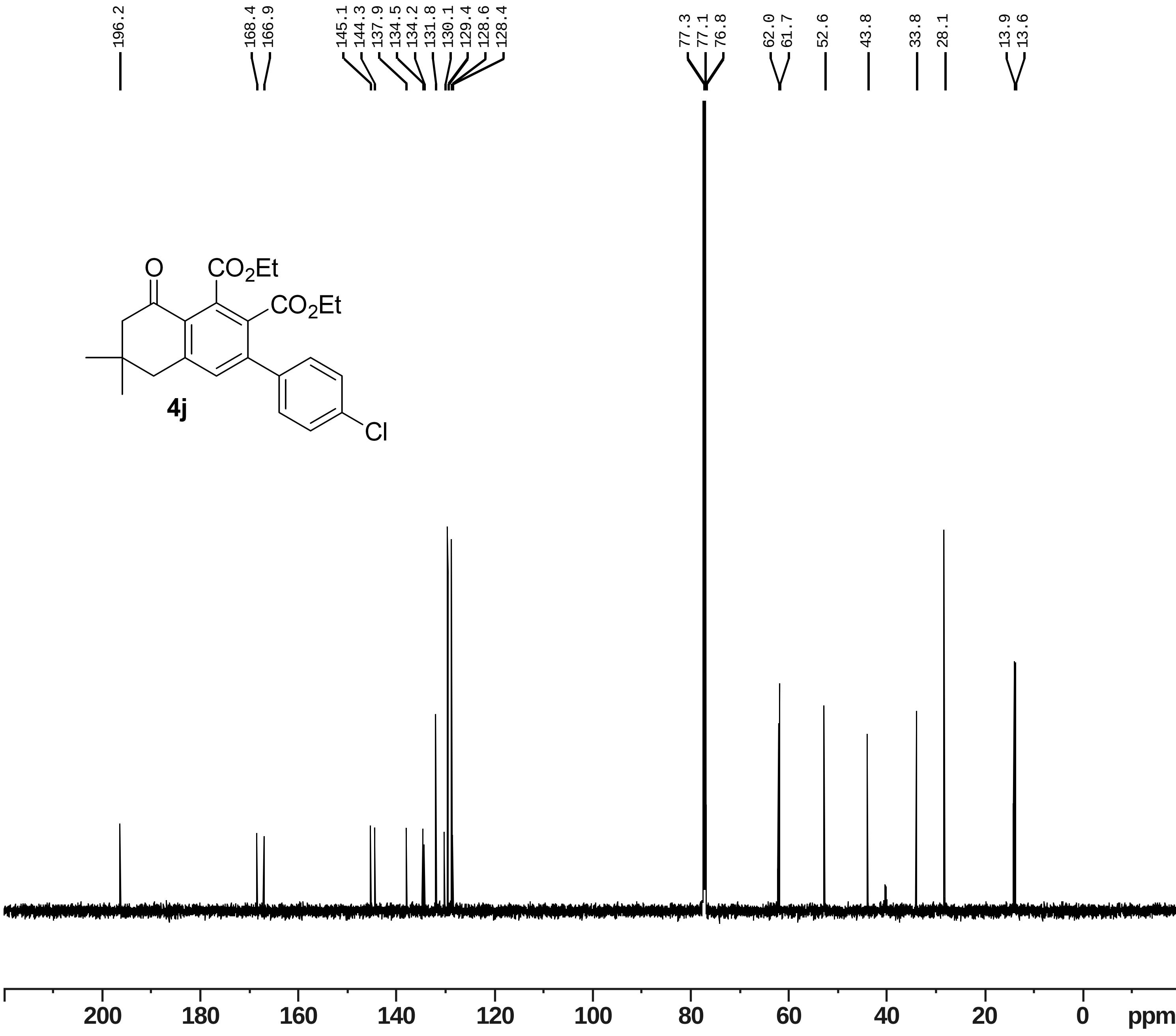
196.2
168.4
166.9
145.1
144.3
137.9
134.5
134.2
131.8
130.1
129.4
128.6
128.4
77.3
77.1
76.8
62.0
61.7
52.6
43.8
33.8
28.1
13.9
13.6
O
CO_2Et
CO_2Et
Cl
4j
200
180
160
140
120
100
80
60
40
20
0
ppm

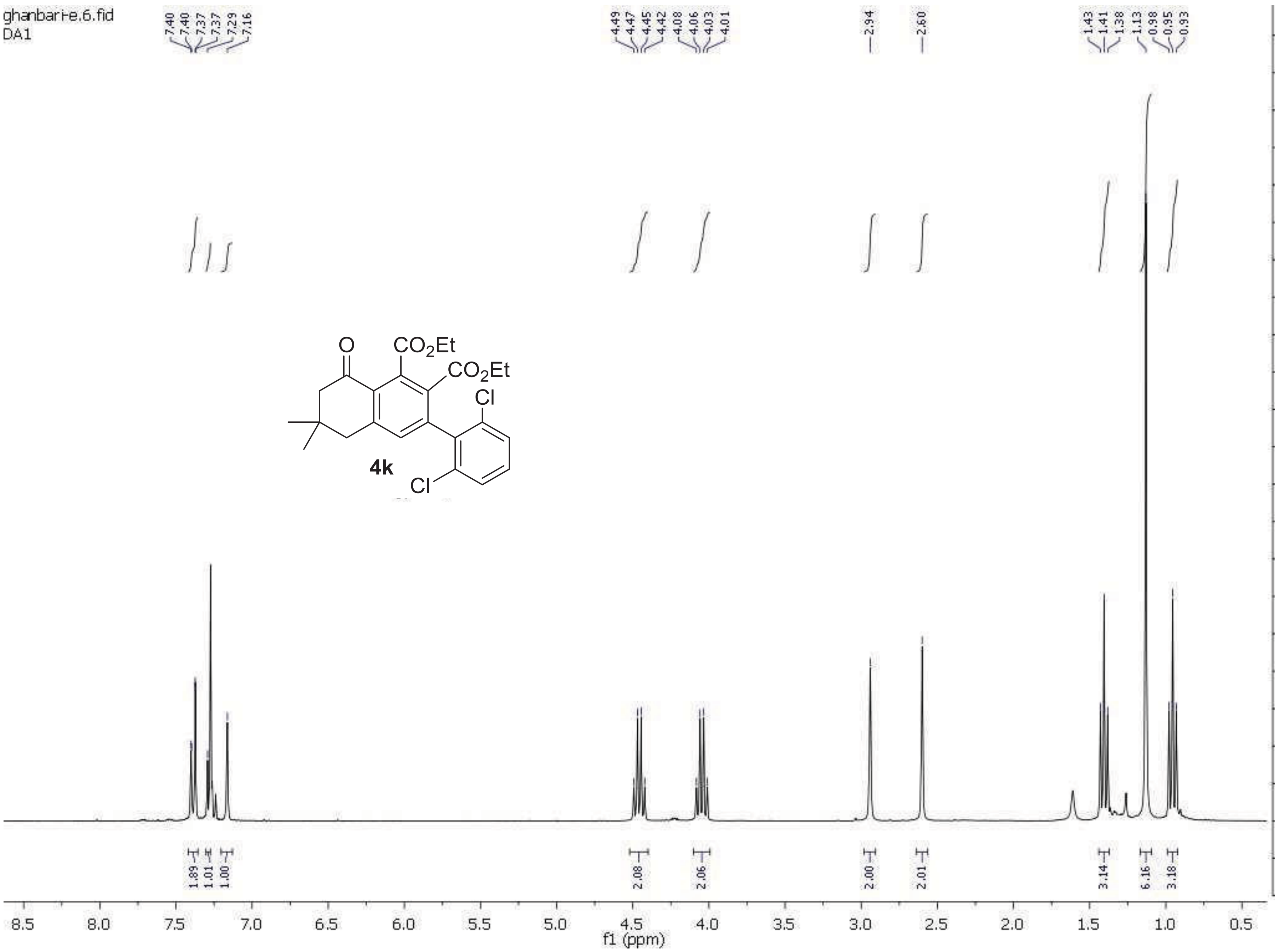

ghanbari-e.6.fid
DA1
7.40 7.40 7.37 7.37 7.29 7.16
4.49 4.47 4.45 4.42 4.08 4.06 4.03 4.01
2.94
2.60
1.43 1.41 1.38 1.13 0.98 0.95 0.93
O
CO2Et
CO2Et
Cl
Cl
4k
1.89 1.01 1.00 2.08 2.06 2.00 2.01 3.14 6.16 3.18
8.5 8.0 7.5 7.0 6.5 6.0 5.5 5.0 4.5 4.0 3.5 3.0 2.5 2.0 1.5 1.0 0.5
f1 (ppm)

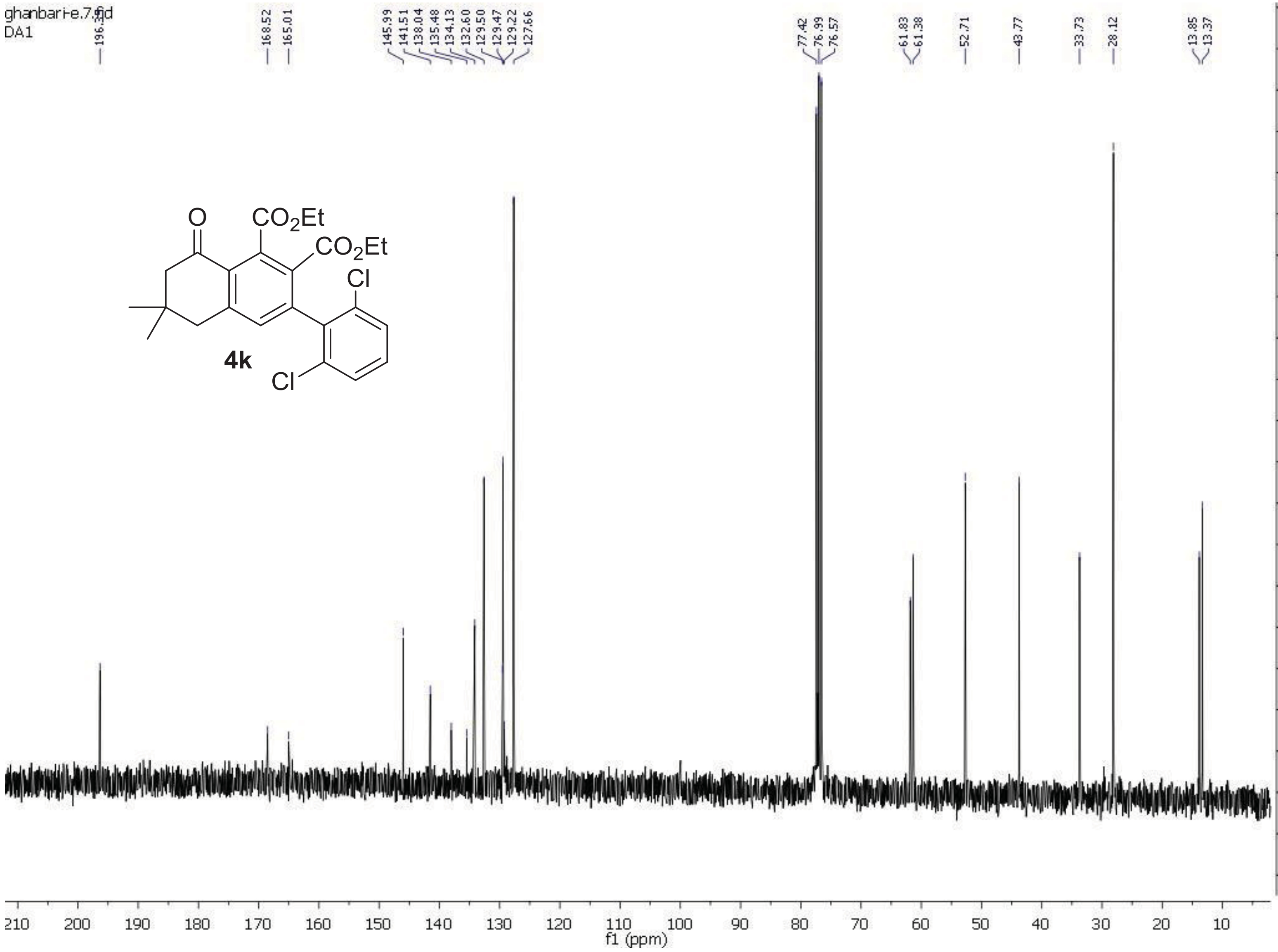
ghanbari-e.7.fid
DA1
168.52
165.01
145.99
141.51
138.04
135.48
134.13
132.60
129.50
129.47
129.22
127.66
77.42
76.99
76.57
61.83
61.38
52.71
43.77
33.73
28.12
13.85
13.37
O
CO2Et
CO2Et
Cl
Cl
4k
210 200 190 180 170 160 150 140 130 120 110 100 90 80 70 60 50 40 30 20 10
f1 (ppm)

Spectrum Plot Report

Sample Name	IS-14	Rack Position		Instrument	Instrument 1	Acq Operator	PARTHA
Inj Vol (ul)	10	Plate Position		IRM Status	Success		
Data File	IS-14.d	Acq Method	APCI POS ION MEOH MS.m	Comment	463.1079	Acq Time (Local)	29-Apr-25 3:40:53 PM (UTC+04:00)

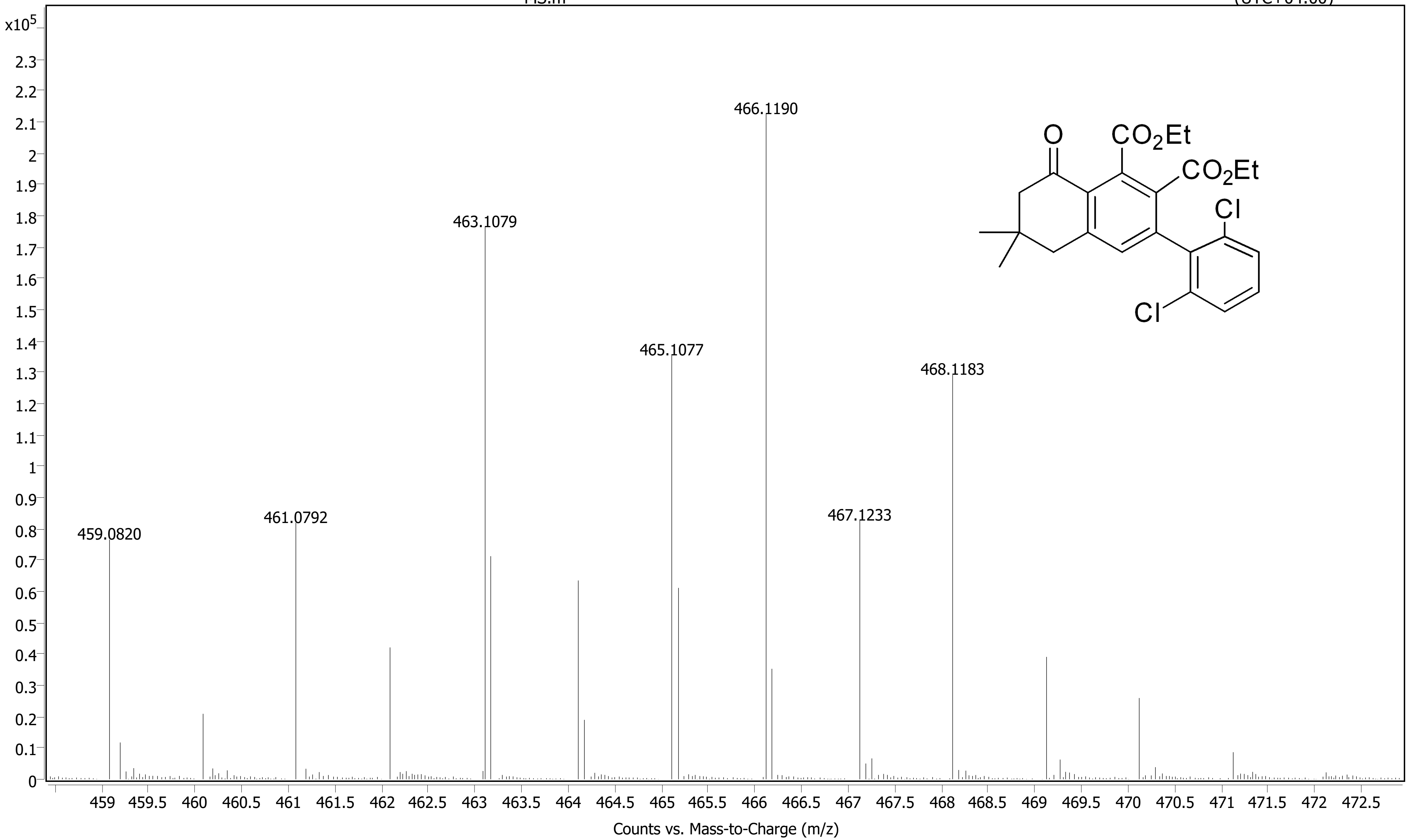

Generated at 9:09 AM on 30-Apr-25

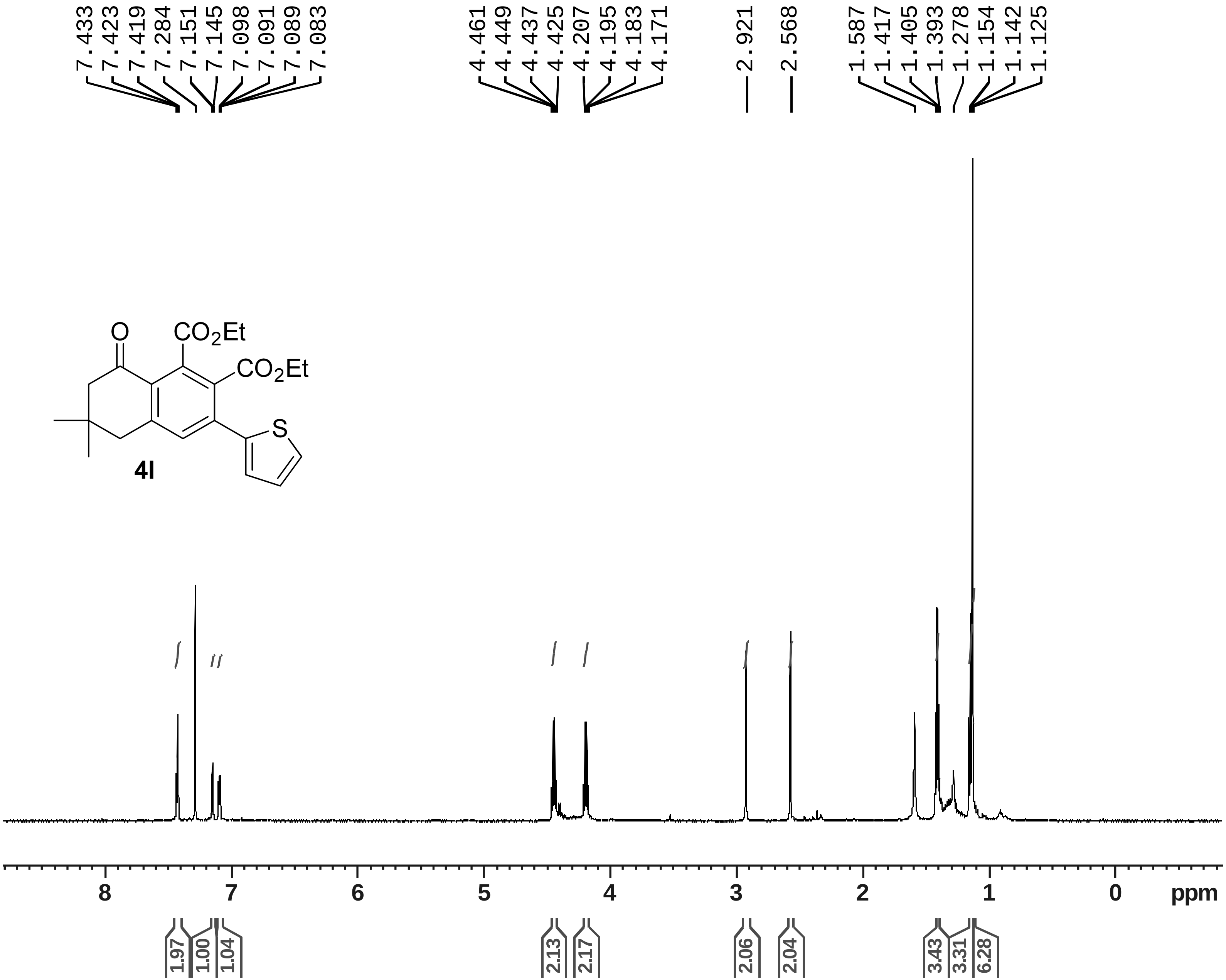
7.433 7.423 7.419 7.284 7.151 7.145 7.098 7.091 7.089 7.083
4.461 4.449 4.437 4.425 4.207 4.195 4.183 4.171
2.921
2.568
1.587 1.417 1.405 1.393 1.278 1.154 1.142 1.125
O
CO2Et
CO2Et
S
4l
8 7 6 5 4 3 2 1 0 ppm
1.97 1.00 1.04
2.13 2.17
2.06
2.04
3.43 3.31 6.28

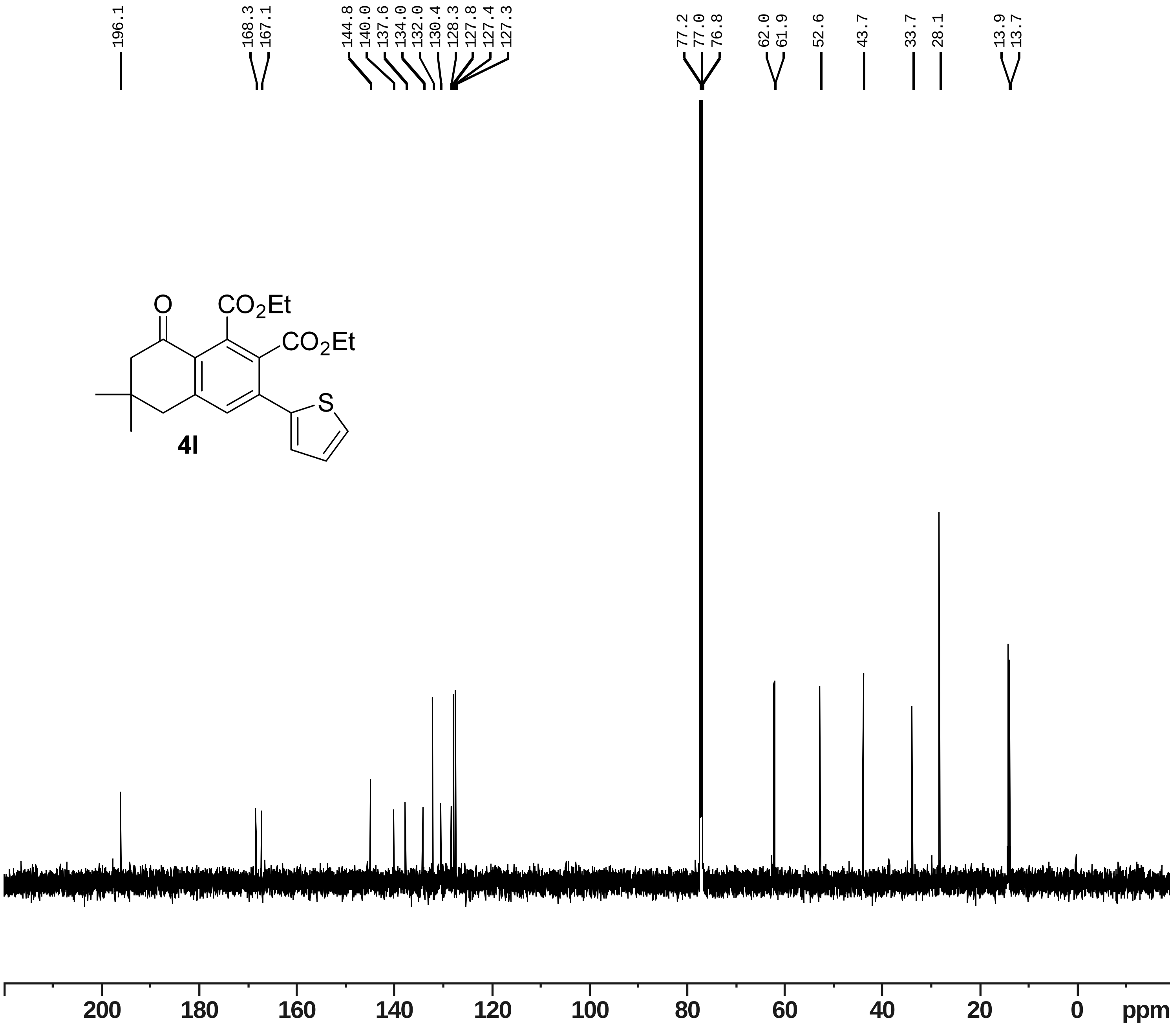
196.1
168.3
167.1
144.8
140.0
137.6
134.0
132.0
130.4
128.3
127.8
127.4
127.3
77.2
77.0
76.8
62.0
61.9
52.6
43.7
33.7
28.1
13.9
13.7
O
CO2Et
CO2Et
S
4l
200
180
160
140
120
100
80
60
40
20
0
ppm
